# Modifying Glucose Metabolism Reverses Memory Defects of Alzheimer's Disease Model at Late Stages

**DOI:** 10.1002/advs.202506695

**Published:** 2025-12-08

**Authors:** Fang Liu, Yiyu‐Li Tang, Zong‐Bo Zhang, Ya‐Hong Tan, Si‐Hui Lin, Ni‐Ya Wang, Jin‐Nan Li, Zhi‐Jie Pan, Jian‐Feng Li, Jing‐Fei Huang, Yu‐Qiang Ding, Chun‐Ming Guo, Lin Xu, Cheng Peng, Qi‐Xin Zhou

**Affiliations:** ^1^ Key Laboratory of Animal Models and Human Disease Mechanisms KIZ‐SU Joint Laboratory of Animal Model and Drug Development and Laboratory of Learning and Memory Kunming Institute of Zoology the Chinese Academy of Sciences Kunming 650223 China; ^2^ Yunnan Key Laboratory of Cell Metabolism and Disease and Center for Life Sciences School of Life Sciences Yunnan University Kunming 650500 China; ^3^ School of Chinese Materia Medica and Yunnan Key Laboratory of Southern Medicinal Utilization Yunnan Chinese Medicine University Kunming 650500 China; ^4^ State Key Laboratory of Medical Neurobiology and MOF Frontiers Center for Brain Science Institute of Brain Science and Department of Laboratory Animal Science Fudan University Shanghai 200032 China

**Keywords:** aging, Alzheimer's disease, Glut1, spatial transcriptomics

## Abstract

Significant efforts have harvested a sophisticated understanding of Alzheimer's disease (AD) including amyloid beta (Aβ) cascade mechanisms, although effective treatment for reversing or stopping AD progression is not available. This study reports that ferul enanthate (SL), a novel derivative of active agents targeting brain microvessels, oxidative phosphorylation, and ATP generation can reverse the hippocampus‐dependent spatial memory defects and reduce Aβ plaques in AD model mice (APP/PS1) at advanced stages. Spatial transcriptomics discovers that SL endows a cluster of genes expressing in Aging‐AD‐Rescue (AAR) pattern, which is prominent in hippocampal dendritic region where Aβ plaques are densely deposited. Furthermore, this AAR rule covers hippocampal Glut1 (glucose transporter 1) expression and ATP generation, which are further confirmed by immunoblotting or immunofluorescence studies. Our data demonstrate that SL can still reverse memory defects at advanced stages of AD mice by modifying aging‐dependent multiple pathologies of AD, particularly promoting Glut1 expression and ATP generation.

## Graphical abstract

Using spatial transcriptomics, we show that ferul enanthate (SL‐ZF‐01) reverses episodic‐like memory deficits in aged, but not young, Alzheimer’s disease (AD) mice. SL restores glucose metabolism and Glucose Transporter 1/3 expression via an ‘Aging‐AD‐Rescue’ pattern, rescuing deficits seen in aged AD mice. This suggests late‐stage AD progression may be reversed by targeting ageing‐dependent pathways.

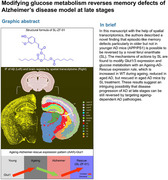

## 1 Introduction

Alzheimer's disease (AD) has been well recognized as a neurodegenerative disease, characterized by the pathological hallmarks of Aβ plaques and tau tangles as well as progressive decline of episodic memory and other cognitive functions.^[^ ^1^, ^2^, ^3^ ^]^ Recent progress demonstrates that Aβ‐targeting monoclonal antibodies can slow down the progression of early AD patients,^[^ ^4^ ^]^ a big step forward for disease‐modifying therapy (DMT). Yet, no drugs are available for effectively treating AD at advanced stages when the patients are usually females and older.

Aging is a major risk factor of AD. Young people with AD‐associated mutations do not suffer AD until 40 or 50 years old, while AD prevalence shoots up from 65 to 85 years old, almost doubling every 5 years.^[^ ^5^ ^]^ Although aging alone is possibly not sufficient for causing AD, even in those with an obvious load of Aβ plaques,^[^ ^6^ ^]^ it can interact with Aβ pathologies,^[^ ^7^ ^]^ leading to reduced cerebral blood flow,^[^ ^8^ ^]^ microvascular abnormalities,^[^ ^9^ ^]^ and impaired brain glucose metabolism,^[^ ^10^ ^]^ eventually pushing forward the AD progression.^[^ ^11^ ^]^ In particularly, brain glucose metabolism is essential for neuronal function and viability. The insulin‐independent glucose transporters Glut1 and Glut3, expressed respectively in endothelial/glial cells and neurons, are crucial for maintaining cerebral glucose supply.^[^ ^12^, ^13^ ^]^ Past study reports diminished Glut1 expression in the brains of AD patients,^[^ ^12^ ^]^ and Glut1 knocked down in AD model mice (APP) exacerbates cognitive defects and Aβ plaque deposition.^[^ ^14^ ^]^ These findings align with the “brain energy rescue” concept, wherein the maintenance of brain glucose uptake and metabolism serves as a key adaptive mechanism in healthy aging that fails in AD.^[^ ^15^, ^16^ ^]^ Therefore, we propose that adaptive mechanisms under healthy **Aging** condition are lost under **AD** aging condition but that can be ideally **Rescued** by certain treatments (termed **AAR** hypothesis).

Previous reports suggest that ferulic acid improves spatial memory in early‐stage AD mouse models,^[^ ^17^, ^18^ ^]^ while enanthic acid (or heptanoic acid) aids supports oxidative phosphorylation and ATP generation.^[^ ^19^, ^20^, ^21^ ^]^ To prove the concept, we linked these two molecules via an ester bond to form a novel compound (SL‐ZF‐01 or simply SL). We found that SL treatment had little effects in adult mice (males and females) but improved spatial memory in aged females (APP/PS1 mice) via enhancing Glut1 expression, which matches our AAR hypothesis (increased expression in WT mice during aging, but decreased expression in AD mice during aging that was rescued by SL treatment), to against AD pathologies. Our study suggests that SL targeting Glut1 may potentially serve as a novel therapy for female AD at advanced stages.

## 2 Results

### 2.1 SL and its Efficacy on Spatial Memory Defects in AD Mice

**Figure 1:**
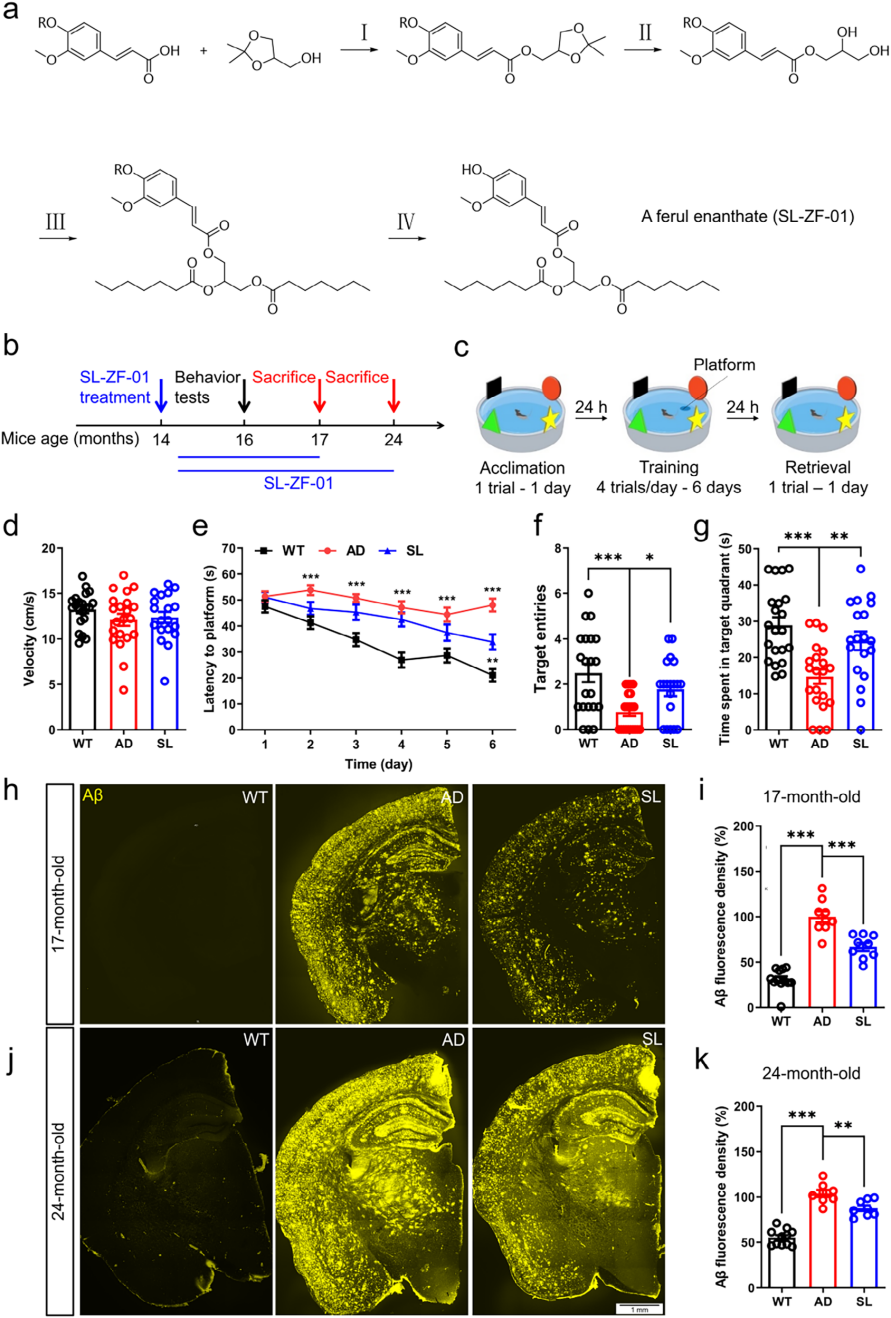
SL reversed spatial memory defects in AD mice at late stages. a) One of the ferul enanthate derivatives, SL‐ZF‐01 (SL), synthesized in four‐steps from ferulic acid and enanthic acid (active agents), for achieving multitarget action on AD. b) Experimental design on SL treatment and behavioral test. The AD mice were fed with normal pellets + SL from 14‐month‐old to 17‐or 24‐month‐old, for which SL mice were termed; The age‐matched AD and wildtype (WT) mice were fed with normal pellets. c) The spatial learning task of the Morris water maze was tested in WT, AD, and SL groups at 16‐month‐old. d) Swimming velocity measured during acclimation day 1 was not significantly different among groups(WT, black, *N* = 21; AD, red, N = 21; SL, blue, N = 19 for panels d–g). e) Spatial learning as indicated by gradually decreased latency in escaping onto a hidden platform during 6‐days training was impaired in AD mice but partially rescued in SL mice relative to WT mice. f,g) Spatial memory on day 7 as indicated by target entries and target quadrant time was impaired in AD mice but near completely rescued in SL mice relative to WT mice. h,j) Representative images of Aβ immunofluorescent staining in WT, AD, and SL groups at 17‐and 24‐month‐old. i,k) The normalized Aβfluorescence density, in which the AD group was used as reference, as the Aβ plaque loading was largely increased in AD mice at 17‐or 24‐month‐old but partially rescued in SL mice (17‐month‐old: WT, black, *N* = 4, *n* = 12; AD, red, *N* = 4, *n* = 9; SL, blue, *N* = 4, *n* = 9. 24‐month‐old: WT, black, *N* = 11; AD, red, *N* = 8; SL, blue, *N* =8). ^*^ *p* < 0.05; ^**^ *p* < 0.01; ^***^ *p* < 0.001 by two‐way ANOVA with Dunnett's multiple comparisons test (e), one‐way ANOVA with Tukey's multiple comparisons test (d,g,i,k), ANOVA followed VA followed by Dunnett's T3 multiple comparisons test (f). Data are presented as mean ± SEM. N, numbers of mice; n) numbers of slices. All mice used here were female.

Thus, we synthesized the ferul enanthate derivative SL by linking ferulic acid and enanthic acid through 4‐steps chemical reactions (**Figure** **1**a). The novel structure of SL was carefully checked by using 1H/13C nuclear magnetic resonance and mass spectrums (see Figure S1a–c, Supporting Information). This candidate was then tested in AD mice at different stages by using the spatial learning task of the Morris water maze (Figure 1b,c), which is a well‐known behavioral paradigm for testing the hippocampus‐dependent episodic‐like spatial memory.^[^ ^22^ ^]^ Preliminary studies were performed to have determined dosage, administration route, and treatment length of SL. One‐month treatment of SL in db/db mice (2‐ to 3‐month‐old) or in AD mice (8‐ to 9‐month‐old) had no significant effect on spatial memory (Figure S2a–e, Supporting Information for db/db; Figure S2f–n, Supporting Information for AD), suggesting that SL's action is negative relevant to either insulin resistance (Glucose transporter type 4 pathway) or early stages of AD characterized by mild memory defects and sparse Aβ plaques.^[^ ^18^ ^]^

Daily SL treatment for 2 months (from 14‐ to 16‐month‐old) in AD mice (SL group) did not affect swimming velocity in the water maze during acclimation on day 1 as compared to WT or AD group without SL treatment (Figure 1d). However, this treatment improved spatial learning during the 6‐day training period, as indicated by a significantly shorter latency in escaping onto the hidden platform on day 6 (AD vs. SL, *p* = 0.0018; Figure 1e). Notably, this treatment intriguingly improved spatial memory as indicated by more target entries (AD vs. SL, *p* = 0.0155; Figure 1f) and more time spent in target quadrant (AD vs. SL, *p* = 0.0060; Figure 1g), both of which were largely reduced in AD mice but near completely rescued to the WT levels in SL mice (Figure 1f,g). Some of SL mice (continued SL treatment), and age‐matched AD and WT mice were sacrificed at 17‐ and 24‐month‐old for immunofluorescent staining of Aβ plaque (Figure 1h,i). Aβ plaque loading as indicated by Aβ fluorescence density was largely increased in AD mice but partly reduced in SL mice at both the ages (17‐ and 24‐month‐old) (Figure 1j,k).

### 2.2 Spatial Transcriptomics Profiling and Brain Region Identification

**Figure 2:**
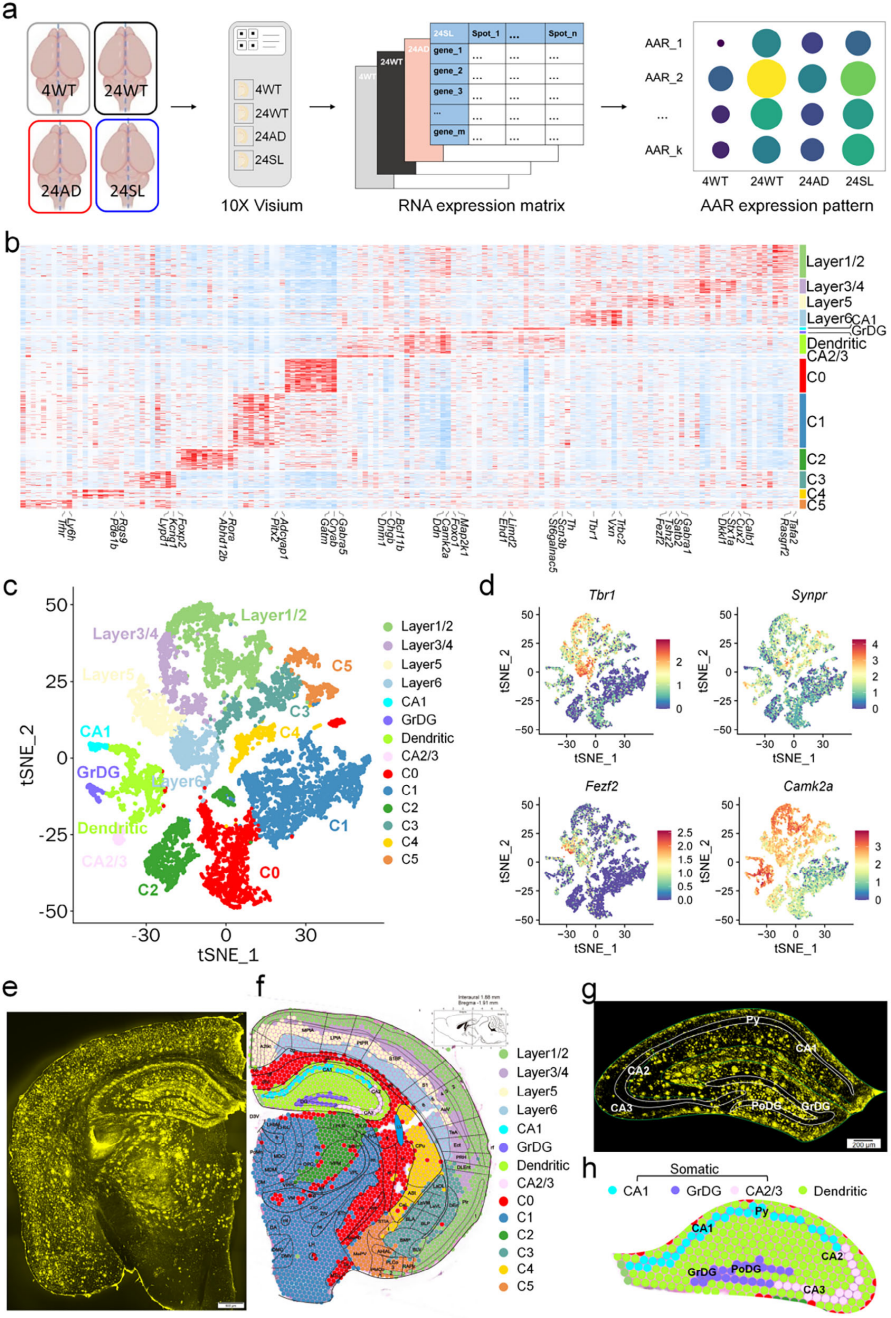
Spatial profiling of spatial transcriptomics in AD mice at late stages. a) Flow diagrams of spatial transcriptomics for 4WT (wildtype mice at 4‐month‐old), 24WT (wildtype mice at 24‐month‐old), 24AD (AD mice at 24‐month‐old), and 24SL mice (AD mice treated with SL). AD mice, APP/PS1 transgenic mice. AAR, following aging, AD, and rescue expression pattern. All mice used here were female. b) Identified marker genes for 14 brain regions. c) The 14 brain regions visualized by using the t‐distributed stochastic neighbour embedding (tSNE) were distinguishable from each other. d) The representative brain region‐specific genes, *Tbr1* (T‐box brain transcription factor 1, a specific biomarker of layer VI neurons in the neocortex)*, Synpr* (Synaptoporin, a presynaptic vesicle protein specifically enriched in mossy fiber terminals of the hippocampus), *Fezf2* (FEZ family zinc finger 2, a critical transcription factor for the specification of subcerebral projection neurons and a definitive biomarker of layer V neurons in the neocortex), and *Camk2a* (Calcium/calmodulin‐dependent protein kinase II alpha, a pan‐biomarker for excitatory neurons throughout the forebrain) were also separated from each other by tSNE. e) Aβ immunofluorescent staining for a half of coronal brain slice indicated widespread Aβ plaques in AD mice at 24‐month‐old. f) Spatial transcriptomics for another half of coronal brain slice from AD mice at 24‐month‐old indicated 14 brain regions nicely matching to the Paxinos and Franklin's the Mouse Brain in Stereotaxic Coordinates (4th Edition). g) Amplification of the hippocampus for Aβ immunofluorescent staining. Almost no Aβ plaques in the somatic region (the cell layer of CA1, CA2/3, and GrDG) but dense Aβ plaques in the dendritic region (green). h) Amplification of the hippocampus for the brain regions derived from spatial transcriptomics.

SL would have produced multiple mechanisms of action on the hippocampus in an aging‐dependent manner. Recent progresses suggest that spatial transcriptomics is a novel tool for studying AD mechanisms with spatial resolution in situ,^[^ ^23^, ^24^ ^]^ we thus used this tool to explore aging‐dependent mechanisms in AD mice with SL treatment. For this purpose, spatial transcriptomics was conducted on the brain slices, in an experimental design considered aging, AD, and rescue effect simultaneously: WT mice at 4‐month‐old (4WT), 24‐month‐old (24WT), and AD mice at 24‐month‐old (24AD), fed with normal pellets; AD mice at 24‐month‐old fed with normal pellets mixed with SL estimated 20 mg kg day^−1^ from 14‐ to 24‐month‐old (24SL) (**Figure** **2**a). Experimental data analysis showed that spatial transcriptomics data were in high quality (see Figure S3, Supporting Information). The spatially variable genes (SVG)^[^ ^25^ ^]^ were used to identify 14 brain regions by unsupervised clustering (Figure 2b). These 14 brain regions included the cortical 1/2, 3/4, 5 and 6 layers, the CA1 and CA2/3 cellular layers, the dentate gyrus (GrDG) granular layer, and the remaining dendritic region of the hippocampus; the other clusters (C0‐5), each covering multiple brain regions. Furthermore, these brain regions were easily distinguished from each other in the t‐distributed stochastic neighbour embedding (tSNE) visualization (Figure 2c). Also, this clustering agreed with the established cellular markers for the regions (see Figure 2c,d and Figure S4a, Supporting Information). Representative examples include *Tbr1* **(T‐box brain transcription factor 1, a specific biomarker of layer VI neurons in the neocortex)**, *Synpr* (Synaptoporin, a presynaptic vesicle protein specifically enriched in mossy fiber terminals of the hippocampus), *Fezf2* (FEZ family zinc finger 2, a critical transcription factor for the specification of subcerebral projection neurons and a definitive biomarker of layer V neurons in the neocortex), and *Camk2a* (Calcium/calmodulin‐dependent protein kinase II alpha, a pan‐biomarker for excitatory neurons throughout the forebrain), as visualized in Figure 2d. The other 16 genes investigated (see Figure S4b, Supporting Information) were also classic brain region biomarkers (detailed in the figure legend). Notably, we observed a stark regional disparity in Aβ deposition. While the C1 region (which almost overlaps with the hypothalamus) and the cellular layers of the CA1, CA2/3, and GrDG areas deposited almost no Aβ plaques, widespread deposition was evident in the other 10 brain regions examined in 24‐month‐old AD mice (Figure 2e,f; for region abbreviations, see Table S2, Supporting Information). Given that the hippocampus is a core brain region for learning and memory, we performed a further subdivision within it based on the striking contrast in Aβ deposition: the cellular layers (CA1, CA2/3, GrDG) were highly resistant to the plaques, while the surrounding regions were highly susceptible. Thus, the cellular layers of the CA1, CA2/3, and GrDG of the hippocampus was termed as somatic region (distributed dense cell bodies) and the remaining region within the hippocampus was termed as dendritic one (distributed sparse cell bodies but dense dendrites) (Figure 2g,h).

### 2.3 An AAR Rule Covered ATP Generation and Glut1 Expression

**Figure 3:**
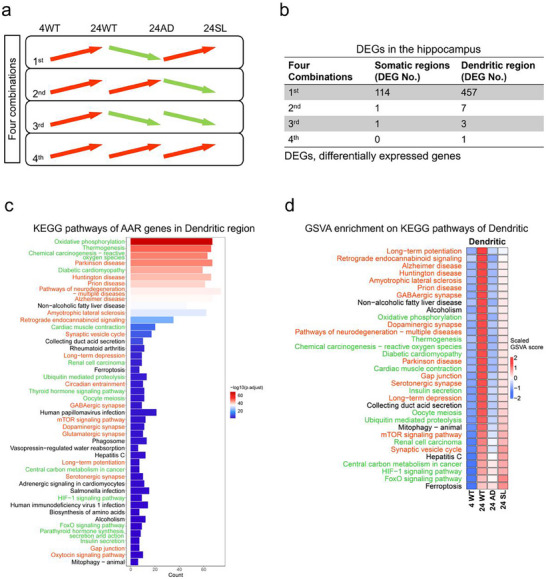
AAR rule and oxidative phosphorylation in the hippocampus. a) The hippocampus contained differentially expressed genes (DEGs) in four combinations of expression patterns, in which the type 1 was a rule of expression, defined as aging‐AD‐rescue (AAR), followed by increased during aging (4WT vs 24 WT), decreased during aging with AD (24WT vs 24AD) but rescued by SL treatment (24AD vs 24SL). b) AAR rule (type 1) was stronger in dendritic region (457 DEGs) than in somatic regions (109 DEGs) of the hippocampus. Other expression combinations (types 2, 3, and 4) covered few DEGs. c) The Kyoto Encyclopaedia of Genes and Genomes (KEGG) analysis on DEGs with AAR expression rule showing three classes of enriched signalling pathways in dendritic region of the hippocampus, in which oxidative phosphorylation was ranked No. 1. d) The gene set variation analysis (GSVA) indicating the AAR rule covered these signalling pathways in dendritic region, in which long‐term potentiation was ranked No.1.

Spatial transcriptomics data over 21000 genes was then analysed to search for differentially expressed genes (DEGs) reached statistically significant level in all of the three pairwise comparisons: 4WT vs 24WT, 24WT vs 24AD, and 24AD vs 24SL, representing these DEGs satisfying aging, AD, and rescue by SL, simultaneously. Such a rigorous selection identified 12002 DEGs distributed in all possible expression combinations (2 × 2 × 2 = 8) from 14 brain regions. It is very interesting that 4WT vs 24WT comparison led to 11166 DEGs with increased expression during aging but only 100 DEGs with decreased expression, implying that some adaptive mechanisms are activated in WT mice during healthy aging. Furthermore, 24WT versus 24AD comparison resulted in 7692 DEGs with decreased expression except 524 DEGs with increased expression, suggesting that these adaptive mechanisms are likely lost in AD mice with aging. Finally, the comparison 24AD versus 24SL output 4267 DEGs with increased expression but 633 DEGs with decreased expression, indicating that these adaptive mechanisms are possibly repaired in AD mice with aging after SL treatment. Based on these coordinated shifts, we identified a core set of genes that exhibited a consistent three‐stage expression pattern: (1) upregulated during healthy aging (Aging, 4WT vs 24WT), (2) downregulated in Alzheimer's disease (AD, 24WT vs 24AD), and (3) rescued upon SL treatment (Rescue, 24AD vs 24SL). This pattern is highly consistent with our AAR hypothesis. The AAR pattern is not merely a descriptive label; it provides a powerful functional filter to pinpoint genes whose expression is intrinsically linked to both the progression of pathology and the therapeutic mechanism of SL. Genes following the AAR rule are strong candidates for mediating the adaptive mechanisms of healthy aging that are lost in AD and subsequently restored by treatment. This framework allows us to move beyond differential expression and focus on a functionally coherent set of genes that likely play critical roles in AD, offering new insights into both disease mechanism and therapeutic action. The other 7 combinations just covered few DEGs. Notably, there were 584 DEGs in the hippocampus with only 4 combinations (**Figure** **3**a), in which the dominant combination type involved DEGs were further separated into 457 and 114 DEGs in dendritic and somatic region, respectively, suggesting a stronger AAR rule in dendritic than in the somatic region of the hippocampus (Figure 3b). Because SL reversed spatial memory defects in AD mice at 16‐month‐old, these DEGs within the hippocampus would be associated with the mechanisms of action produced by SL treatment.

Based on these dendritic DEGs (457) in the hippocampus, Kyoto Encyclopaedia of Genes and Genomes (KEGG) enrichment and gene set variation analysis (GSVA) identified KEGG and GSVA pathways, respectively. The KEGG pathways were mainly classified into three‐modules: metabolisms, neural functions, and the others, in which oxidative phosphorylation was ranked No.1 (Figure 3c). The GSVA pathways were also classified into similar modules showing an AAR rule, and the pathways were sorted by ranking scores of 24WT as possible protection mechanisms during aging, in which synaptic long‐term potentiation (LTP), the major cellular mechanism of learning and memory,^[^ ^26^ ^]^ was ranked No.1 (Figure 3d). The somatic DEGs (114) in the hippocampus showed KEGG pathways similar to those of the dendritic DEGs in the hippocampus, while oxidative phosphorylation was ranked No.1 in both KEGG and GSVA pathways (see Figure S5a,b, Supporting Information).

**Figure 4:**
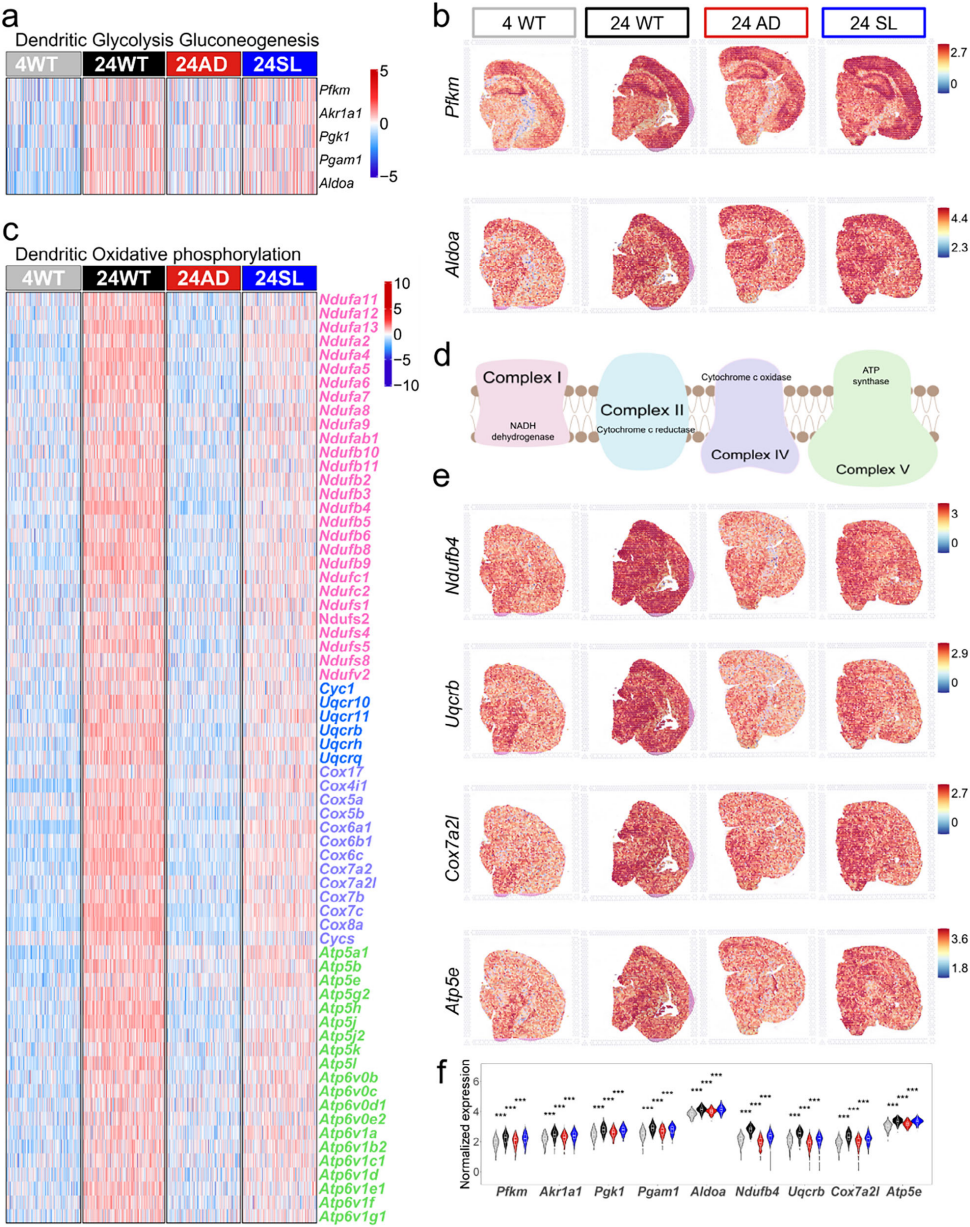
Glucose metabolism with AAR rule in hippocampal dendritic region. a) Heatmap showing relative RNA abundance with AAR rule among 4 samples that covered 5 DEGs in glycolysis gluconeogenesis. The row and column indicate the gene and spot respectively. The color bar represents the normalized gene expression level. b) Representative DEGs (*Pfkm* and *Aldoa*) showing as relatively spatial RNA abundance with AAR rule among mouse slices. c) Heatmap showing relative RNA abundance with AAR rule among 4 samples that covered four‐classes of gene family (67 DEGs) of oxidative phosphorylation at mitochondrial respiratory chain complexes (MRCC I, II, IV, and V). The heatmap indicates the same representation as that in panel A. d) Schematic graph for MRCC I, II, IV, and V. e) The representative DEGs in four‐classes of gene family (*Ndufb4, Uqcrb, Cox7a2l*, and *Atp5e*) showing as relatively spatial RNA abundance with AAR rule among 4 samples. f) Violin plots for the representative DEGs with AAR rule in hippocampal dendritic region. Gray, 4WT; black, 24WT; red, 24AD; blue, 24SL. Statistical analysis was performed using the Wilcoxon rank‐sum test. The sample sizes for each group in Dendritic were 4WT (*n* = 214), 24WT (*n* = 235), 24AD (*n* = 212), and 24SL (*n* = 213). ^*^ *p *< 0.05, ^**^ 0.01 < *p *< 0.05, ^***^ *p* < 0.001. Data are presented with the box indicating the interquartile range and the line representing the median.

Given that SL was synthesized from ferulic acid and enanthic acid (or heptanoic acid), and the latter one is known for supporting oxidative phosphorylation and ATP generation, and oxidative phosphorylation was highly enriched in both KEGG and GSVA analyses, we further focused on the key genes contributing to ATP production within these processes. ATP biosynthesis is driven by three tightly coupled biochemical stages: 1) glycolysis gluconeogenesis (the cytosolic breakdown of glucose to pyruvate, yielding a small amount of ATP); 2) the tricarboxylic acid (TCA) cycle (which oxidizes acetyl‐CoA to generate reducing equivalents NADH and FADH_2_); and 3) oxidative phosphorylation (wherein the electron transport chain utilizes these reducing equivalents to establish a proton gradient across the inner mitochondrial membrane that drives ATP synthase to produce the majority of ATP). We therefore systematically examined the AAR‐based expression patterns of DEGs associated with these three core bioenergetic pathways. Five DEGs with AAR rule were enriched in the glycolysis gluconeogenesis pathway (**Figure** **4**a). The representatives *Pfkm* and *Aldoa* expression showing as relatively spatial RNA abundance in the brain slices of 4WT, 24WT, 24AD, and 24SL actually followed an AAR rule (Figure 4b). Notably, we found 67 DEGs covering four gene families (Figure 4c) that were enriched in the mitochondrial respiratory chain complexes I, II, IV, and V, which are well‐known critical for oxidative phosphorylation (Figure 4d). The representatives *Ndufb4*, *Uqcrb*, *Cox7a2l*, and *Atp5e* expression for these four gene families showing as relatively spatial RNA abundance in the brain slices also followed an AAR rule (Figure 4e). The 5 DEGs in the glycolysis gluconeogenesis and the representatives 4 DEGs in the oxidative phosphorylation were violin‐plotted to show a typical AAR rule (Figure 4f). However, the tricarboxylic acid cycle pathway was not reported by enrichment analyses, but 4 genes, *Aco2*, *Idh3g*, *Idh3b*, and *Mdh1*, in this pathway were identified to follow an AAR rule. This study suggested that the DEGs with AAR rule covered ATP generation in the dendritic region of the hippocampus.

**Figure 5:**
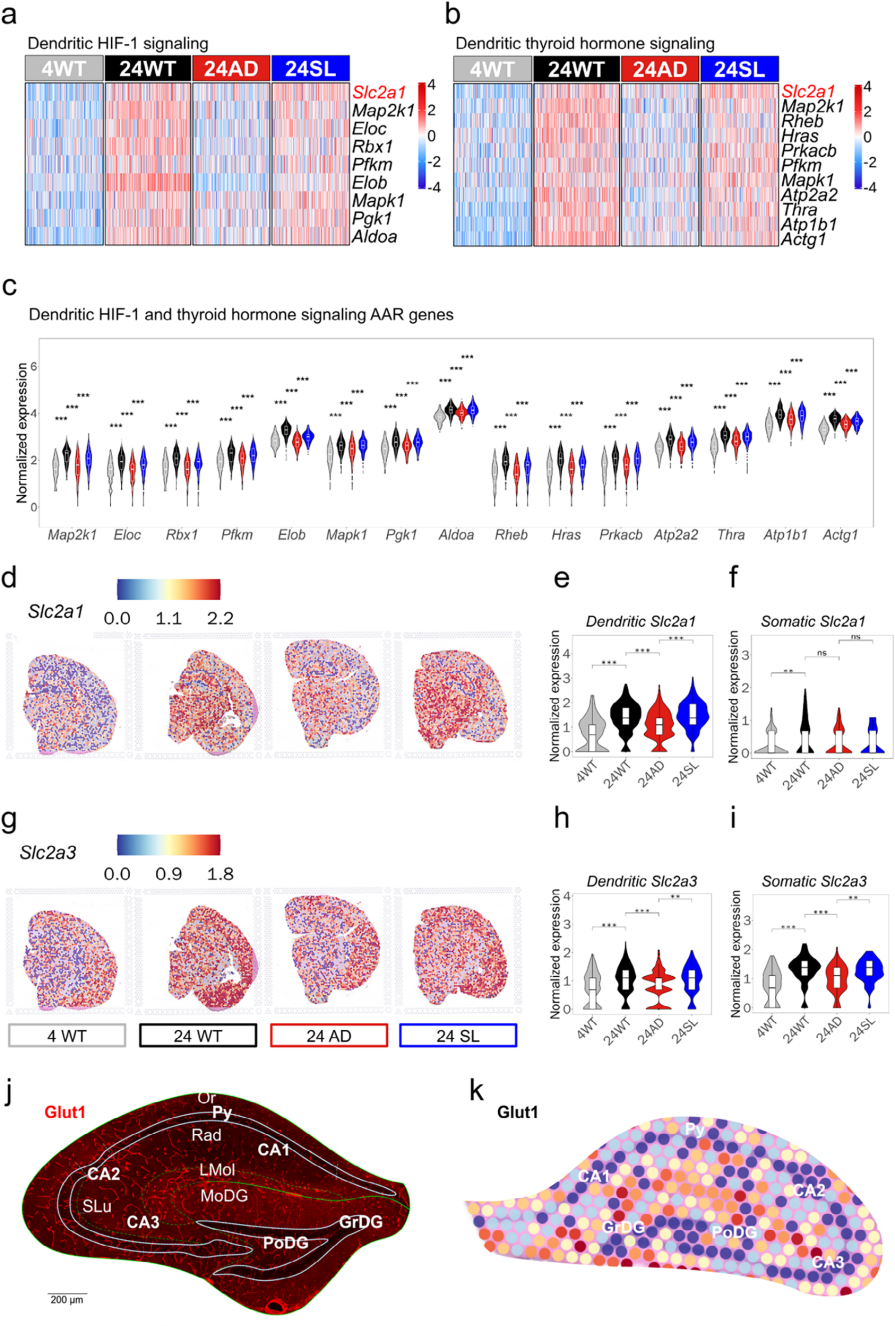
AAR rule covered Glut1/3 expression and their upstream signaling pathways in hippocampal dendritic region. a,b) Heatmap showing relative RNA abundance with AAR rule among 4 samples covered *Slc2a1* (coded Glut1) in both HIF‐1 and thyroid hormone pathways. c) Violin plot showing DEGs in HIF‐1 and thyroid hormone pathways to regulate Glut1 expression with AAR rule. Statistical analysis was performed using the Wilcoxon rank‐sum test. The sample sizes for each group in Dendritic were 4WT (*n* = 214), 24WT (*n* = 235), 24AD (*n* = 212), and 24SL (*n* = 213). ^*^ *p *< 0.05, ^**^ 0.01 < *p *< 0.05, ^***^ *p* < 0.001. Data are presented with the box indicating the interquartile range and the line representing the median. d–f) *Slc2a1* showed relatively spatial RNA abundance in slices with AAR rule, which was stronger in dendritic than in somatic region of the hippocampus. Statistical analysis was performed using the Wilcoxon rank‐sum test. The sample sizes for each group in Dendritic and Somatic were 4WT (*n* = 214), 24WT (*n* = 235), 24AD (*n* = 212), 24SL (*n* = 213) and 4WT (*n* = 83), 24WT (*n* = 105), 24AD (*n* = 75), 24SL (*n* = 84) respectively.^*^ *p *< 0.05, ^**^ 0.01 < *p* < 0.05, ^***^ *p* < 0.001. Data are presented with the box indicating the interquartile range and the line representing the median. g–i) *Slc2a3* (coded Glut3) showing as relatively spatial RNA abundance in slices with AAR rule, which was similar in dendritic and somatic region of the hippocampus. Statistical analysis was performed using the Wilcoxon rank‐sum test. The sample sizes for each group in Dendritic and Somatic were 4WT (*n* = 214), 24WT (*n* = 235), 24AD (*n* = 212), 24SL (*n* = 213) and 4WT (*n* = 83), 24WT (*n* = 105), 24AD (*n* = 75), 24SL (*n* = 84) respectively.^*^ *p *< 0.05, ^**^ 0.01 < *p* < 0.05, ^***^ *p* < 0.001. Data are presented with the box indicating the interquartile range and the line representing the median. j) Immunofluorescent staining of Glut1 (red) in WT mice at 24‐month‐old showing that Glut1 was mainly expressed in microvessels that were distributed denser in dendritic than in somatic region of the hippocampus. k) Color‐coded relative levels of Glut1 expression in each spot detected by spatial transcriptomics in the hippocampus of 24WT showing high and low levels of Glut1 expression in dendritic and somatic region of the hippocampus, respectively.

Notably, further analyses tracked dendritic DEGs of the hippocampus down onto hypoxia‐inducible factor‐1 (HIF‐1) and thyroid hormone pathways, both of which engaged a gene *Slc2a1*, coding Glut1 (**Figure** **5**a,b). Furthermore, violin plot confirmed that the dendritic DEGs covering HIF‐1 and thyroid hormone pathways followed an AAR rule (Figure 5c). This is particularly interesting because Glut1 expression would have well explained a possible‐adaptive mechanism by enhancing its expression in WT mice during healthy aging (4WT vs 24 WT). In contrast, this mechanism was lost in AD mice with aging (24WT vs 24AD) but that was rescued in AD mice with aging by SL treatment (24AD vs 24SL).

It is also very interesting that molecular docking analysis based on SL's structure and structure‐known proteins of the dendritic DEGs resulted in MAPK1 and HRAS of these two pathways, with a high affinity binding with SL (‐6.5 and ‐5.9 Kcal/mol) (see Figure S6a,b, Supporting Information), implying that SL may regulate Glut1 expression through a direct action on MAPK1 and HRAS. Another protein ADD1 with a high affinity binding with SL (‐5.2 Kcal/mol) was also reported by docking experiment (see Figure S6c, Supporting Information). ADD1 expression dependent on aging is previously reported^[^ ^27^ ^]^ and may play an important role in both AD and aging.^[^ ^28^ ^]^ We further performed an in vitro binding assay using a human proteome microarray to experimentally validate these direct interactions (see Figure S6d, Supporting Information).

Then, we analysed *Slc2a1* (coding Glut1) expression showing as relatively spatial RNA abundance in the brain slices of 4WT, 24WT, 24AD, and 24SL, suggesting that Glut1 expression was covered by an AAR rule (Figure 5d), which was also stronger in dendritic (Figure 5e) than in somatic region of the hippocampus as shown in violin plots (Figure 5f). Furthermore, another insulin‐insensitive *Slc2a3* (coding Glut3) expression shown as relatively spatial RNA abundance in the brain slices of the groups also followed AAR rule (Figure 5g) in both dendritic and somatic region of the hippocampus (Figure 5h,i). Moreover, both immunofluorescent staining of Glut1 and spatial transcriptomics spot of relative Glut1 expression showed that Glut1 expression (Figure 5j, red) was indeed stronger in the dendritic (warmer colour) than in somatic region (cooler colour) of the hippocampus (Figure 5k). This pattern is likely attributable to the distribution of microvessels, i.e. the small blood vessels (≤ 20 µm in diameter) that highly express Glut1 as a key glucose transporter on endothelial cells. The higher density or activity of these microvessels in dendritic region may locally increase Glut1‐derived glucose availability, supporting the enhanced metabolic demands of synaptic and dendritic compartments. Taking together, this set of spatial transcriptomics experiments indicated that glucose metabolism (or ATP generation) and Glut1 expression (glucose supply) were the major mechanisms of action produced by SL treatment, particularly in the dendritic region of the hippocampus.

### 2.4 SL Treatment Improves Hippocampal Glut1 Expression and ATP Generation

**Figure 6:**
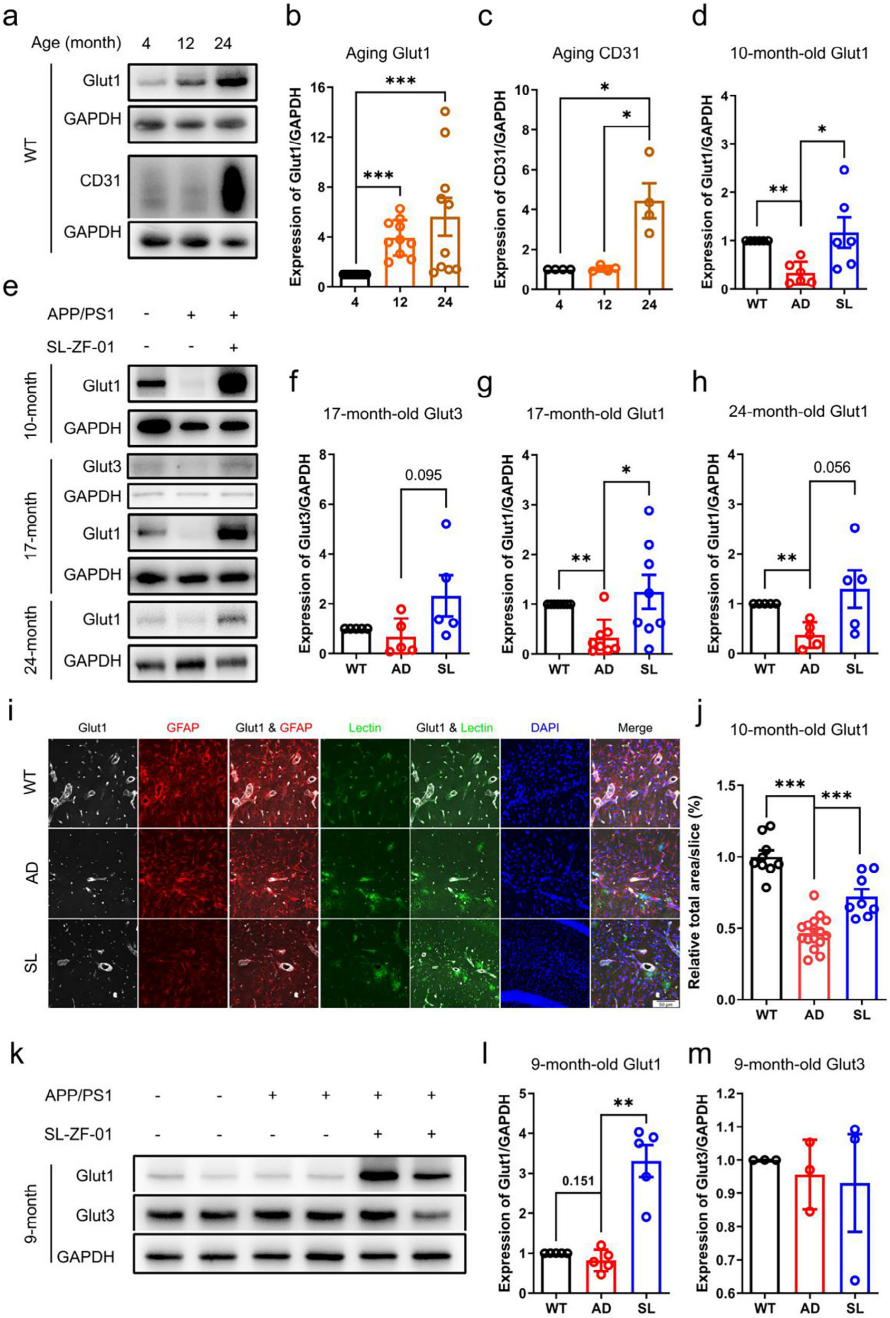
SL increased Glut1 and Glut3 expression in the hippocampus. a–c) Immunoblot analysis of hippocampal Glut1 in wild‐type (WT) mice at 4, 12, and 24 months of age indicated an overall increase in expression with advancing age. (b) Pairwise comparisons using the Mann‐Whitney test revealed a significant increase between 4 and 12 months (*p* < 0.0001, *n* = 10) and between 4 and 24 months (*p* < 0.0001, *n* = 10), whereas no significant change was observed between 12 and 24 months (*p* = 0.8534, *n* = 10). (c) The expression of CD31 (platelet endothelial cell adhesion molecule 1) was significantly elevated only at 24 months compared to both 4 and 12 months (*p* = 0.028 for each comparison, *n* = 4 per group). d‐h. Immunoblotting of hippocampal Glut1 of the mice at 10, 17, and 24‐month‐old suggested decreased expression during aging with AD but that was rescued by SL relative to WT mice. Hippocampal Glut3 expression showed a numerical decrease in AD mice at 17‐month‐old but that appeared to be rescued by SL. (d) At 10 months of age, hippocampal Glut1 expression was significantly decreased in AD mice compared to WT controls (*p* = 0.002, *n* = 6), and this decrease was significantly rescued by SL treatment (AD vs. SL, *p* = 0.015, *n* = 6). f) At 17 months, no significant difference in hippocampal Glut3 expression was observed between AD and WT mice (*p* = 0.690, *n* = 5). Similarly, the difference between AD and SL‐treated groups did not reach statistical significance (*p* = 0.095, *n* = 5). g) Consistent with the 10‐month time point, Glut1 expression at 17 months was again significantly lower in AD mice than in WT (*p* = 0.0057, *n* = 8), and SL treatment significantly restored its expression (AD vs. SL, *p* = 0.0148, *n* = 8). h) By 24 months of age, a significant reduction in hippocampal Glut1 persisted in AD mice relative to WT (*p* = 0.0079, *n* = 5). The difference between AD and SL‐treated groups at this stage was not statistically significant (*p* = 0.0556, *n* = 5). i,j) Immunofluorescent staining of Glut1, Glial Fibrillary Acidic Protein (GFAP), lectin, and 4’6‐diamidino‐2‐phenylindole (DAPI) in the hippocampus for WT, AD, and SL mice at 10‐month‐old suggested that the hippocampal neurovascular units, the microvessels (lectin, green), astrocytes (GFAP, red), and neurons (DAPI, blue) were decreased and distorted in AD mice relative to WT mice but these were largely rescued by SL treatment. j) Quantification of the relative total area per slice confirmed a significant reduction in AD mice relative to WT controls (*p* < 0.0001; WT *n*=9, AD *n*=16). This decrease was significantly rescued by SL treatment, as evidenced by a substantial increase in the measured area compared to the AD group (AD vs. SL, *p* = 0.0001; SL *n*=8), based on Dunnett's multiple comparisons test. k–m) Glut1 and Glut3 expression in AD mice at 9‐month‐old remained nearly unchanged relative to WT mice at the same age. SL treatment increased Glut1 but not Glut3 expression in the hippocampus. (l) For Glut1, no significant difference was observed between WT and AD mice (*p* = 0.151, *n* = 5). However, SL treatment resulted in a significant increase in Glut1 expression compared to the AD group (*p* = 0.0079, *n* = 5). (m) In contrast, Glut3 expression levels showed no significant difference between WT and AD mice, nor between AD and SL‐treated groups (*p* = 0.7000, *n* = 3). ^*^ *p* < 0.05; ^**^ *p* < 0.01; ^***^ *p* < 0.001 by Mann‐Whitney test or one‐way ANOVA with Tukey's multiple comparisons test. Data are presented as mean ± SEM. All mice used here were female.

This set of experiments was performed for confirming the above findings. As shown by immunoblotting study on the hippocampus of the WT mice at 4, 12, and 24‐month‐old, hippocampal Glut1 expression was increased during healthy aging (**Figure** **6**a,b). The hippocampal blood vessel marker CD31, also known as the platelet endothelial cell adhesion molecule 1 (PECAM‐1), was also increased expression in the WT mice at 24‐month‐old (Figure 6a,c). Furthermore, immunoblotting study suggested that hippocampal Glut1 expression was decreased in the AD mice at 10, 17, and 24‐month‐old, relative to the WT mice at the same ages, but it was consistently reversed in AD mice at these stages by SL treatment (Figure 6d,e,g,h). Hippocampal Glut3 expression was decreased on average in AD mice, but this reduction appeared rescued by SL treatment (*p* = 0.095), relative to the WT mice at the same age (Figure 6e,f). Moreover, immunofluorescent study in the AD mice at 10‐month‐old suggested that hippocampal Glut1 expression was mainly located in the microvessels as indicated by lectin signals (green in Figure 6i) and also in the astrocytes as indicated by glial fibrillary acidic protein (GFAP) signals (red in Figure 6i). Notably, hippocampal Glut1 expression measured as signal area was decreased in the AD mice relative to the WT mice but rescued in the AD mice after SL treatment for two months (Figure 6j). By contrast, hippocampal Glut1 expression was not decreased in the AD mice at 9‐month‐old relative to the WT mice (*p* = 0.151) but it was still increased by SL treatment for two months (*p* < 0.01) (Figure 6k,l), while hippocampal Glut3 expression was not different among WT, AD, and SL groups (Figure 6k,m). This implies that SL treatment has little effects on spatial memory defects in the AD mice at 9‐month‐old may attribute to no changes of the hippocampal Glut1/3 expression at this early stage.

**Figure 7:**
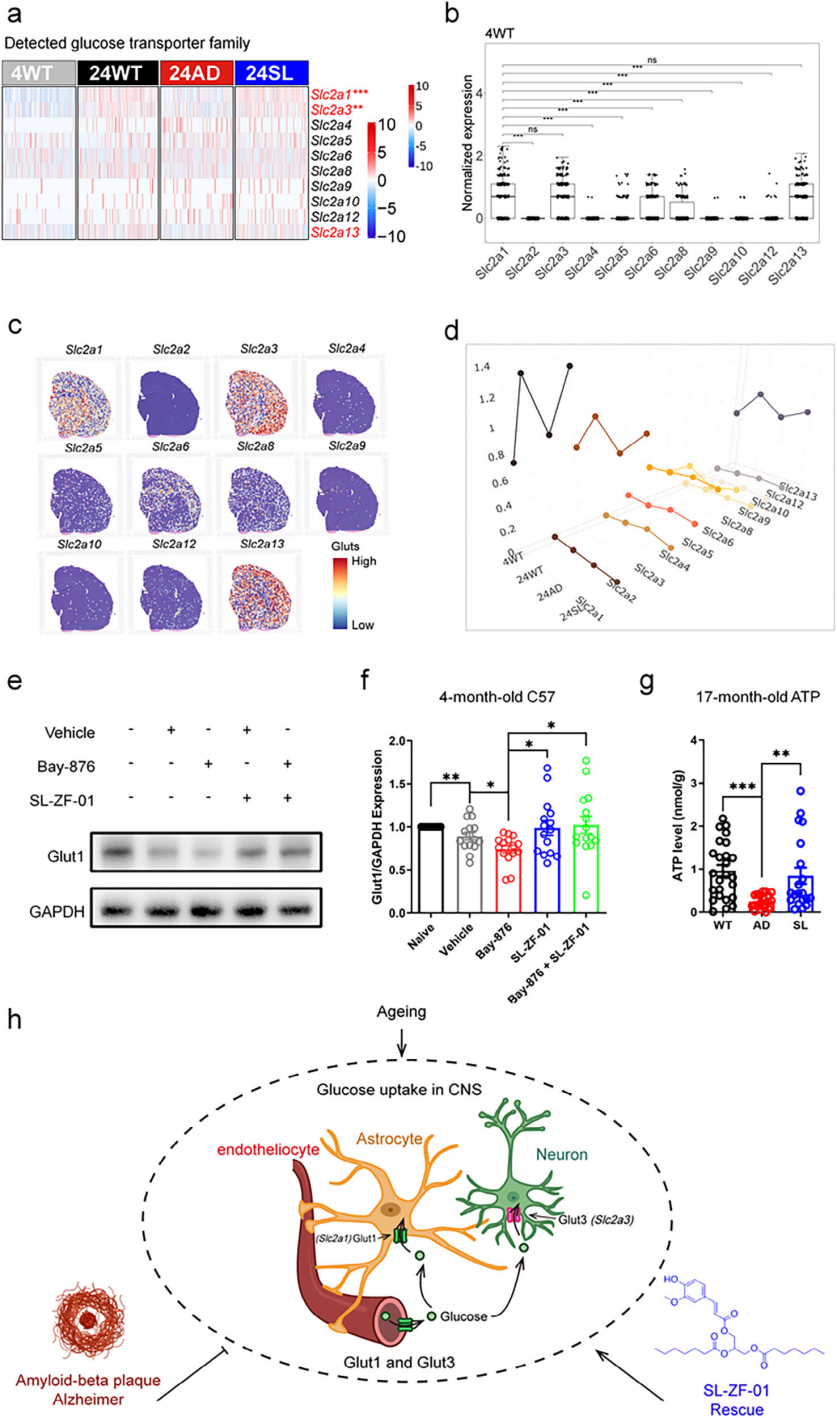
Glut family and ATP generation responding to SL treatment. a) Heatmap showing relative RNA abundance of 10 members of the glucose transporters family. b) *Slc2a1*, *Slc2a3*, and *Slc2a13*, coded Glut 1, 2, and 3, were expressed significantly higher RNA levels than other Glut members in WT mice at 4‐month‐old (4WT). Statistical analysis was performed using the Wilcoxon rank‐sum test. The sample sizes of 4WT in all region is 2354. ^*^ *p *< 0.05, ^**^ 0.01 < *p *< 0.05, ^***^ *p* < 0.001. Data are presented with the box indicating the interquartile range and the line representing the median. c) Relatively spatial RNA abundance for Glut family showing that Glut 1, 3, and 13 were expressed higher levels than the other Glut members. d) Relative RNA abundance among 4 samples showing Glut1, Glut3, and Glut13 with an AAR expression rule in 3D graph (The x‐axis, y‐axis, and z‐axis indicated genes, 4 samples, and relative RNA abundance, respectively). e,f) The Glut1 inhibitor Bay‐876 inhibited hippocampal Glut1 expression relative to vehicle or naïve mice. SL alone or SL with Bay‐876 increased hippocampal Glut1 expression relative to Bay‐876 alone, suggesting that SL produced an agonist‐like action to counteract Bay‐876's inhibition on hippocampal Glut1 expression. g) Hippocampal ATP levels were largely decreased in AD mice at 17‐month‐old but rescued by SL treatment relative to WT mice at the same age. All mice used here were male. h) The glucose transporter (Glut) type 1 (Glut1, green) is mainly expressed in the microvessels (< 20 µm) and astrocytes, but the Glut type 3 (Glut3, red) is mainly expressed in the neurons. These Glut1/3 constitute a constant supply of glucose crossing blood brain barrier to the brain. We proposed a hypothesis that the pathologies of Alzheimer's disease (AD) might vary following aging‐dependent different stages (or disease progression). Consistent with this point of view, Glut1/3 expressions were increased during healthy aging but decreased during aging with AD, in which these lost protection mechanisms were rescued by SL‐ZF‐01 (SL) treatment. ^*^ *p* < 0.05; ^**^ *p* < 0.01; ^***^ *p* < 0.001 by Mann‐Whitney test or one‐way ANOVA with Tukey's multiple comparisons test. Data are presented as mean ± SEM.

Finally, we further analysed the glucose transporter (Glut) family detected by spatial transcriptomics on the brain slices of 4WT, 24WT, 24AD, and 24SL (**Figure** **7**a). The insulin‐unresponsive Glut1, Glut3, and Glut13 were expressed in high levels, while Glut5, Glut6, and Glut8 were expressed in middle levels, but the remaining ones including insulin‐responsive Glut4 were expressed in low levels (Figure 7b,c). Glut1, Glut3, and Glut13 expression indeed followed an AAR rule (Figure 7d). Notably, Bay‐876, a known Glut1 inhibitor, suppressed hippocampal Glut1 expression that was increased by SL treatment, suggesting opposite effects on hippocampal Glut1 expression produced by these active agents (fed for one month). The effect of Bay‐876 on hippocampal Glut1 expression seemed to be antagonized by SL, leading to an expression level of hippocampal Glut1 similar to that of SL alone (Figure 7e,f). Most important of all, hippocampal ATP levels were largely decreased in the AD mice relative to the WT mice at 17‐month‐old but they were nicely rescued in the AD mice after SL treatment for two months (Figure 7g), indicating that ATP generation also follows an AAR rule.

This set of experiments suggested that hippocampal Glut1 expression was indeed increased during healthy aging, but it was not decreased in younger AD mice (at 9‐month‐old) until 10‐month‐old or older (at 17‐ and 24‐month‐old). SL treatment consistently increased hippocampal Glut1 expression in the AD mice at 9‐month‐old and older (10‐, 17‐, and 24‐month‐old). Glut1, Glut3, and Glut13 expression but not the other Glut members followed an AAR rule. Besides, SL may serve as a positive Glut1 modulator to counteract the effect of the Glut1 inhibitor Bay‐876 on inhibiting Glut1 expression. SL can also rescue hippocampal ATP generation. In short, SL reversed memory defects in the AD mice at late stages by targeting or modifying multiple mechanisms of action on glucose supply and ATP generation.

### 2.5 AAR Genes as a Shared Resource and their Integrated Network in AD and Aging

We provided a comprehensive list of 3,065 AAR genes (Table S4, Supporting Information) across all regions, as well as a curated set of 101 unique AAR genes (Table S5, Supporting Information) ranked within the top 30 based on ANOVA‐derived p‐values, to facilitate further reference by researchers. With the aim of how the AAR genes might contribute to aging and AD pathology, we constructed a PPI network using the STRING database (https://string‐db.org/) integrating top 200 AAR genes with 383 AD‐related genes (https://www.genome.jp/kegg/) and 136 aging‐related genes (https://genomics.senescence.info/genes/models.html), revealing close interaction patterns between AAR genes and both AD‐ and aging‐associated genes (Figure S8a, Supporting Information). Furthermore, we conducted KEGG and GO enrichment analyses on the 3,065 AAR genes to investigate the molecular functions potentially engaged during the process in which SL appears to restore the AD mouse brain toward a state of healthy aging. These analyses indicated that in addition to glucose metabolism and ATP metabolism described above, endocytosis/exocytosis, neuronal apoptosis, and autophagy were also significantly enriched by AAR genes (Figure S8b,c, Supporting Information). Collectively, these findings suggest that AAR genes may play a crucial role in the progression of both aging and AD, and that the therapeutic effect of SL may depend on orchestrating a comprehensive and complex functional network to counteract AD pathologies.

## 3 Discussion

The prevailing amyloid cascade hypothesis has guided Alzheimer's disease (AD) therapeutic development for decades. However, the limited clinical success of mono‐targeted Aβ therapies in late‐stage trials, coupled with growing scientific evidence, points to a more complex and stage‐dependent pathology. Our AAR hypothesis proposes a dynamic interplay between aging and AD, to lead to the trajectory of brain aging pathologically diverted. Normal aging involves a gradual decline in certain cellular functions but AD pathogenesis accelerates and exacerbates this decline, leading to catastrophic functional failure. Based on this assumption, an effective intervention should presumably not only halt the progression but also reverse the pathological trajectory, to a state resembling healthy aging.

### 3.1 The AAR Hypothesis: A Transcriptomic Framework Linking Aging, Pathology, and Rescue

For decades, the amyloid cascade hypothesis has been the central dogma driving Alzheimer's disease (AD) therapeutic development.^[^ ^2^, ^29^ ^]^ While foundational, the limited clinical success of mono‐targeted Aβ therapies in patients with advanced disease has exposed its critical limitation: an inability to account for the profound impact of aging, the single greatest risk factor for AD.^[^ ^4^, ^30^ ^]^ Our study introduces the Aging‐AD‐Rescue (AAR) hypothesis, which reframes AD not as a simple acceleration of aging, but as a pathological deviation from the normal aging trajectory. We propose that healthy aging engages a compensatory transcriptomic program to maintain function, a program that is specifically disrupted in AD, leading to catastrophic failure. Therefore, an ideal intervention, therefore, should not merely slow decline but actively *re‐tune* the system toward a healthier aging state. Our discovery of SL, a compound that reverses memory defects in late‐stage AD mice, and the subsequent identification of the AAR gene pattern through spatial transcriptomics, provides robust experimental support for this dynamic model. This work positions the failure of adaptive aging mechanisms, particularly in brain energy metabolism, as a key driver of late‐stage AD, offering a new paradigm for therapeutic development.

### 3.2 SL Reverses Late‐Stage Cognitive Deficits: The Reversibility of Advanced AD

A most striking finding is that SL treatment reverses well‐established spatial memory defects in aged APP/PS1 mice burdened with widespread Aβ plaques. This cognitive rescue occurred with only a modest reduction in plaque load, demonstrating that significant functional recovery is achievable without requiring extensive amyloid clearance. The clinical trial outcomes of anti‐Aβ monoclonal antibodies like Aducanumab and Lecanemab, while showing promise in early AD, have demonstrated limited to no efficacy in patients with moderate‐to‐severe disease.^[^ ^4^, ^30^, ^31^ ^]^ Our data provide a compelling explanation: the dominant pathology driving cognitive decline shifts with disease progression. The age‐dependent efficacy of SL, being effective in older (16‐month) but not significant in younger (9‐month) AD mice, directly mirrors this clinical reality and suggests that the therapeutic targets relevant in late‐stage AD are distinct from those in the prodromal phase.

### 3.3 Spatial Transcriptomics and the AAR Rule: Pinpointing a Spatially Resolved Metabolic Crisis

To move beyond phenomenological observation, we employed spatial transcriptomics, a powerful tool that has recently been used to map the topographic landscape of AD pathology and aging.^[^ ^32^ ^]^ Our innovative application was to overlay the dimensions of aging, disease, and therapeutic rescue. This uniquely allowed us to define the AAR rule, a transcriptomic signature of lost adaptation and its pharmacological restoration. While previous omics studies have catalogued changes in aging or AD separately, our triple‐comparison design specifically filters for genes implicated in this critical interaction. This approach proved vastly more efficient than traditional and candidate‐driven methods.

The power of this spatial‐AAR integration was further highlighted by the discovery that this gene program was most prominent in the hippocampal dendritic region, an area we found to be highly susceptible to Aβ deposition, in contrast to the resistant somatic layers. This precise spatial matching between the site of greatest molecular perturbation and the primary locus of pathology represents a significant advance. It explains why bulk tissue analyses have likely underestimated the regional specificity of metabolic failure in AD. Our data subsequently narrowed this complex AAR signature onto a core mechanism: the dysregulation of brain glucose metabolism. KEGG and GSVA enrichment analyses consistently highlighted oxidative phosphorylation as the top pathway, and upstream regulatory analysis tracked this deficit to a loss of Glut1 expression, governed by HIF‐1 and thyroid hormone signaling pathways that themselves follow the AAR rule.

These mechanisms of action produced by SL are likely not unique for treating AD but could be common for treating neurodegenerative disorders (see Figure S7, Supporting Information), suggesting that this multitarget SL modifying aging‐related mechanisms may be a potential therapy for these brain diseases by repairing constant glucose supply and rescuing ATP generation in the brain.

### 3.4 Glut1 and Bioenergetics: Therapeutic Target in Late‐Stage AD

The critical role of cerebral glucose hypometabolism in AD has long been recognized from PET imaging studies.^[^ ^33^ ^]^ The “brain energy rescue” hypothesis has been elegantly proposed by Cunnane et al., suggesting that supporting brain bioenergetics could be a viable therapeutic strategy for neurodegenerative diseases.^[^ ^15^ ^]^ Further, a prior study demonstrated that knocking down Glut1 in an AD mouse model exacerbates cognitive deficits and Aβ pathology,^[^ ^34^ ^]^ establishing that impairing glucose transport can worsen the disease. Our findings build upon but crucially extend these important observations. We demonstrate that the natural, age‐dependent upregulation of Glut1, a putative adaptive mechanism in healthy aging, is absent in AD, and that its restoration is well associated with cognitive recovery in late‐stage disease. This positions SL not as a symptomatic agent but as a DMT that addresses a fundamental, age‐associated pathophysiology.

Our work also intersects with growing interest in alternative metabolic interventions, such as ketogenic diets and medium‐chain triglycerides, which aim to bypass cerebral glycolytic deficits by providing ketone bodies as an alternative fuel source^.[^ ^35^, ^36^ ^]^ SL offers a complementary and potentially more direct strategy: instead of bypassing the compromised glucose metabolism machinery, it directly enhances the brain's intrinsic capacity for glucose utilization by boosting Glut1‐mediated transport and downstream ATP production. This is evidenced by the rescue of hippocampal ATP levels in SL‐treated AD mice to near‐normal levels.

Moreover, the binding targets of SL remained unclear, but our docking experiment suggested several possibilities: for example, SL might bind to MAPK1 and HRAS in the HIF‐1/thyroid hormone pathways for upregulating Glut1 expression, and bind on ADD1 for regulating synaptic LTP and memory formation. We performed an in vitro binding assay using a human proteome microarray to validate the direct interactions as shown in the Figure S6d (Supporting Information).

### 3.5 The Evolving Pathology of AD: Integrating Aβ and Metabolic Hypotheses

Our data support an integrated, stage‐dependent model of AD pathogenesis. We do not contest the importance of Aβ in initiating the disease cascade, particularly at early stages^.[^ ^2^, ^31^ ^]^ Instead, we propose that with advancing age and disease duration, the pathological epicenter shifts from Aβ‐centric toxicity to a broader crisis, such as brain energy metabolism. This view reconciles the success of Aβ‐clearing antibodies in early AD with their failure in late AD. It is also consistent with clinical data showing that the correlation between Aβ burden and cognitive impairment weakens as the disease progresses^,[^ ^37^, ^38^ ^]^ while the severity of mitochondrial dysfunction and hypometabolism becomes more pronounced.^[^ ^15^, ^39^ ^]^

In this model, early AD is driven by the toxic gain‐of‐function of Aβ and tau. However, over time, the chronic energy demand of combating these pathologies, combined with age‐related mitochondrial decline, pushes the brain's bioenergetic system past a tipping point. The failure of adaptive mechanisms like Glut1 upregulation then becomes the primary driver of neuronal dysfunction and cognitive collapse. This explains why a multi‐target agent like SL, designed to support oxidative phosphorylation and microvascular health, is effective at a stage when single‐target Aβ therapies are not. Our AAR gene list, which includes players in endocytosis, apoptosis, and autophagy beyond core metabolic pathways, suggests SL may orchestrate a broad restorative program. These AAR gene sets serve as a valuable resource for the scientific community, offering potentially new biomarkers and potential targets for future drug discovery.

### 3.6 Limitations

This study has several limitations. First, the investigation was limited to female mice, a choice driven by the more robust and consistent AD phenotype in aged female APP/PS1 models compared to males, which develop confounding comorbidities. Although the core mechanisms of SL‐ZF‐01 are not inherently sex‐specific, its efficacy in males warrants future validation. Second, while our data strongly associate cognitive rescue with enhanced Glut1 and bioenergetics, definitive causal proof is lacking. A planned rescue experiment using a Glut1 inhibitor (Bay‐876) was precluded by severe mortality in aged AD mice, an observation that itself underscores the critical dependence of the diseased brain on glucose transport. Future studies will employ genetic strategies, such as inducible Glut1 knockdown, to establish causality.

## 4 Conclusion

In conclusion, our study makes several significant contributions to the AD field. First, we propose the AAR hypothesis, a novel conceptual framework that explicitly links the biology of aging with AD pathogenesis and treatment response. Second, we report the unprecedented activity of SL, a compound that reverses memory defects in a late‐stage AD model, providing a much‐needed candidate for a disease stage with no effective DMTs. Third, we demonstrate the power of spatial transcriptomics coupled with the AAR analytical framework to efficiently deconstruct complex, multi‐target drug mechanisms in a spatially resolved manner. Finally, we provide evidence that Glut1‐dependent bioenergetic failure is a critical, necessary pathology in late‐stage AD. Therefore, our work provides strong evidence that the advanced AD is reversible. It paves a new therapeutic path by targeting the age‐related metabolic vulnerabilities that ultimately underlie cognitive demise, offering renewed hope for treating patients at advanced stages of this devastating disease.

## 5 Experimental Section

### Mice

APP/PS1 [B6. Cg‐Tag (APPswe, PSEN1dE9) 85Dbo/Mmjax] double transgenic mice, which is known as a model mouse of Alzheimer's disease (AD) that was thus termed AD mice, and their negative littermates (wildtype, WT) were purchased from the Model Animal Research Centre of Nanjing University, Nanjing, China or Cavens Biogle Model Animal Research Co., Ltd, Suzhou, China. The BKS‐db (BKS‐Leprem2Cd479/Gpt Strain NO.T002407) transgenic mice, which is known as a model mouse of diabetes (db/db), were purchased from GemPharmatech, Jiangsu, China.

All mice were maintained in animal facilities of Kunming Institute of Zoology, the Chinese Academy of Sciences, and fed ad libitum with standard laboratory chow (or pellet) and water in ventilated cages under a 12‐h light/dark cycle.

Perhaps due to health conditions of mice in the laboratory, females are more likely to survive during aging. Therefore, both male and female AD mice were used younger than 14‐month‐old but only female ones were used older than this age. Controls such as WT mice with the same gender were used. All mice used for spatial transcriptomics, immunoblotting, and immunofluorescent studies on the hippocampus were females. All db/db or C57BL/6J mice used or not mentioned elsewhere were male. All experiments followed the Principles for the Application Format for Ethical Approval for Research Involving Animals were approved in advance by the Institutional Animal Care and Use Committee, Kunming Institute of Zoology, the Chinese Academy of Sciences (SMKX‐2015019).

### Anesthesia for Sacrificing Animals

All mice were sacrificed by anesthesia with 4% Zoletil 50, 1 mg mL^−1^ Xylazine hydrochloride, and 0.5 µg mL^−1^ atropine sulfate monohydrate through subcutaneous injection at a dose of 1 mg/100g body weight. All mice were fast overnight before sacrifice.

### SL‐ZF‐01 and Experimental Designs

The ferul enanthate (E)‐3‐((3‐(4‐hydroxy‐3‐methoxyphenyl) acrylyl) oxy) propane‐1,2‐dienanthate (SL‐ZF‐01, SL) was synthesized by Shureli Biopharma Company (Kunming, China). Based on preliminary studies in AD mice at different stages and SL treatment, the present study reported the following experimental designs: 1. AD mice were fed with estimated 20 mg/kg/day SL diet (normal pellets + SL) or control diet (normal pellets), while all aged‐matched WT mice were fed with control diet, started at 14‐month‐old until 16/17‐month‐old or 24‐month‐old. The spatial learning task of the Morris water maze was carried out in the groups when the AD mice had been fed with SL (estimated 20 mg kg day^−1^) diet for two months (i.e., 16‐month‐old). 2. An independent group of AD mice was fed with estimated 20 mg kg day^−1^ SL diet and aged‐matched AD or WT mice were fed with control diet, starting at 8 or 9‐month‐old. The spatial learning task of the Morris water maze was performed at one month later. 3. To address the specific mechanisms of brain glucose metabolism in AD, db/db mice was utilized, a well‐established model of systemic insulin resistance characterized by impaired Glut4‐mediated glucose uptake. This model allows us to investigate whether the efficacy of SL depends on intact insulin signaling. WT mice served as normal controls to establish baseline cognitive performance. db/db mice were divided into two groups: one received an SL diet (estimated 20 mg/kg/day) and the other a control diet, while WT mice received the control diet. After one month of treatment, all animals underwent testing in the Morris Water Maze spatial learning task. 4. The C57BL/6J mice, slightly differed from the WT mice (negative littermates of AD mice) in genetic background, at 2‐month‐old were treated with estimated 20 mg kg day^−1^ SL or control diet for one month. These mice were then randomly divided into two groups, treated with the Glut1 inhibitor Bay‐876 (estimated 3 mg kg day^−1^ for one month) or vehicle through drinking water.

### Morris Water Maze

The water maze consists of a circular plastic tank with white color and 120 cm in diameter (Med Association, USA), filled with warm water (21–22 °C), the surface of which is covered by the polyethylene plastic tiny particles for obscuring a hidden platform (10 cm in diameter ≈ 5 cm below the surface) underwater. The experimental procedures used here are like those previously described.^[^ ^18^, ^22^ ^]^ Briefly, the spatial learning task of the Morris water maze includes acclimation, spatial learning training (4 trials/day for 6 days), and retrieval test (a probe trial at 24‐h after the final training trial). For acclimation, one trial was allowed for the animals freely swimming in the water maze when their swimming velocity was measured by using a video tracking system. Spatial learning training was 4 trials per day for continuous 6 days. During the training, each animal was gently released into the water tank by facing the wall, from four different quadrants in 4 training trials, with 10‐min interatrial intervals. The animal was allowed to find the hidden platform within 60 s. If a mouse found the hidden platform within 60 s, it was allowed staying on the platform for extra 10 s. If a mouse failed to escape to the hidden platform within 60 s, it was guided on to and stayed on the platform for extra 15 s. The mean time cross 4 trials per day in escaping onto the hidden platform was used to score spatial learning on that day. Spatial memory was tested by a retrieval test, i.e., a probe trial that was conducted at 24‐h after the final training trial without the hidden platform. Each mouse was released into the water tank by facing the wall from the diagonal quadrant of the original one where the hidden platform was placed previously, and then the mouse was allowed freely swimming for 60 s. The time spent in the target quadrant and entries to the target location where the hidden platform was previously placed were used to score spatial memory. All the data in the Morris water maze were recorded and analyzed by using a video tracking system, the EnthoVision 8.0 program (from Noldus, Beijing, China).

### Tissue Collection for Spatial Transcriptomics and Immunofluorescence

The brains were collected from WT mice at 4‐month‐old (4WT) and 24‐month‐old (24WT) under control diet (normal pellets), and from AD mice at 24‐month‐old treated with SL (normal pellets mixed with estimated 20 mg kg day from 14 to 24‐month‐old) (24SL) or control diet (24AD). The four group mice were anesthetized and the brains were obtained quickly, followed by separating the left hemisphere from the right one with a surgical blade at a 45‐degree angle. The left hemisphere was post‐fixed in paraformaldehyde (PFA) solution at 4°C for slicing and immunofluorescence study. The right hemisphere was transferred into liquid nitrogen‐cool isopentane for 15 s and then removed out and embedded in optimal cutting temperature (OCT) for frozen sectioning, and then used for spatial transcriptomics study.

### Tissue Collection for Immunoblotting and Immunofluorescent Studies on the Hippocampus

The brain tissues were collected from AD mice and their controls at the ages from 10 to 24‐month‐old, and the others were collected from C57BL/6J at 3‐month‐old. After anesthesia, the mice were subjected to myocardial perfusion with saline for 5 min. The brains were obtained quickly, followed by separating the left hemisphere from the right one with a surgical blade at a 45‐degree angle. The left hemisphere was post‐fixed in PFA solution at 4°C for frozen sectioning and immunofluorescent studies. The right hemisphere was stored at −80 °C until being processed.

### Spatial Transcriptomics

The spatial transcriptomics was performed by following 10X Visium protocol (version CG000160). Briefly, the mouse brains were sectioned in a pre‐cooled cryostat (−18°C, HM 525 NX) at the thickness of 16 µm until the coronal sections well reached to the targeted hippocampal region, i.e., 2.03 mm posterior to the bregma. The slices were mounted on pre‐cooled Visium Spatial Gene Expression slides, and the slices were fixed and stained by hematoxylin‐eosin with immediate imaging at 20X magnification. Then the tissues underwent permeabilization for 15 min that was determined by a preliminary study, and the spatially barcoded libraries were obtained from reverse transcription and cDNA amplification, in which the qPCR (Bio‐Rad, CFX96 Touch) was used to determine the amplification round in each sample. The constructed libraries were sequenced under Nova‐seq PE150 platform. The summary on 10X Visium slide numbers.

The selection criteria were applied a posteriori and were based on the quantitative and qualitative results from the IHC analysis. Specifically, inclusion was determined by the average immunofluorescence intensity of Aβ plaque load in conjunction with qualitative expression of Glut1.

### Data Processing and Brain Region Annotation

The paired‐end reads were mapped to the mouse reference genome mm10 (https://cf.10xgenomics.com/supp/spatial‐exp/refdata‐gex‐mm10‐2020‐A.tar.gz) using Space Ranger (version 1.3.0) (https://www.10xgenomics.com/support/software/space‐ranger/downloads/previous‐versions) with default parameters. Then the functions implemented in Seurat package (version 4.3.0) were used in the following analysis if without explicit statement.^[^ ^40^ ^]^ First, the Load10X_Spatial function was used to load the aligned spatial transcriptomics data in each sample. The spots with less than 200 expressed genes was removed and the spots with more than 30% of unique molecular identifiers (UMIs) corresponding to mitochondrial genes. Totally, 11 448 spots were left in the four samples after data quality control (see Figure S3a–d, Supporting Information). The mitochondrial genes were removed from all following analyses. The SCTranform function was used to normalize gene expressions in each spot by using the parameter: vars.to.regress = “percent.mt”. The software SVGbit (version 0.0.4) was used to select the top 500 spatially variable genes in each sample.^[^ ^25^ ^]^ These top spatially variable genes in the four samples were merged together, yielding 1090 genes in total. With these selected genes, FindIntegrationAnchors function with default parameters to identify anchors was used, and then used IntegrateData function to combine the samples. The Louvain algorithm implemented in FindClusters function was used to cluster spots by using the 13 principal components derived from RunPCA function. 14 clusters were obtained, and FindAllMarkers function in Seurat was next used to detect the marker genes in each cluster with parameters: Log2FC ≥ 0.25 and *p* < 0.05 (see Figure S4a, Supporting Information). According to the selected marker genes and the brain reference from the Paxinos and Franklin's the Mouse Brain in Stereotaxic Coordinates (4th Edition), 8 well‐known regions [Isocortex layer 1/2 was finally defined, layer 3/4, layer 5, layer 6, and CA1, CA2/3, GrDG (the granular layer of the dentate gyrus), dendritic region of the hippocampus], and 6 ambiguous regions (named C0 to C5).

### AAR Expression Pattern

In each brain region, differentially expressed genes (DEGs) were detected among the 4 samples in the paired ways according to aging and disease progression (4WT vs 24WT, 24WT vs 24AD, 24AD vs 24SL) using Wilcoxon rank‐sum test with 0.001 as the threshold of false discovery rate (FDR). The overlapped DEGs from the three comparative ways were used in the following analysis. The DEGs exhibit different kinds of upregulation and downregulation patterns among the four samples (4WT, 24WT, 24AD, and 24SL). In these variation patterns, one predominant expression pattern was found, in which gene expression was increased in WT mice during aging (4WT vs 24WT, aging related effect) and decreased in aged AD mice (24WT vs 24AD, AD related effect) but rescued in AD mice after SL treatment (24AD vs 24SL, SL related effect), to a level similar to WT's. Therefore, this expression pattern as Aging‐AD‐Rescue (AAR) expression pattern was defined, which may reflect a fundamental rule of the DEGs contributable to aging, AD, and rescue related effects.

### Enrichment Analysis

Enrichment analysis of Kyoto Encyclopedia of Genes and Genomes (KEGG)^[^ ^41^ ^]^ and Gene Ontology (GO)^[^ ^42^ ^]^ were performed by using R package clusterProfiler (version 4.4.2),^[^ ^43^ ^]^ in which the hypergeometric test was used to analyze the major functions or annotations of target gene sets. Gene set variation analysis (GSVA) was performed.^[^ ^44^ ^]^ All gene expressions in KEGG pathways were used to compute the pathway activity scores, and the limma package was used to select the differential pathways in the three pairwise comparisons (4WT vs 24WT, 24WT vs 24AD, 24AD vs 24SL) with parameters: *p* < 0.05 and t > 2. For each pathway, if GSVA scores among four samples (4WT, 24WT, 24AD, and 24SL) showed a consistent AAR expression pattern, this pathway was classified as an AAR pathway.^[^ ^45^ ^]^

### Molecule Docking

The 3D structures of human protein HRAS, MAPK1 and ADD1 were obtained from PDB database (https://www.rcsb.org/) with ID number 2QUZ, 6QA3, and AF935611 respectively. The water molecules were removed from the protein structures using the software PyMol (https://pymol.org/2/) and then the SL (SL‐ZF‐01) was docked into proteins using AutoDockTool4.^[^ ^46^ ^]^ Specifically, the 2D format of SL was transformed to mol2 format by PyMol and hydrogens were added, and then this molecule was used as ligand to detect the Torsion root for 3D conformation prediction. Each protein was pre‐processed as following steps: adding hydrogens, computing gasteiger, assigning AD4 type, choosing it as receptor and putting it into a dock grid box. After the preliminary process, the SL was docked into protein with default parameters using open‐source program AutoDockVina which is integrated in AutoDockTools.^[^ ^47^, ^48^ ^]^ Finally, the docking results were visualized by PyMol.

### Human Proteome Microarray Assays

Profiling of SL‐ZF‐01 interacting proteins was conducted on HuProt™20K Human Proteome Microarrays (CDI Laboratories). The entire procedure, comprising chip hybridization, washing, and signal detection, was carried out in strict adherence to the vendor's standardized operating procedures.^[^ ^49^ ^]^

### Immunofluorescence Study and Image Capture

The experimental protocols were similar to those described previously.^[^ ^18^, ^50^ ^]^ The PFA‐fixed left hemisphere was cut into coronal slices at 40 µm thickness by using a vibrotome (VT‐1000S, Leica). The slices were stored cryogenically at −20 °C in cryopreservation solution with 20% ethylene glycol and 30% glycerol. Three to five coronal sections of each mouse were selected to stain Aβ, Glut1 (glucose transporter type 1), GFAP (glial fibrillary acidic protein) for glial cells, and lectin for microvessels (≤ 20 µm) in the brain.

After transferred to sifters inserted into a six‐well plate, the slices were washed 3 times by phosphate‐buffered saline with Tween ® detergent (PBST) for 30 min. After that, the slices were transferred into 5% bovine serum albumin (BSA) and 1% Triton X‐100 in phosphate buffered saline (PBS) solution for the incubation for 60 min. Then, the slices were transferred to primary antibodies overnight at 4 °C. On the next day, the slices were washed 3 times by PBST for 30 min, followed by incubation of second antibodies conjugated with different fluorophores for 120 min. Next, the slices were washed 3 times by PBST for 30 min and were mounted on microscope slides in PBS solution. After PBS solution dried, one drop of the Antifade Mounting Medium with DAPI was added on to the slices and a coverslip was carefully placed on. Images were captured by Olympus FV3000 microscope.

Animals at 10‐month‐old were used in this study. Older mice at 17 or 24‐month‐old were also used for this study, but lectin signals were very bad and thus the data were not shown in the present report, implying that some unknown changes occurred in the brain microvessels in AD mice at late stages.

### Immunoblotting and Protein Quantifications

The experimental protocols followed to those described previously.^[^ ^51^ ^]^ As usually, 200 µL RIPA lysate buffer with cocktail protease inhibitors and cocktail phosphatase inhibitors were added into 20 mg hippocampal tissue and homogenized with zirconia beads for 60 s. The supernatants were added with SDS‐PAGE loading buffer without heating according to antibody manufacturer recommendations. Then, the protein samples were separated on a 10% SDS‐PAGE gel (PAGE Gel Fast Preparation Kit, Epizyme), and transferred to PVDF membranes with wet transfers. Blots were subjected to immunoblotting analysis using the following antibodies: mouse anti‐Glucose Transporter Glut1 (1: 500, abcam), rabbit anti‐Glucose Transporter Glut3 (1: 1000, abcam), goat anti‐CD31/PECAM‐1 (1: 50, R&D), HRP‐conjugated Monoclonal Mouse Anti‐glyceraldehyde‐3‐phosphate Dehydrogease GAPDH (1: 2000, DBA Aksomics). The secondary antibodies were HRP‐conjugated Affinipure Goat Anti‐Mouse IgG (1:1000, proteintech), HRP‐conjugated Affinipure Goat Anti‐Rabbit IgG (1: 1000, proteintech), and HRP‐conjugated Affinipure Rabbit Anti‐Goat IgG (1: 1000, proteintech), respectively. Proteins were visualized using immmobilon western chemilum HRP substrate (Millipore). Imaging acquisition were performed on a Tanon‐5200 Multi Automatic chemiluminescence image analysis system. Quantitation of bands were performed using the Image J software. (Some of the protein samples for immunoblotting were the rest samples from ATP level test)

### Detection of ATP Levels

Detection of ATP levels and BCA protein quantification both followed the manufacturer (Beyotime)’s instruction: https://m.beyotime.com/mobilegoods.do?method = code&code = S0027. ATP quantification was conducted with an Enhanced ATP Assay kit (Beyotime). For details, 200 µL lysate buffer with cocktail protease inhibitors and cocktail phosphatase inhibitors were added into 20 mg hippocampal tissue and homogenized with zirconia beads for 60 s. 100 µL ATP test reagent was added into a 96‐well black plates with flat bottom wells. After incubation for 3 min at room temperature, 20 µL obtained supernatant or ATP standard solution were transferred to the ATP test reagent well. ATP levels were detected by chemiluminescence using Multi‐Mode Microplate Reader (Feyond‐A300, ALLSHENG). Protein quantification was conducted with a BCA Protein Assay Kit (Beyotime). The relative ATP levels were calculated according to the following formula: relative ATP levels = ATP values/protein concentration.

### Statistical Analysis

Data preprocessing for the Morris water maze behavioral test involved the assessment and exclusion of outliers. The initial group sizes were *n* = 21 for wild‐type (WT), AD and SL‐treated AD (SL) group. Two mice from the SL‐treated group were identified as outliers and excluded from all subsequent analyses because two SL‐treated mice were very healthy and floated in the water without swimming in the Morris water maze test. For Western blot analysis, which included samples from WT, AD, and SL groups (or from 4‐, 12‐, and 24‐month‐old WT), biological replicates were processed on separate membranes. To control for technical variation across these membranes, the data were normalized. Specifically, samples were grouped into matched sets of three, and the optical density values for the WT sample within each set were used as the reference for normalization.

All data are presented as the mean ± SEM. Statistical analyses were performed using GraphPad Prism 9.0 software. Details of the specific statistical tests applied are provided in the figure legends.

Differential Gene Expression Analysis: Differentially expressed genes (DEGs) were detected among the 4 samples in pairwise comparisons according to aging, AD, and rescue by SL (4WT vs 24WT, 24WT vs 24AD, 24AD vs 24SL) by using Wilcoxon rank‐sum test with p < 0.001 as the significant threshold after correction by false discovery rate (FDR). Behavioral and Biochemical Data Analysis: For comparisons of means across three or more groups (e.g., WT, AD, SL), one‐way analysis of variance (ANOVA) was used. The selection of post‐hoc tests was based on the experimental design: Dunnett's post‐hoc test was used when comparisons were focused against a single control group (e.g., all groups vs. WT). Tukey's post‐hoc test was used for all pairwise comparisons between groups. For data that violated the assumption of homogeneity of variances, Welch's ANOVA was applied, followed by Dunnett's T3 multiple comparisons test. Western blot densities were analyzed with the Kruskal‐Wallis test followed by Dunn‐post hoc test. A p‐value of ^*^ *p* < 0.05 was considered statistically significant, with ^*^ *p* < 0.05, ^**^ *p* < 0.01, and ^***^ *p* < 0.001.

## Conflict of Interest

Some authors (L.X., F.L., J.F.H., Q.X.Z., L.J.F., C.P., and Y.L.T.) applied a patent for ferul enanthate PCT/CN2024/080334. All other authors declare no competing interests.

## Author Contributions

F.L. and Y.‐L.T. contributed equally to this work. Q.X.Z., C.P., L.X., and C.M.G. conceptualized and designed this study. J.F.L., J.F.H., and Y.Q.D. designed and synthesized ferul enanthate derivatives. F.L. optimized and performed experiments, together with Z.B.Z., Y.H.T., S.H.L., N.Y.W., J.N.L., Z.J.P. And Y.L.T. performed data analysis, and C.P. supervised data analysis. The manuscript was written and revised by F.L., Y.L.T., Q.X.Z., J.F.H., Y.Q.D., C.P., L.X., and C.M.G. We thank all members from C.P. and L.X.’s laboratories for their support.

## Supporting information



Supporting Information

Supporting Information

Supporting Information

## Data Availability

The 10X Visium data, including the raw sequencing reads, H&E images and slide layout files, have been deposited in the National Genomics Data Center (https://ngdc.cncb.ac.cn) with the BioProject accession number PRJCA023205, in which the sequencing reads are under GSA accession number CRA014696, and the H&E images are under OMIX accession number OMIX005854. The original code is publicly available at https://github.com/tyyl622/ADmouse/tree/main.

## References

[1] G. G Glenner , C. W. Wong , Biochem. Biophys. Res. Commun. 1984, 120, 885. 6375662 10.1016/s0006-291x(84)80190-4

[2] J. A. Hardy , G. A. Higgins , Science 1992, 256, 1845. 10.1126/science.1566067 1566067

[3] Y. Zhang , H. Chen , R. Li , K. Sterling , W. Song , Signal Transduction Targeted Ther. 2023, 8, 248. 10.1038/s41392-023-01484-7 PMC10310781 37386015

[4] A. L. Boxer , R. Sperling , Cell 2023, 186, 4757. 37848035 10.1016/j.cell.2023.09.023 PMC10625460

[5] M. A. Better , Alzheimers Dement. 2023, 19, 1598.36918389

[6] I. Driscoll , J. Troncoso , Curr. Alzheimer Res. 2011, 8, 330. 21222594 10.2174/156720511795745348 PMC3286868

[7] M. H. Murdock , L.‐H. Tsai , Nat. Neurosci. 2023, 26, 181. 36593328 10.1038/s41593-022-01222-2 PMC10155598

[8] B. J Graff , S. L Harrison , S. J. Payne , W. K. El‐Bouri , Cerebrovascular Diseases 2023, 52, 11. 35640565 10.1159/000524797

[9] J. De la Torre , Neurosci. Biobehav. Rev. 1994, 18, 397. 7984357 10.1016/0149-7634(94)90052-3

[10] S. Hunt , J. P. Hellwig , Nursing for Women's Health 2018, 22, 16.

[11] D. Kellar , S. Craft , Lancet Neurol. 2020, 19, 758. 32730766 10.1016/S1474-4422(20)30231-3 PMC9661919

[12] N. Kyrtata , H. C Emsley , O. Sparasci , L. M. Parkes , B. R. Dickie , Front. Neurosci. 2021, 15, 626636. 34093108 10.3389/fnins.2021.626636 PMC8173065

[13] S. Raut , A. Bhalerao , M. Powers , M. Gonzalez , S. Mancuso , L. Cucullo , Cells 2023, 12, 2019. 37626828 10.3390/cells12162019 PMC10453773

[14] K. Zeller , S. Rahner‐Welsch , W. Kuschinsky , J. Cereb. Blood Flow Metab. 1997, 17, 204. 9040500 10.1097/00004647-199702000-00010

[15] S. C Cunnane , E. Trushina , C. Morland , A. Prigione , G. Casadesus , Z. B Andrews , M. F Beal , L. H Bergersen , R. D. Brinton , S. De La Monte , Nat. Rev. Drug Discovery 2020, 19, 609. 32709961 10.1038/s41573-020-0072-x PMC7948516

[16] J. Yuan , S.‐Y Chang , S.‐G Yin , Z.‐Y Liu , X. Cheng , X.‐J Liu , Q. Jiang , G. Gao , D.‐Y. Lin , X.‐L. Kang , Nature 2020, 579, 118. 32103178 10.1038/s41586-020-2037-y

[17] B. Michels , H. Zwaka , R. Bartels , O. Lushchak , K. Franke , T. Endres , M. Fendt , I. Song , M. Bakr , T. Budragchaa , Sci. Adv. 2018, 4, aat6994. 10.1126/sciadv.aat6994 PMC6224069 30417089

[18] N.‐Y Wang , J.‐N Li , W.‐L Liu , Q. Huang , W.‐X Li , Y.‐H Tan , F. Liu , Z.‐H Song , M.‐Y. Wang , N. Xie , Neurotherapeutics 2021, 18, 1064. 33786807 10.1007/s13311-021-01024-7 PMC8423929

[19] C. B Castro , C. B Dias , H. Hillebrandt , H. R Sohrabi , P. Chatterjee , T. M Shah , S. J Fuller , M. L. Garg , R. N. Martins , Nutr. Rev. 2023, 81, 1144. 36633304 10.1093/nutrit/nuac104

[20] F. Mochel , E. Hainque , D. Gras , I. M Adanyeguh , S. Caillet , B. Héron , A. Roubertie , E. Kaphan , R. Valabregue , D. Rinaldi , J. Neurol., Neurosurg. Psychiatry. 2016, 87, 550. 26536893 10.1136/jnnp-2015-311475 PMC4853553

[21] G. Paxinos , K. B. Franklin , Paxinos and Franklin's the Mouse Brain in Stereotaxic Coordinates, Academic Press, Cambridge, Massachusetts 2019.

[22] R. G Morris , P. Garrud , J. Rawlins , J. O'Keefe , Nature 1982, 297, 681. 7088155 10.1038/297681a0

[23] W.‐T Chen , A. Lu , K. Craessaerts , B. Pavie , C. S Frigerio , N. Corthout , X. Qian , J. Laláková , M. Kühnemund , I. Voytyuk , Cell 2020, 182, 976. 32702314 10.1016/j.cell.2020.06.038

[24] O. Hahn , A. G Foltz , M. Atkins , B. Kedir , P. Moran‐Losada , I. H Guldner , C. Munson , F. Kern , R. Pálovics , N. Lu , Cell 2023, 186, 4117. 37591239 10.1016/j.cell.2023.07.027 PMC10528304

[25] Y. Hong , K. Song , Z. Zhang , Y. Deng , X. Zhang , J. Zhao , J. Jiang , Q. Zhang , C. Guo , C. Peng , Cell Death Discovery 2023, 9, 264. 37500639 10.1038/s41420-023-01569-w PMC10374563

[26] S. J Martin , P. D. Grimwood , R. G. Morris , Annu. Rev. Neurosci. 2000, 23, 649. 10845078 10.1146/annurev.neuro.23.1.649

[27] K. Hotta , N. L Bodkin , T. A Gustafson , S. Yoshioka , H. K. Ortmeyer , B. C. Hansen , J. Gerontol. A. Biol. Sci. Med. Sci. 1999, 54, B183. 10361996 10.1093/gerona/54.5.b183

[28] I. Ferrer , P. Andrés‐Benito , K. Ausín , R. Pamplona , J. A Del Rio , J. Fernández‐Irigoyen , E. Santamaría , Brain Pathol. 2021, 31, 12996. 10.1111/bpa.12996 PMC8549032 34218486

[29] G. G. Glenner , C. W. Wong , Biochem. Biophys. Res. Commun. 1984, 120, 885. 6375662 10.1016/s0006-291x(84)80190-4

[30] J. L. Cummings , T. Morstorf , K. Zhong , Alzheimer's Res. Ther. 2014, 6, 37. 25024750 10.1186/alzrt269 PMC4095696

[31] C. H. van Dyck , C. J. Swanson , P. Aisen , R. J. Bateman , C. Chen , M. Gee , M. Kanekiyo , D. Li , L. Reyderman , S. Cohen , L. Froelich , S. Katayama , M. Sabbagh , B. Vellas , D. Watson , S. Dhadda , M. Irizarry , L. D. Kramer , T. Iwatsubo , N. Engl. J. Med. 2023, 388, 9. 36449413 10.1056/NEJMoa2212948

[32] W. T. Chen , A. Lu , K. Craessaerts , B. Pavie , C. Sala Frigerio , N. Corthout , X. Qian , J. Laláková , M. Kühnemund , I. Voytyuk , L. Wolfs , R. Mancuso , E. Salta , S. Balusu , A. Snellinx , S. Munck , A. Jurek , J. Fernandez Navarro , T. C. Saido , I. Huitinga , J. Lundeberg , M. Fiers , B. De Strooper , Cell 2020, 182, 976. 32702314 10.1016/j.cell.2020.06.038

[33] N. Kyrtata , H. C. A. Emsley , O. Sparasci , L. M. Parkes , B. R. Dickie , Front Neurosci. 2021, 15, 626636. 34093108 10.3389/fnins.2021.626636 PMC8173065

[34] E. A. Winkler , Y. Nishida , A. P. Sagare , S. V. Rege , R. D. Bell , D. Perlmutter , J. D. Sengillo , S. Hillman , P. Kong , A. R. Nelson , J. S. Sullivan , Z. Zhao , H. J. Meiselman , R. B. Wendy , J. Soto , E. D. Abel , J. Makshanoff , E. Zuniga , D. C. De Vivo , B. V. Zlokovic , Nat. Neurosci. 2015, 18, 521. 25730668 10.1038/nn.3966 PMC4734893

[35] H. M. Al‐Kuraishy , M. S. Jabir , A. K. Albuhadily , A. I. Al‐Gareeb , S. F. Jawad , A. A. Swelum , N. R. Hadi , Ageing Res. Rev. 2024, 95, 102233. 38360180 10.1016/j.arr.2024.102233

[36] P. E. H. M'Bra , L. K. Hamilton , G. Moquin‐Beaudry , C. L. Mangahas , F. Pratesi , A. Castonguay , S. Mailloux , M. Galoppin , J. Avila Lopez , M. Bernier , M. Turri , M. Mayhue , A. Aumont , M. Tétreault , S. C. Cunnane , K. J. L. Fernandes , Brain 2025, 10.1093/brain/awaf267. PMC12782165 40794774

[37] C. R. Jack , D. S. Knopman , W. J. Jagust , R. C. Petersen , M. W. Weiner , P. S. Aisen , L. M. Shaw , P. Vemuri , H. J. Wiste , S. D. Weigand , T. G. Lesnick , V. S. Pankratz , M. C. Donohue , J. Q. Trojanowski , Lancet Neurol 2013, 12, 207. 23332364 10.1016/S1474-4422(12)70291-0 PMC3622225

[38] M. C. Donohue , R. A. Sperling , D. P. Salmon , D. M. Rentz , R. Raman , R. G. Thomas , M. Weiner , P. S. Aisen , JAMA Neurol. 2014, 71, 961. 24886908 10.1001/jamaneurol.2014.803 PMC4439182

[39] M. Askenazi , T. Kavanagh , G. Pires , B. Ueberheide , T. Wisniewski , E. Drummond , Nat. Commun. 2023, 14, 4466. 37491476 10.1038/s41467-023-40208-x PMC10368642

[40] Y. Hao , S. Hao , E. Andersen‐Nissen , W. M Mauck , S. Zheng , A. Butler , M. J Lee , A. J Wilk , C. Darby , M. Zager , Cell 2021, 184, 3573. 34062119 10.1016/j.cell.2021.04.048 PMC8238499

[41] M. Kanehisa , S. Goto , Nucleic Acids Res. 2000, 28, 27. 10592173 10.1093/nar/28.1.27 PMC102409

[42] M. Ashburner , C. A Ball , J. A Blake , D. Botstein , H. Butler , J. M Cherry , A. P Davis , K. Dolinski , S. S. Dwight , J. T. Eppig , Nat. Genet. 2000, 25, 25. 10802651 10.1038/75556 PMC3037419

[43] T. Wu , E. Hu , S. Xu , M. Chen , P. Guo , Z. Dai , T. Feng , L. Zhou , W. Tang , L. Zhan , The Innovation 2021, 2, 100141. 34557778 10.1016/j.xinn.2021.100141 PMC8454663

[44] S. Hänzelmann , R. Castelo , J. Guinney , BMC Bioinformatics 2013, 14, 7. 23323831 10.1186/1471-2105-14-7 PMC3618321

[45] M. E Ritchie , B. Phipson , D. Wu , Y. Hu , C. W Law , W. Shi , G. K. Smyth , Nucleic Acids Res. 2015, 43, 47. 10.1093/nar/gkv007 PMC4402510 25605792

[46] G. M Morris , R. Huey , W. Lindstrom , M. F Sanner , R. K Belew , D. S. Goodsell , A. J. Olson , J. Comput. Chem. 2009, 30, 2785. 19399780 10.1002/jcc.21256 PMC2760638

[47] J. Eberhardt , D. Santos‐Martins , A. F. Tillack , S. Forli , J. Chem. Inf. Model. 2021, 61, 3891. 34278794 10.1021/acs.jcim.1c00203 PMC10683950

[48] O. Trott , A. J. Olson , J. Comput. Chem. 2010, 31, 455. 19499576 10.1002/jcc.21334 PMC3041641

[49] S. Ye , W. Luo , Z. A Khan , G. Wu , L. Xuan , P. Shan , K. Lin , T. Chen , J. Wang , X. Hu , S. Wang , W. Huang , G. Liang , Circulation Res. 2020, 126, 1007. 32098592 10.1161/CIRCRESAHA.119.315861

[50] J. Zheng , Methods Mol. Biol. 1998, 105, 307. 10427573 10.1385/0-89603-491-7:307

[51] R. Sule , G. Rivera , A. V. Gomes , BioTechniques 2023, 75, 99. 36971113 10.2144/btn-2022-0034 PMC12303220

