# Supplementary material for: Modifying Glucose Metabolism Reverses Memory Defects of Alzheimer's Disease Model at Late Stages
Source: Adv Sci (Weinh). 2025 Dec 8;13(11):e06695. doi: 10.1002/advs.202506695 (PMC12931168; doi:10.1002/advs.202506695)
Supplement: Supplementary file 1 — Supporting Information [file ADVS-13-e06695-s001.docx]

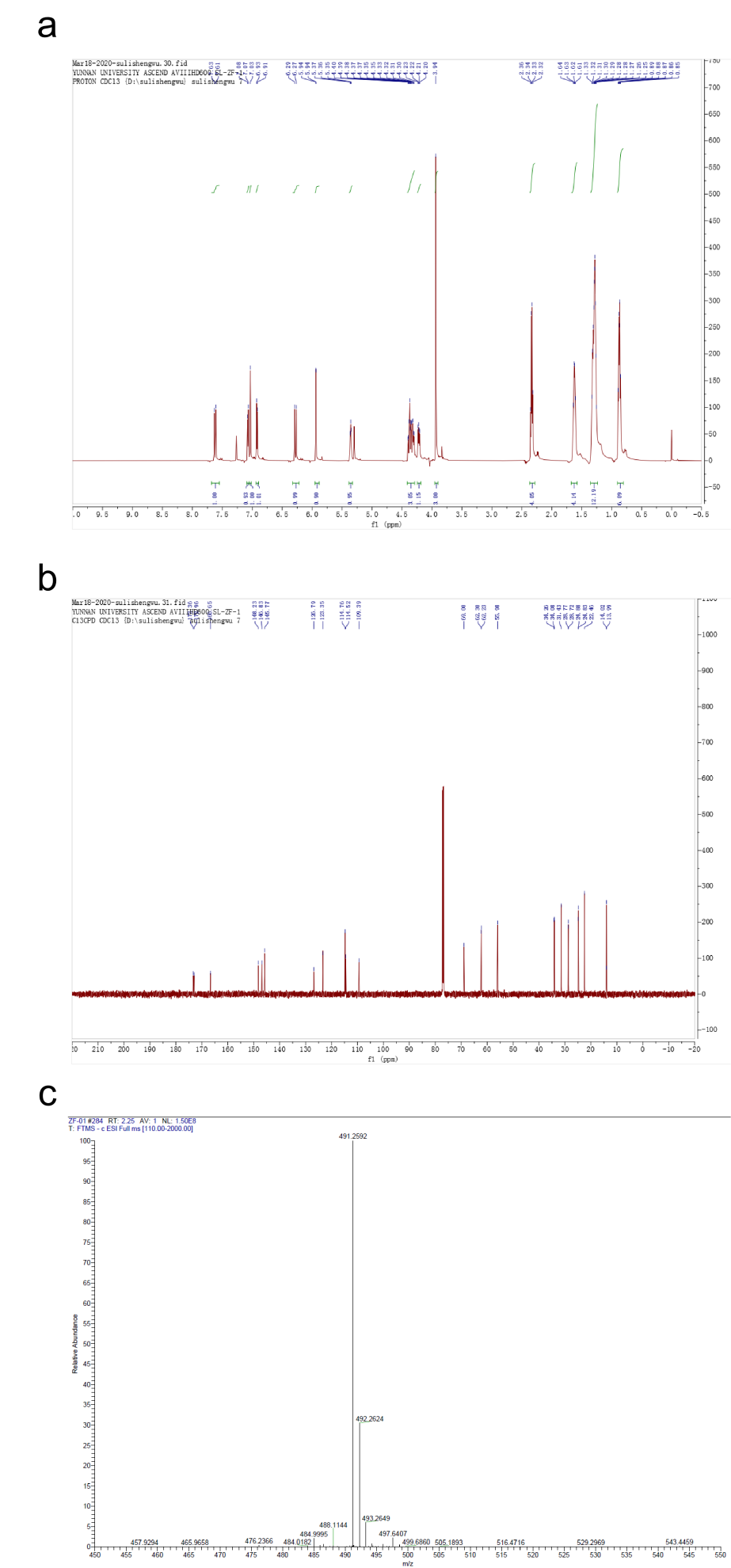


**Figure S1. SL-ZF-01**

1. 1 H NMR spectrum of SL.
2. 13 C NMR spectrum of SL.
3. Mass spectrometry of SL.


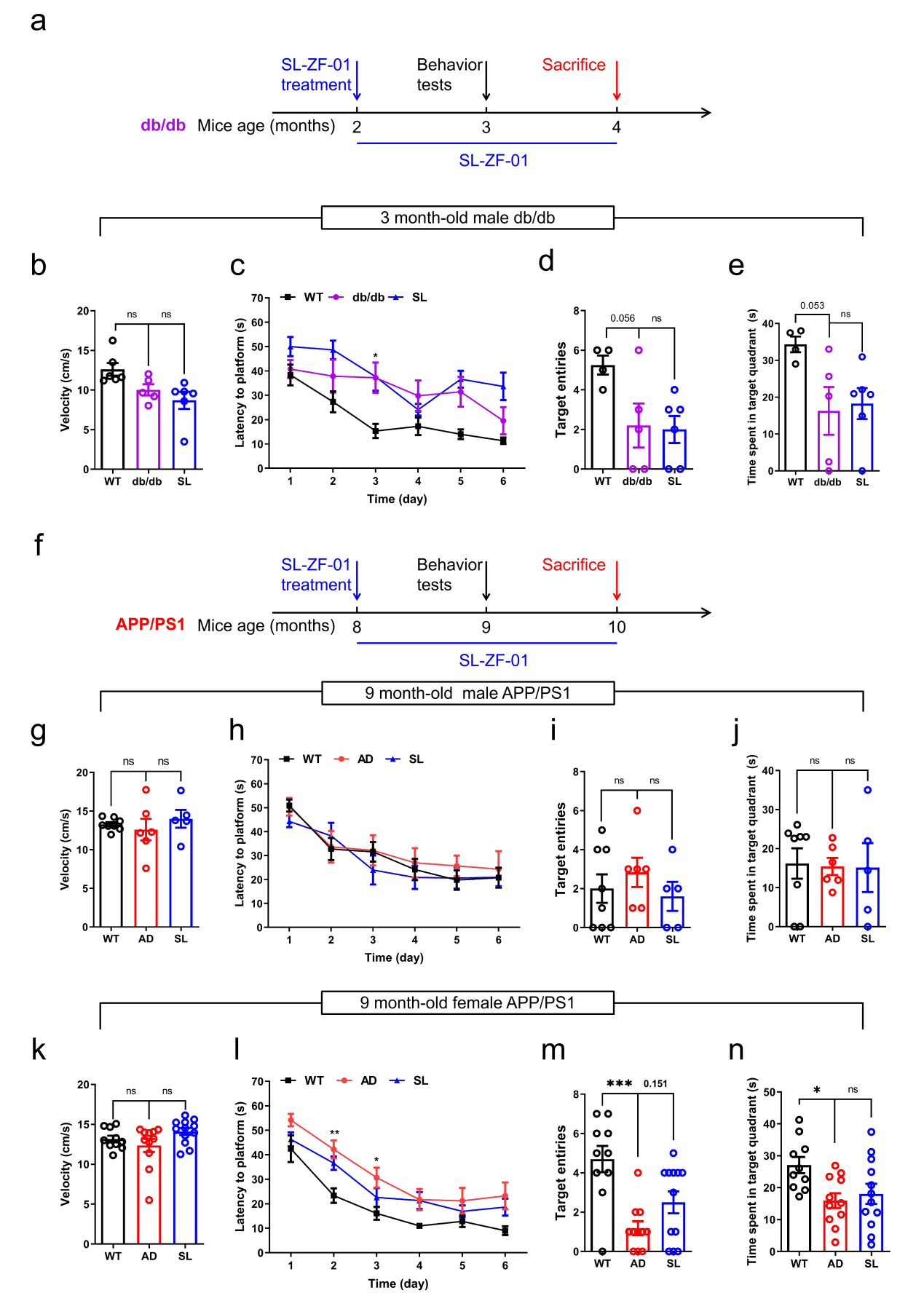


**Figure S2. SL was tested in db/db and AD mice.**

**a.** db/db mice (a model of insulin resistance) were administered SL-ZF-01 (SL; 20 mg/kg/day) via diet for one month (SL group), with age-matched, pellet-fed db/db and wild-type (WT) mice serving as disease and healthy controls, respectively. All mice used here were male.

**b-e.** The spatial learning task and probe trial at 24-h after the final training of the Morris water maze were used to test the effects of SL on the hippocampus-dependent learning and memory. SL treatment had no effects on swimming velocity, latency to platform as well as target entries and target quadrant time (*p* > 0.05 in panels b-e, db/db vs. SL), although db/db group exhibited a trend of impaired spatial learning and memory (*p* = 0.056 in panel d and e, WT vs. db/db).

**f.** Experimental protocols for screening the effects of SL for AD mice. SL treatment was similar to panel a.

**g-j.** SL treatment had no effect on swimming velocity, latency to platform as well as target entries and target quadrant time, suggesting that SL produced no effect on spatial learning and memory in male AD mice at 9-month-old. All mice used here were male.

**k-n.** SL produced a trend of improving spatial learning and memory in female AD mice at 9-month-old. All mice used here were female.

**p* < 0.05; ***p* < 0.01; ****p* < 0.001 by two-way ANOVA with Dunnett’s multiple comparisons test (c, h, l), or one-way ANOVA with Tukey’s multiple comparisons test (b, d, e, g, i, j, k, m, n). Data are presented as mean ± SEM.


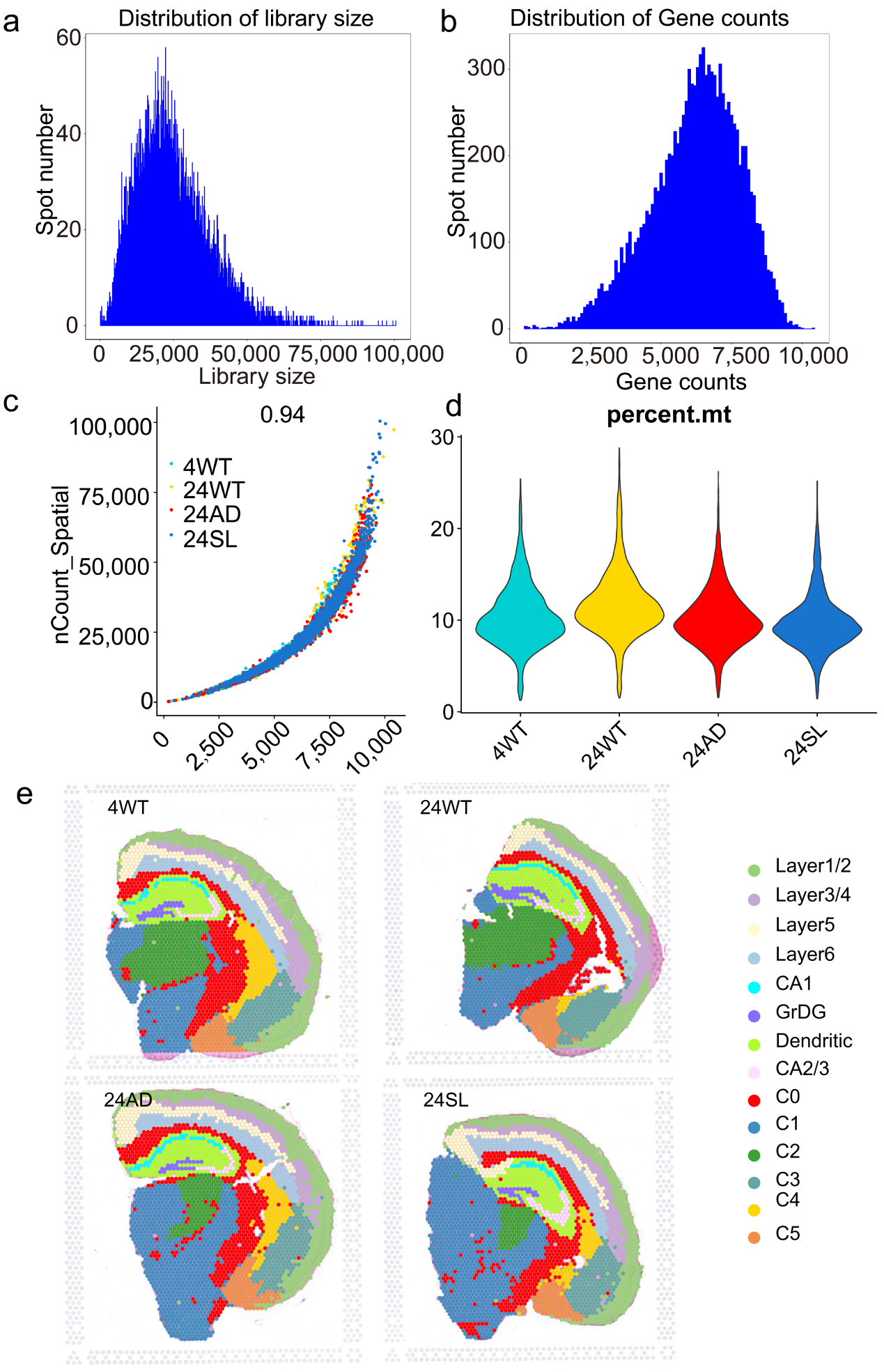


**Figure S3. Quality control for spatial transcriptomics data.**

**a-b.** Distribution of library size (a) and gene counts (b).

**c.** The correlation between the number of features and the summary of features counts in every spot is 0.94.

**d.** The percentages of mitochondrial genes (percent.mt) in the 4 samples (4WT, 24WT, 24AD, and 24SL).

**e.** The brain spatial domains of the 4 samples. The 24AD sample was further used to depict detailed brain regions in Fig. 2f.


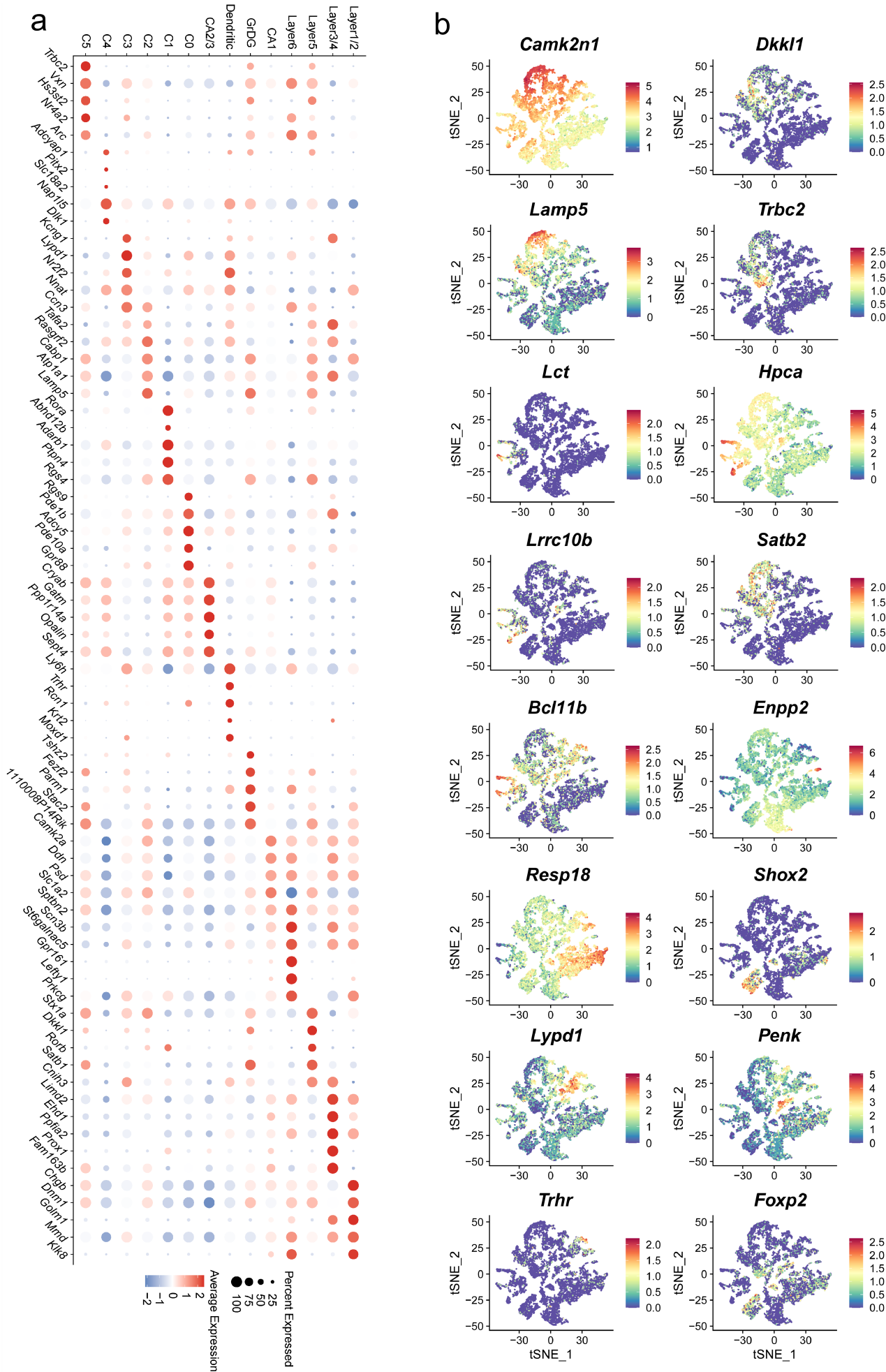


**Figure S4. Brain region-specific marker genes were visualized by dot plot and tSNE embedding**

**a.** Dot plot showing the top 5 brain region-specific marker genes.

**b.** The t-distributed stochastic neighbour embedding visualized the brain region-specific marker genes. The representative brain region-specific genes, *Camk2n1* (Calcium/calmodulin-dependent protein kinase II inhibitor 1, a regulator of neuronal signaling and plasticity, often expressed in hippocampal and cortical regions), *Dkkl1* (Dickkopf-like 1, a modulator of Wnt signaling pathways involved in brain development and neurogenesis, expressed in layer3/4 and Layer 5), *Lamp5* (Lysosomal-associated membrane protein 5, enriched in specific neuronal subtypes, such as inhibitory interneurons, expressed in Layer1/2), *Trbc2* (T cell receptor beta constant 2, primarily immune-related, expressed in Layer 6), *Lct* (Lactase, expressed in GrDG), *Hpca* (Hippocalcin, a calcium-binding protein highly specific to hippocampal neurons and involved in synaptic plasticity), *Lrrc10b* (Leucine rich repeat containing 10B, highly expressed in CA2/3), *Satb2* (SATB homeobox 2, a critical transcription factor for cortical neuron differentiation and a biomarker of upper-layer neurons), *Bcl11b* (B-cell lymphoma/leukemia 11B, a transcription factor essential for corticospinal neuron specification and layer V identity), *Enpp2* (Ectonucleotide pyrophosphatase/phosphodiesterase 2, also known as autotaxin, involved in lipid signaling and highly expressed in C0), *Resp18* (Regulated endocrine-specific protein 18, associated with neuroendocrine functions and potentially enriched in C1 and C5), *Shox2* (Short stature homeobox 2, expressed in C2), *Lypd1* (Ly6/PLAUR domain containing 1, a GPI-anchored protein potentially involved in cholinergic signaling and neuronal identity, expressed in C3), *Penk* (Proenkephalin, a precursor for opioid peptides widely expressed in striatal and limbic systems, expressed in C4), Trhr (Thyrotropin-releasing hormone receptor, expressed in C5), and *Foxp2* (Forkhead box P2, a transcription factor critical for language development and neuronal connectivity in cortical and basal ganglia regions, expressed in C2 C4 and Layer6) were also separated from each other by tSNE.


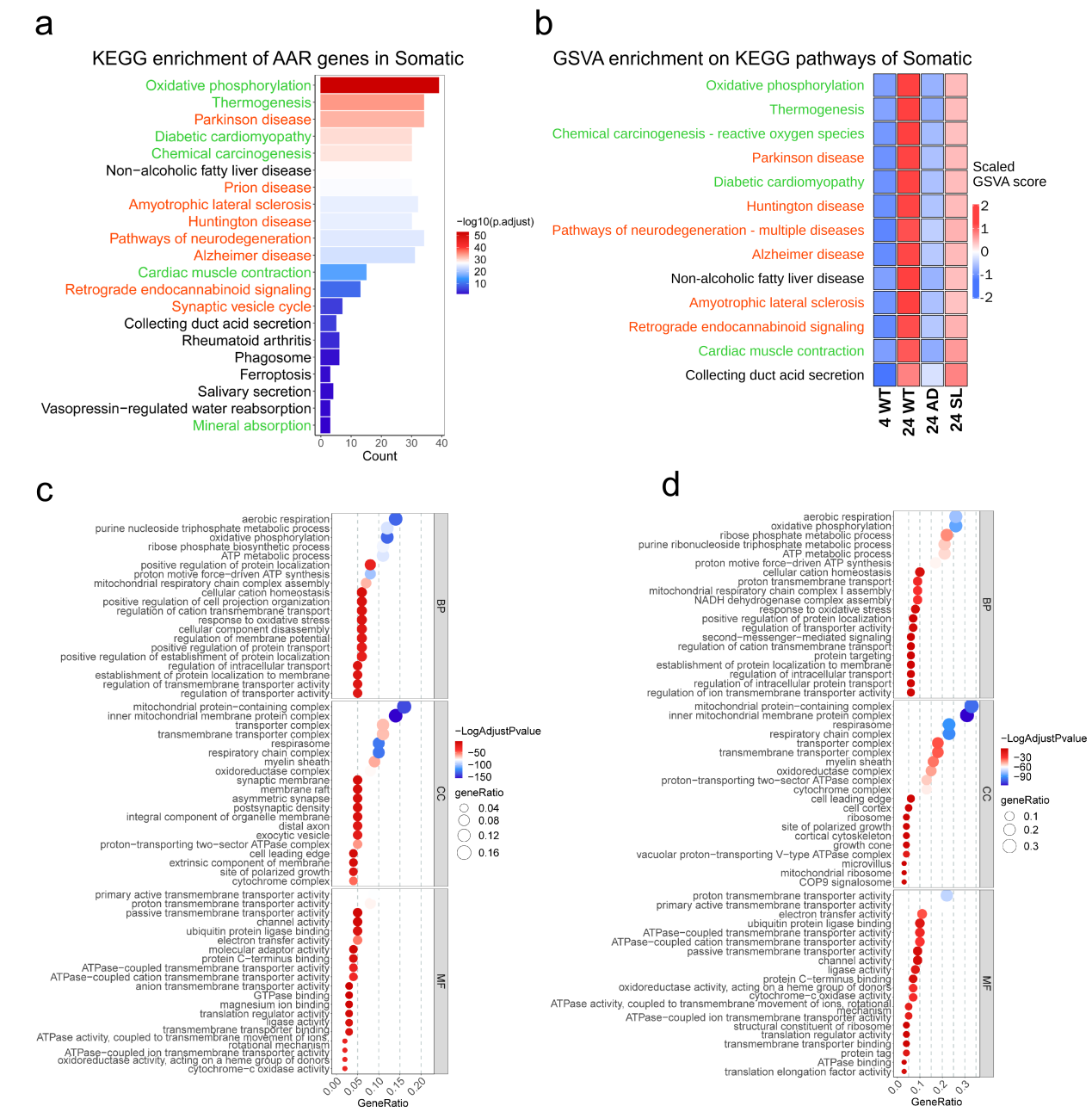


**Figure S5. DEGs with AAR pattern in somatic and dendritic regions of the hippocampus.**

**a-b.** The KEGG analysis on DEGs with AAR expression rule in somatic region of the hippocampus (a), which were further scored by GSVA estimate (b).

**c-d.** The top 20 GO terms in BP, CC, and MF class ranked by gene ratio on DEGs with AAR expression rule in dendritic (total 334 terms) (c) stronger than in somatic regions (total 96 terms) (d) of the hippocampus.


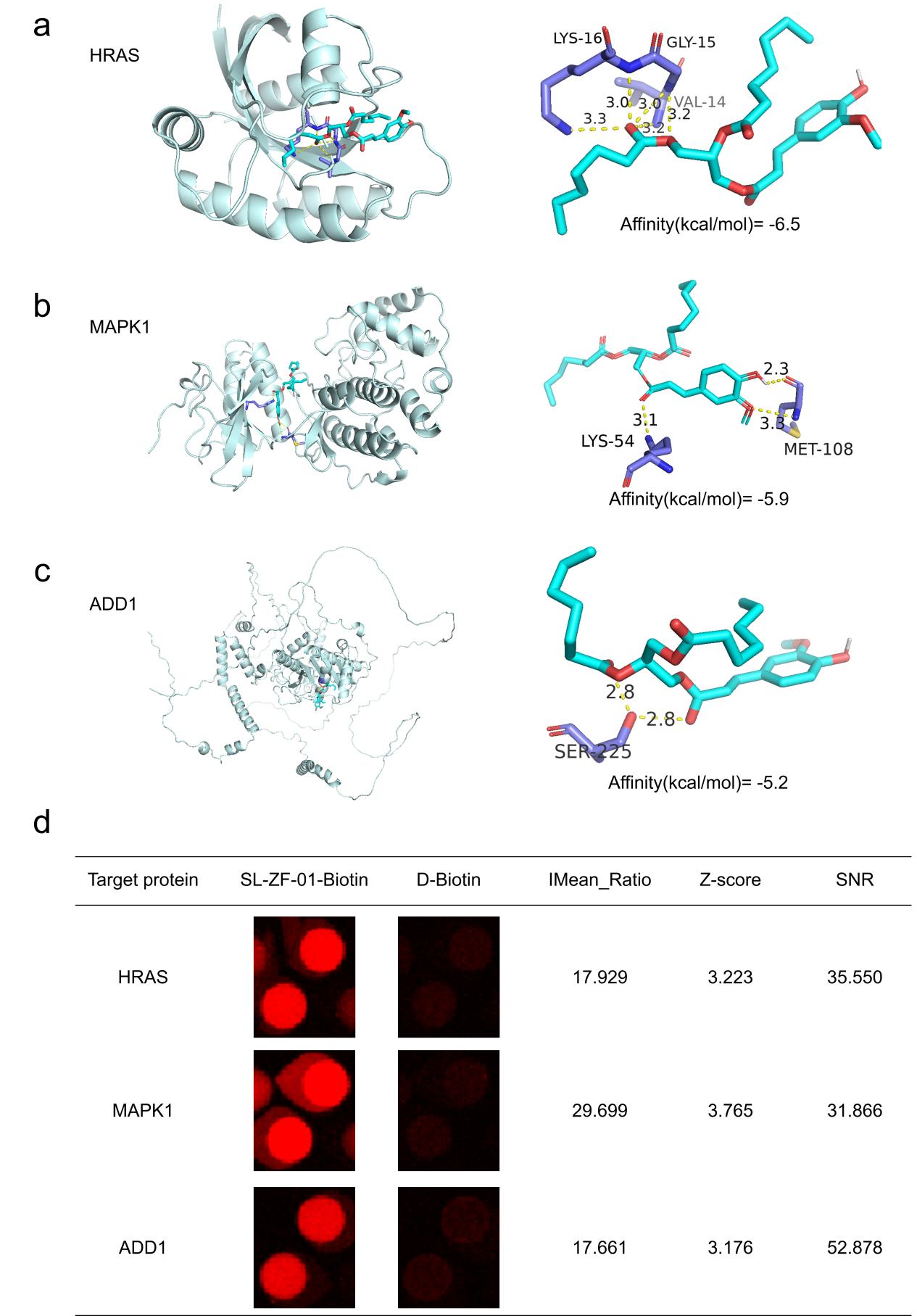


**Figure S6. Identification and validation of SL targets by molecular docking and human proteome microarray**

**a-c.** The interface of the complex between protein and SL-ZF-01 (SL) derived from AutoDockTools. The docking results are visualized by stick model. The complex affinity energies (kcal/mol) are -6.5, -5.9, and -5.2, respectively for panels a, b, and c. The yellow dotted line indicated the hydrogen bond with the length labelled. Amino acid residues with are predicted to interact with SL were also labelled on the stick model.

**d.** Magnified images of the protein array spots probed with SL-ZF-01-biotin (experimental) or D-biotin (control). Proteins with significant binding: HRAS, MAPK1, ADD1. IMean_Ratio: fluorescence intensity ratio of experimental vs. control groups; Z-score, site-corrected signal intensity; SNR, signal-to-noise ratio.


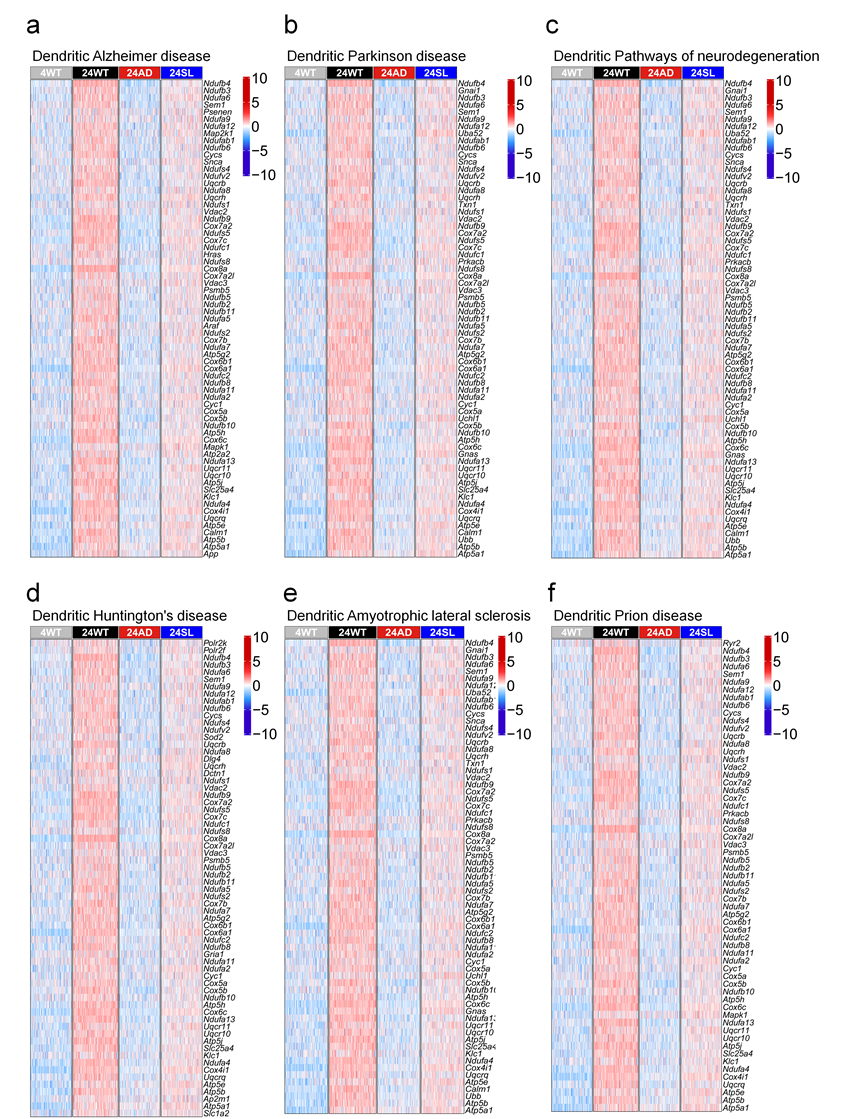


**Figure S7. Neurodegenerative disorders pathways with AAR pattern in hippocampal dendritic region.**

**a-f.** Heatmaps showing the genes with AAR expression pattern in neurodegenerative diseases pathways such as, (a) Alzheimer’s disease, (b) Parkinson’s disease, (c) neurodegeneration, (d) Huntington’s disease, (e) amyotrophic lateral sclerosis, and (f) prion disease.


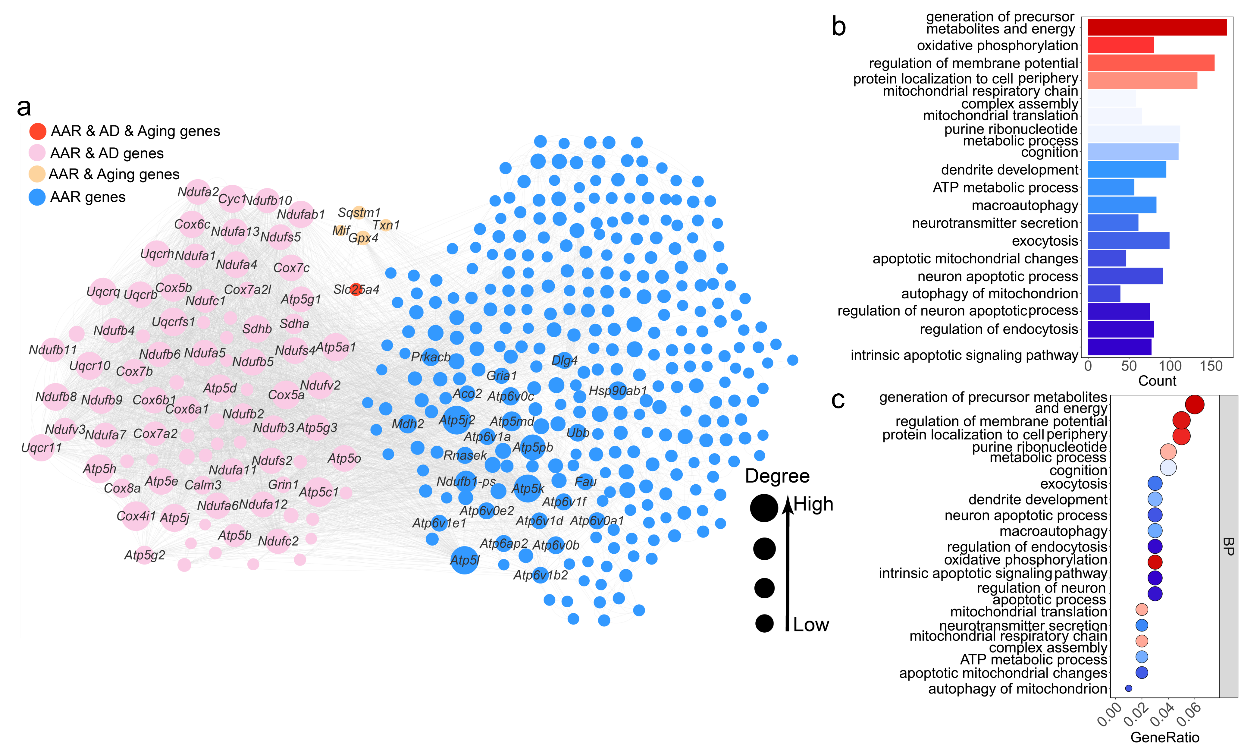


**Figure S8. AAR genes may play an essential role in the progression of both aging and AD**

**a.** PPI network of AAR-AD-Aging, different gene sets were highlighted in distinct colors and the node degree were indicated by node size.

**b.** KEGG enrichment of AAR.

**c.** GO enrichment of AAR.

**Table S1. Summary on spatial transcriptomics sequencing data.**

| Sample Name | Condition | Spots under tissue | Median UMI Counts per Spot | Median Genes per Spot | Total Genes Detected | Serial number | Region |
| --- | --- | --- | --- | --- | --- | --- | --- |
| 4WT | 4 Month WT | 2918 | 20354.5 | 5723 | 21079 | V11F09-071 | A1 |
| 24WT | 24 Month WT | 2540 | 32068.5 | 6997.5 | 21620 | V11F09-071 | B1 |
| 24AD | 24 Month AD | 3012 | 25124 | 6386 | 21667 | V11F09-071 | C1 |
| 24SL | 24 Month SL | 2978 | 28332.5 | 6727.5 | 21691 | V11F09-071 | D1 |

**Table S2. Abbreviation for brain regions**

| **NO.** | **Abbreviation** | **Structure Name** |
| --- | --- | --- |
| 1 | cc | corpus callosum |
| 2 | df | dorsal fornix |
| 3 | dhc | dorsal hippocampal commissure |
| 4 | alv | alveus of the hippocampus |
| 5 | ec | external capsule |
| 6 | fi | fimbria of the hippocampus |
| 7 | st | stria terminalis |
| 8 | ic | internal capsule |
| 9 | cp | cerebral peduncle |
| 10 | opt | optic tract |
| 11 | MePD | medial amygdaloid nucleus, posterodorsal |
| 12 | f | Fornix |
| 13 | PTe | paraterete nucleus |
| 14 | Te | terete hypothalamic nucleus |
| 15 | LHbM | lateral habenular nucleus, medial part |
| 16 | LHbL | lateral habenular nucleus, lateral part |
| 17 | fr | fasciculus retroflexus |
| 18 | PoMn | posteromedian thalamic nucleus |
| 19 | CM | central medial thalamic nucleus |
| 20 | MDM | mediodorsal thalamic nucleus, medial part |
| 21 | MDC | mediodorsal thalamic nucleus, central part |
| 22 | MDL | mediodorsal thalamic nucleus, lateral part |
| 23 | PC | paracentral thalamic nucleus |
| 24 | OPC | oval paracentral thalamic nucleus |
| 25 | VPPC | ventral posterior nucleus of the thalamus, parvicellular x |
| 26 | VM | ventromedial thalamic nucleus x |
| 27 | scp | superior cerebellar peduncle |
| 28 | ml | medial lemniscus |
| 29 | VPL | ventral posterolateral thalamic nucleus |
| 30 | Rt | reticular nucleus (prethalamus) |
| 31 | PrG | pregeniculate nucleus of the prethalamus |
| 32 | ZID | zona incerta, dorsal part |
| 33 | ZIV | zona incerta, ventral part |
| 34 | ns | nigrostriatal bundle |
| 35 | STh | subthalamic nucleus |
| 36 | mt | mamillothalamic tract |
| 37 | DA | dorsal hypothalamic area |
| 38 | DMC | dorsomedial hypothalamic nucleus, compact part |
| 39 | DMV | dorsomedial hypothalamic nucleus, ventral part |
| 40 | VMHDM | ventromedial hypothalamic nucleus, dorsomedial part |
| 41 | VMHC | ventromedial hypothalamic nucleus, central part x |
| 42 | VMHVL | ventromedial hypothalamic nucleus, ventrolateral part |

**Table S2. Abbreviation for brain regions (Continued)**

| **NO.** | **Abbreviation** | **Structure Name** |
| --- | --- | --- |
| 43 | Mtu | medial tuberal nucleus |
| 44 | Po | posterior thalamic nuclear group |
| 45 | LPMR | lateral posterior thalamic nucleus, mediorostral part |
| 46 | LPLR | lateral posterior thalamic nucleus, laterorostral part |
| 47 | DLG | dorsal lateral geniculate nucleus |
| 48 | VPM | ventral posteromedial thalamic nucleus |
| 49 | LaDL | lateral amygdaloid nucleus, dorsolateral |
| 50 | LaVM | lateral amygdaloid nucleus, ventromedial |
| 51 | LaVL | lateral amygdaloid nucleus, ventrolateral part |
| 52 | BLA | basolateral amygdaloid nucleus, anterior |
| 53 | BLP | basolateral amygdaloid nucleus, posterior part |
| 54 | BMP | basomedial amygdaloid nucleus, posterior |
| 55 | BLV | basolateral amygdaloid nucleus, ventral part |
| 56 | Ect | ectorhinal cortex |
| 57 | PRh | perirhinal cortex |
| 58 | DLEnt | dorsolateral entorhinal cortex |
| 59 | MePV | medial amygdaloid nucleus, posteroventral part |
| 60 | PMCo | posteromedial cortical amygdaloid area |
| 61 | AHiAL | amygdalohippocampal area, anterolateral part |
| 62 | PLCo | posterolateral cortical amygdaloid area |
| 63 | RAPir | rostral amygdalopiriform area |
| 64 | STIA | bed nucleus of the stria terminalis, intraamygdaloid division |
| 65 | Cpu | caudate putamen (striatum) |
| 66 | ASt | amygdalostriatal transition area |
| 67 | CeL | central amygdaloid nucleus, lateral part |

**Table S3. DEGs with 8 combinations of expression pattern.**

| Eight combi-  nations  Brain  region | ↑↓↑ | ↑↑↓ | ↑↓↓ | ↑↑↑ | ↓↑↑ | ↓↑↓ | ↓↓↑ | ↓↓↓ | Spots number |
| --- | --- | --- | --- | --- | --- | --- | --- | --- | --- |
| Layer1/2 | 1749 | 19 | 4 | 3 | 1 | 1 | 1 | 0 | 1514 |
| Layer3/4 | 366 | 3 | 1 | 0 | 0 | 0 | 0 | 0 | 669 |
| Layer5 | 412 | 4 | 6 | 3 | 0 | 0 | 0 | 0 | 641 |
| Layer6 | 477 | 9 | 1 | 3 | 0 | 0 | 0 | 0 | 752 |
| Somatic | 114 | 1 | 1 | 0 | 0 | 0 | 0 | 0 | 347 |
| Dendritic | 457 | 7 | 3 | 1 | 0 | 0 | 0 | 0 | 874 |
| C0 | 1330 | 20 | 9 | 8 | 0 | 1 | 0 | 1 | 1547 |
| C1 | 1663 | 9 | 60 | 10 | 1 | 1 | 0 | 0 | 2506 |
| C2 | 284 | 24 | 2 | 3 | 0 | 0 | 0 | 0 | 962 |
| C3 | 329 | 4 | 3 | 1 | 1 | 0 | 0 | 0 | 778 |
| C4 | 86 | 1 | 0 | 0 | 0 | 0 | 0 | 0 | 425 |
| C5 | 313 | 18 | 1 | 0 | 0 | 0 | 1 | 0 | 422 |

The three arrows in each blanket in the first row respectively indicated the three pairwise comparisons: 4WT vs 24WT, 24WT vs 24AD, and 24AD vs 24SL. Up arrows = increased expression; Down arrows = decreased expression. Thus, there are 8 possible combinations of gene expression pattern (2×2×2). The first type with up-down-up expression pattern had dominant numbers of DEGs in 14 brain regions, termed as Aging-AD-Rescue (AAR) expression rule. DEGs, differentially expressed genes.
