# Supplementary material for: Modifying Glucose Metabolism Reverses Memory Defects of Alzheimer's Disease Model at Late Stages
Source: Adv Sci (Weinh). 2025 Dec 8;13(11):e06695. doi: 10.1002/advs.202506695 (PMC12931168; doi:10.1002/advs.202506695)
Supplement: Supplementary file 2 — Supporting Information [file ADVS-13-e06695-s002.docx]

**Table S4. 3065 AAR genes across all brain regions**

| **ID** | **AAR genes** | **Region** |
| --- | --- | --- |
| 1 | ***0610010K14Rik*** | C1,C0 |
| 2 | ***0610012G03Rik*** | Layer6,C1,C3,Layer1/2,C0,C5,Layer5 |
| 3 | ***1110004F10Rik*** | Layer6,C3,Layer1/2 |
| 4 | ***1110008P14Rik*** | Dendritic,Layer6,C1,C3,Layer1/2,C0,C5 |
| 5 | ***1110032A03Rik*** | C1,C0 |
| 6 | ***1110038B12Rik*** | C0 |
| 7 | ***1110051M20Rik*** | C1,Layer1/2,C0 |
| 8 | ***1110065P20Rik*** | C1,Layer1/2,C0,C5,Layer5 |
| 9 | ***1190007I07Rik*** | Layer1/2 |
| 10 | ***1500004A13Rik*** | C1 |
| 11 | ***1500011B03Rik*** | Dendritic,Layer6,C1,C3,Layer1/2,C0,Layer5 |
| 12 | ***1600020E01Rik*** | Layer1/2 |
| 13 | ***1810026B05Rik*** | Layer1/2 |
| 14 | ***1810037I17Rik*** | Dendritic,Layer6,C1,Layer1/2,C0,Layer5 |
| 15 | ***1810058I24Rik*** | C1,C0 |
| 16 | ***2010204K13Rik*** | Layer1/2 |
| 17 | ***2010300C02Rik*** | C1,C3,Layer1/2,C5,Layer3/4 |
| 18 | ***2210016L21Rik*** | C1,Layer1/2,C0 |
| 19 | ***2310009A05Rik*** | Layer1/2 |
| 20 | ***2310009B15Rik*** | C1,C0 |
| 21 | ***2310061I04Rik*** | C1 |
| 22 | ***2410002F23Rik*** | Layer1/2,C0 |
| 23 | ***2410006H16Rik*** | Dendritic,Layer6,C1,C0 |
| 24 | ***2610001J05Rik*** | Layer1/2 |
| 25 | ***2610301B20Rik*** | Layer1/2 |
| 26 | ***2810013P06Rik*** | Layer1/2 |
| 27 | ***2810455O05Rik*** | Layer1/2 |
| 28 | ***2900076A07Rik*** | C3 |
| 29 | ***2900093K20Rik*** | Layer1/2 |
| 30 | ***2900097C17Rik*** | C1 |
| 31 | ***3110039M20Rik*** | Layer6 |
| 32 | ***4833439L19Rik*** | C1 |
| 33 | ***4933431E20Rik*** | C0 |
| 34 | ***4933434E20Rik*** | C1 |
| 35 | ***5031425E22Rik*** | Layer1/2 |
| 36 | ***5430416N02Rik*** | Layer6,Layer1/2 |
| 37 | ***5730455P16Rik*** | C0 |
| 38 | ***6430548M08Rik*** | C1,C2 |
| 39 | ***9330151L19Rik*** | Layer1/2 |
| 40 | ***9330159F19Rik*** | C0 |
| 41 | ***A230057D06Rik*** | Layer1/2 |
| 42 | ***A2ml1*** | Layer1/2,C0 |
| 43 | ***A830018L16Rik*** | Dendritic,Layer1/2,C0 |
| 44 | ***A830036E02Rik*** | Layer1/2 |
| 45 | ***A830082K12Rik*** | Layer1/2 |
| 46 | ***Aak1*** | C1,Layer3/4 |
| 47 | ***Aamdc*** | Layer1/2,C0 |
| 48 | ***Aar2*** | C1 |
| 49 | ***Aars*** | C1,Layer1/2,C0 |
| 50 | ***Aarsd1*** | C1 |
| 51 | ***Abat*** | C0 |
| 52 | ***Abca2*** | C1,Layer1/2,C0 |
| 53 | ***Abca3*** | C1,C0 |
| 54 | ***Abcb1a*** | Layer1/2 |
| 55 | ***Abcc5*** | Layer1/2 |
| 56 | ***Abcc8*** | Layer1/2,C2 |
| 57 | ***Abcd3*** | C1 |
| 58 | ***Abce1*** | C1,C0 |
| 59 | ***Abcf2*** | C1,Layer1/2,C0 |
| 60 | ***Abcg4*** | C1,Layer1/2,Layer3/4 |
| 61 | ***Abhd12*** | Dendritic,Layer6,C1,C3,Layer1/2,C0 |
| 62 | ***Abhd16a*** | Layer1/2 |
| 63 | ***Abhd17a*** | C1,Layer1/2,C0 |
| 64 | ***Abhd6*** | Layer1/2,C0 |
| 65 | ***Abhd8*** | Dendritic,Layer6,C1,Layer1/2,C2,C0,Layer5,Layer3/4 |
| 66 | ***Abi1*** | Dendritic,C1,Layer1/2 |
| 67 | ***Abi2*** | Layer1/2,Layer5 |
| 68 | ***Ablim2*** | C1,Layer1/2 |
| 69 | ***Abr*** | C1,C3,Layer1/2,C2,C0,C5,Layer5,Layer3/4 |
| 70 | ***Abraxas2*** | Layer1/2 |
| 71 | ***Acadl*** | C0 |
| 72 | ***Acap2*** | Layer1/2 |
| 73 | ***Acat1*** | C1 |
| 74 | ***Acbd5*** | C0 |
| 75 | ***Acbd6*** | C1 |
| 76 | ***Acly*** | C1 |
| 77 | ***Aco2*** | Dendritic,Layer6,C1,C3,Layer1/2,C0,C5 |
| 78 | ***Acot13*** | Dendritic,Layer6,C1,Layer1/2,C0,C5,Layer5 |
| 79 | ***Acot7*** | C1 |
| 80 | ***Acox1*** | C1,Layer1/2,C0 |
| 81 | ***Acp1*** | C1,C0 |
| 82 | ***Acsbg1*** | C0 |
| 83 | ***Acsl3*** | Dendritic,C1,Layer1/2,C2,C0 |
| 84 | ***Acsl5*** | Layer1/2 |
| 85 | ***Acsl6*** | Dendritic,C1 |
| 86 | ***Actg1*** | Dendritic,C1,Layer1/2,Layer5,Layer3/4 |
| 87 | ***Actl6b*** | Layer1/2,C5,Layer5 |
| 88 | ***Actr10*** | C1 |
| 89 | ***Actr1b*** | C1,Layer1/2,C2,Layer3/4 |
| 90 | ***Actr2*** | C1,Layer1/2,C2,C5 |
| 91 | ***Actr3*** | C1,C5 |
| 92 | ***Actr3b*** | C1,Layer1/2,Layer3/4 |
| 93 | ***Acvr1*** | Layer1/2 |
| 94 | ***Acyp1*** | C1 |
| 95 | ***Acyp2*** | C1,Layer1/2,C0 |
| 96 | ***Adam11*** | Layer1/2 |
| 97 | ***Adap1*** | C1 |
| 98 | ***Adarb1*** | C1,C0 |
| 99 | ***Adarb2*** | Layer1/2 |
| 100 | ***Adcy1*** | Layer6,C1,Layer1/2,Layer5 |
| 101 | ***Adcy3*** | Layer1/2,C0 |
| 102 | ***Add1*** | Layer1/2,C0 |
| 103 | ***Adgra1*** | C0 |
| 104 | ***Adgrb1*** | C1,Layer1/2,C2,C0,C5 |
| 105 | ***Adgrb2*** | C1,Layer1/2 |
| 106 | ***Adh5*** | C0 |
| 107 | ***Ado*** | C1 |
| 108 | ***Adora1*** | C2 |
| 109 | ***Adprh*** | C1,C0 |
| 110 | ***Adra1b*** | Layer1/2 |
| 111 | ***Adrb3*** | Layer1/2 |
| 112 | ***Adsl*** | Layer1/2 |
| 113 | ***Adss*** | C2 |
| 114 | ***Aff3*** | Layer1/2 |
| 115 | ***Aff4*** | Layer1/2 |
| 116 | ***Afg3l2*** | C1,Layer1/2,C2,C0 |
| 117 | ***Agap3*** | C1,Layer1/2,Layer3/4 |
| 118 | ***Agk*** | Layer1/2 |
| 119 | ***Agpat3*** | C1 |
| 120 | ***Agrn*** | Layer1/2 |
| 121 | ***Agtpbp1*** | Dendritic,C1,Layer1/2,C2,C0 |
| 122 | ***Ahcyl1*** | C0 |
| 123 | ***Ahcyl2*** | Dendritic |
| 124 | ***Ahsa1*** | C0 |
| 125 | ***AI413582*** | Dendritic,Layer6,C1,Layer1/2,C0,C5,Layer5,Layer3/4 |
| 126 | ***AI593442*** | C1 |
| 127 | ***Aifm3*** | C1,Layer1/2,C0 |
| 128 | ***Aig1*** | C0 |
| 129 | ***Aimp1*** | C1,Layer1/2,C0,Layer5 |
| 130 | ***Aip*** | C1,C2,C0 |
| 131 | ***Ajap1*** | C1,Layer1/2,C0 |
| 132 | ***Ak1*** | C1,C2,C0 |
| 133 | ***Ak5*** | Dendritic,Layer1/2,C0 |
| 134 | ***Akap11*** | C0 |
| 135 | ***Akap6*** | C1,C0 |
| 136 | ***Akirin2*** | C1 |
| 137 | ***Akr1a1*** | Dendritic,Layer6,C1,Layer1/2,C5 |
| 138 | ***Alas1*** | C1,C0 |
| 139 | ***Aldh3a2*** | C0 |
| 140 | ***Aldh6a1*** | C0 |
| 141 | ***Aldoa*** | Dendritic,Somatic,Layer6,C1,Layer1/2,C2,C0,C5,Layer5,Layer3/4 |
| 142 | ***Alg1*** | Layer1/2 |
| 143 | ***Alg11*** | Layer1/2 |
| 144 | ***Alg13*** | Layer1/2 |
| 145 | ***Alg9*** | Layer1/2 |
| 146 | ***Alkbh6*** | C1,Layer1/2,C0,C5 |
| 147 | ***Amd1*** | Layer1/2 |
| 148 | ***Amfr*** | Layer1/2 |
| 149 | ***Amph*** | C1,Layer1/2,C0 |
| 150 | ***Anapc11*** | C1 |
| 151 | ***Anapc13*** | Dendritic,C1,C0 |
| 152 | ***Anapc16*** | C1,Layer1/2 |
| 153 | ***Anapc5*** | C3,C0 |
| 154 | ***Ank2*** | C1,Layer1/2,Layer3/4 |
| 155 | ***Ank3*** | Dendritic,C0 |
| 156 | ***Ankle2*** | Layer1/2 |
| 157 | ***Ankrd24*** | Layer1/2,Layer5 |
| 158 | ***Ankrd28*** | Layer1/2 |
| 159 | ***Ankrd33b*** | Layer1/2 |
| 160 | ***Ankrd35*** | Layer1/2 |
| 161 | ***Ankrd45*** | Layer1/2 |
| 162 | ***Ankrd46*** | Dendritic,C1,Layer1/2,C0,Layer3/4 |
| 163 | ***Ankrd6*** | Layer1/2 |
| 164 | ***Anks1b*** | C3,Layer1/2,C2,Layer5 |
| 165 | ***Ano3*** | C0 |
| 166 | ***Anp32e*** | C0 |
| 167 | ***Anxa7*** | C1 |
| 168 | ***Ap1b1*** | C1 |
| 169 | ***Ap1m1*** | C0 |
| 170 | ***Ap1s1*** | Dendritic,C1,Layer1/2,C0 |
| 171 | ***Ap2a1*** | C1,Layer1/2,C2 |
| 172 | ***Ap2a2*** | C1 |
| 173 | ***Ap2m1*** | Dendritic,C1,Layer1/2,C0 |
| 174 | ***Ap2s1*** | C1,Layer1/2,C0 |
| 175 | ***Ap3b2*** | C1 |
| 176 | ***Ap4b1*** | Layer1/2 |
| 177 | ***Ap4s1*** | C1,Layer1/2 |
| 178 | ***Ap5s1*** | C1 |
| 179 | ***Apba2*** | C0 |
| 180 | ***Apbb1*** | C1,Layer1/2,C5 |
| 181 | ***Apbb3*** | C0 |
| 182 | ***Apc*** | C0 |
| 183 | ***Apip*** | Layer1/2 |
| 184 | ***Aplp1*** | Dendritic,Somatic,C1,C3,Layer1/2,C0,C5,Layer5,Layer3/4 |
| 185 | ***Aplp2*** | C1,C2,C0 |
| 186 | ***Apoo*** | C1,Layer1/2,C5 |
| 187 | ***App*** | Dendritic,Layer6,C1,Layer1/2,C2,C4,C0,C5,Layer5,Layer3/4 |
| 188 | ***Appl1*** | C1 |
| 189 | ***Araf*** | Dendritic,Layer6,C1 |
| 190 | ***Arel1*** | C1,C5 |
| 191 | ***Arf1*** | Layer6,C1,C3,Layer1/2,C2,C0,C5,Layer5,Layer3/4 |
| 192 | ***Arf2*** | Layer1/2 |
| 193 | ***Arf3*** | C1,C3,Layer1/2,C2 |
| 194 | ***Arf4*** | Layer1/2 |
| 195 | ***Arf5*** | Dendritic,Layer6,C1,C3,Layer1/2,C2,C0,C5,Layer5 |
| 196 | ***Arfgap1*** | Layer1/2 |
| 197 | ***Arfgef1*** | C1 |
| 198 | ***Arfgef2*** | Layer1/2 |
| 199 | ***Arfgef3*** | C1,Layer1/2 |
| 200 | ***Arfip2*** | C1 |
| 201 | ***Arhgap1*** | Layer1/2,C0 |
| 202 | ***Arhgap21*** | Dendritic,C1,C0 |
| 203 | ***Arhgap26*** | C0 |
| 204 | ***Arhgap27*** | Layer1/2 |
| 205 | ***Arhgap31*** | Layer1/2 |
| 206 | ***Arhgap35*** | C1 |
| 207 | ***Arhgap39*** | C0 |
| 208 | ***Arhgap44*** | C1 |
| 209 | ***Arhgap5*** | C0 |
| 210 | ***Arhgdig*** | C1 |
| 211 | ***Arhgef25*** | Layer1/2 |
| 212 | ***Arhgef4*** | Dendritic,C0 |
| 213 | ***Arhgef40*** | Layer1/2 |
| 214 | ***Arhgef9*** | Layer1/2 |
| 215 | ***Arl2*** | C1 |
| 216 | ***Arl3*** | C1,C0 |
| 217 | ***Arl4d*** | C1,C0 |
| 218 | ***Arl6*** | Layer1/2 |
| 219 | ***Arl6ip1*** | C1 |
| 220 | ***Arl6ip4*** | C1 |
| 221 | ***Arl6ip5*** | C1,Layer1/2 |
| 222 | ***Arl8a*** | C1,C0 |
| 223 | ***Arl8b*** | C1,C3,Layer1/2 |
| 224 | ***Armc1*** | C1,Layer1/2,C0 |
| 225 | ***Armcx1*** | C1 |
| 226 | ***Arpc1a*** | C1,Layer1/2,C0 |
| 227 | ***Arpc2*** | Dendritic,Layer6,C1,C3,Layer1/2,Layer5,Layer3/4 |
| 228 | ***Arpc3*** | Layer6,C1,C3,Layer1/2,C0 |
| 229 | ***Arpc4*** | C1,Layer1/2,C0,Layer3/4 |
| 230 | ***Arpc5*** | Layer1/2 |
| 231 | ***Arpc5l*** | C1,Layer1/2,C0,C5 |
| 232 | ***Arpp21*** | Layer1/2 |
| 233 | ***Arx*** | Layer1/2 |
| 234 | ***Asap1*** | C1 |
| 235 | ***Asb13*** | C1,C0 |
| 236 | ***Asna1*** | C1 |
| 237 | ***Asns*** | C1 |
| 238 | ***Asxl2*** | Layer1/2 |
| 239 | ***Atad1*** | C3,C0 |
| 240 | ***Atf2*** | C0 |
| 241 | ***Atf4*** | C1,C2 |
| 242 | ***Atg10*** | Layer1/2 |
| 243 | ***Atg12*** | C1 |
| 244 | ***Atg14*** | Layer1/2 |
| 245 | ***Atg16l2*** | Layer1/2 |
| 246 | ***Atg3*** | C1,Layer1/2,C0 |
| 247 | ***Atg9a*** | Layer1/2 |
| 248 | ***Atl1*** | C0 |
| 249 | ***Atox1*** | C1,C0 |
| 250 | ***Atp13a3*** | Layer1/2 |
| 251 | ***Atp1a1*** | C3,C5 |
| 252 | ***Atp1a3*** | C2 |
| 253 | ***Atp1b1*** | Dendritic,Somatic,Layer6,C1,C3,Layer1/2,C2,C5,Layer5,Layer3/4 |
| 254 | ***Atp2a2*** | Dendritic,C1,C3,Layer1/2,C0,C5,Layer3/4 |
| 255 | ***Atp2b1*** | C3,Layer1/2,C5,Layer5 |
| 256 | ***Atp2b2*** | C1,Layer1/2,C0,C5 |
| 257 | ***Atp5a1*** | Dendritic,Somatic,Layer6,C1,C3,Layer1/2,C2,C0,C5,Layer5,Layer3/4 |
| 258 | ***Atp5b*** | Dendritic,Somatic,Layer6,C1,C3,Layer1/2,C0,C5,Layer5,Layer3/4 |
| 259 | ***Atp5c1*** | Layer6,C1,C3,Layer1/2,C0,Layer5 |
| 260 | ***Atp5d*** | Layer6,C1,Layer1/2,C0,C5 |
| 261 | ***Atp5e*** | Dendritic,Somatic,Layer6,C1,C3,Layer1/2,C0,C5 |
| 262 | ***Atp5f1*** | Layer6,C1,C0 |
| 263 | ***Atp5g1*** | Layer6,C1,Layer1/2,C5 |
| 264 | ***Atp5g2*** | Dendritic,Layer6,C1,Layer1/2,C2,C0,C5,Layer5 |
| 265 | ***Atp5g3*** | C1,C5 |
| 266 | ***Atp5h*** | Dendritic,Somatic,Layer6,C1,C3,Layer1/2,C2,C4,C0,C5,Layer5,Layer3/4 |
| 267 | ***Atp5j*** | Dendritic,Layer6,C1,C3,Layer1/2,C4,C0,C5,Layer5,Layer3/4 |
| 268 | ***Atp5j2*** | Dendritic,Somatic,Layer6,C1,C3,Layer1/2,C0,C5,Layer5,Layer3/4 |
| 269 | ***Atp5k*** | Dendritic,Somatic,Layer6,C1,Layer1/2,C0,C5,Layer3/4 |
| 270 | ***Atp5l*** | Dendritic,Somatic,Layer6,C1,C3,Layer1/2,C2,C4,C0,C5,Layer5,Layer3/4 |
| 271 | ***Atp5md*** | Dendritic,Somatic,Layer6,C1,C3,Layer1/2,C2,C0,C5,Layer5,Layer3/4 |
| 272 | ***Atp5mpl*** | Dendritic,Somatic,Layer6,C1,C3,Layer1/2,C0,C5,Layer5,Layer3/4 |
| 273 | ***Atp5o*** | C1,C3,Layer1/2 |
| 274 | ***Atp6ap1*** | C1,Layer1/2,Layer3/4 |
| 275 | ***Atp6ap1l*** | Layer1/2 |
| 276 | ***Atp6ap2*** | C1 |
| 277 | ***Atp6v0a1*** | C1 |
| 278 | ***Atp6v0b*** | Dendritic,Somatic,Layer6,C1,Layer1/2,C0,Layer5,Layer3/4 |
| 279 | ***Atp6v0c*** | Dendritic,Somatic,Layer6,C1,Layer1/2,C0,C5,Layer5,Layer3/4 |
| 280 | ***Atp6v0d1*** | Dendritic,C1,Layer1/2 |
| 281 | ***Atp6v0e*** | C0 |
| 282 | ***Atp6v0e2*** | Dendritic,Somatic,C1,Layer1/2,C2,C0,C5,Layer3/4 |
| 283 | ***Atp6v1a*** | Dendritic,Somatic,Layer6,C1,Layer1/2,C4,C0,C5,Layer5,Layer3/4 |
| 284 | ***Atp6v1b2*** | Dendritic,Somatic,C1,Layer1/2,C2,C0,Layer5,Layer3/4 |
| 285 | ***Atp6v1c1*** | Dendritic,C1,Layer1/2,C4,C0 |
| 286 | ***Atp6v1d*** | Dendritic,Layer6,C1,Layer1/2,C2,C0,C5,Layer5 |
| 287 | ***Atp6v1e1*** | Dendritic,Somatic,Layer6,C1,C3,Layer1/2,C2,C0,C5,Layer5,Layer3/4 |
| 288 | ***Atp6v1f*** | Dendritic,Layer6,C1,Layer1/2,C4,C0,C5,Layer5,Layer3/4 |
| 289 | ***Atp6v1g1*** | Dendritic,Layer6,C1,Layer1/2,Layer5 |
| 290 | ***Atp6v1g2*** | C1,Layer1/2 |
| 291 | ***Atp6v1h*** | C1 |
| 292 | ***Atp8a1*** | C3,Layer1/2 |
| 293 | ***Atp9b*** | Layer1/2 |
| 294 | ***Atpaf1*** | C1,Layer1/2 |
| 295 | ***Atpif1*** | Dendritic,Somatic,Layer6,C1,C3,Layer1/2,C2,C4,C0,C5,Layer5,Layer3/4 |
| 296 | ***Atraid*** | C0 |
| 297 | ***Atrn*** | Layer1/2 |
| 298 | ***Atrnl1*** | C1,Layer1/2,C0 |
| 299 | ***Atxn1*** | Layer1/2 |
| 300 | ***Atxn10*** | C1 |
| 301 | ***Atxn2*** | C1 |
| 302 | ***Atxn2l*** | Layer1/2 |
| 303 | ***Atxn7l2*** | Layer1/2 |
| 304 | ***Atxn7l3*** | C1,C2 |
| 305 | ***AU040320*** | Layer1/2 |
| 306 | ***Auh*** | Dendritic,Layer6,C1,C0 |
| 307 | ***Aurkaip1*** | C1,Layer1/2 |
| 308 | ***Auts2*** | C0 |
| 309 | ***AV356131*** | Dendritic |
| 310 | ***Azin1*** | Layer1/2,C2,C0 |
| 311 | ***B230118H07Rik*** | C1,C0 |
| 312 | ***B230217C12Rik*** | C1,C0 |
| 313 | ***B230219D22Rik*** | Layer1/2,Layer3/4 |
| 314 | ***B230334C09Rik*** | Layer1/2,C0 |
| 315 | ***B3galnt1*** | C1,C0 |
| 316 | ***B3gat1*** | C1,C3 |
| 317 | ***B3gat3*** | C1,Layer1/2,C0 |
| 318 | ***B3gnt2*** | Layer3/4 |
| 319 | ***B4galt2*** | C0 |
| 320 | ***B930059L03Rik*** | Layer1/2 |
| 321 | ***B9d1*** | Layer1/2 |
| 322 | ***Baalc*** | Dendritic,Layer1/2,C0 |
| 323 | ***Babam1*** | C1 |
| 324 | ***Babam2*** | C1 |
| 325 | ***Bad*** | C1 |
| 326 | ***Bag1*** | C1,C0 |
| 327 | ***Bag4*** | Layer6 |
| 328 | ***Bag6*** | C1 |
| 329 | ***Bahd1*** | Layer1/2 |
| 330 | ***Baiap2*** | C1,C3,Layer1/2,Layer3/4 |
| 331 | ***Banf1*** | C1,Layer1/2 |
| 332 | ***Banp*** | Layer1/2 |
| 333 | ***Bap1*** | C1,Layer1/2,C0 |
| 334 | ***Basp1*** | Dendritic,Layer6,Layer1/2,C4,C0,Layer3/4 |
| 335 | ***Bax*** | C1,Layer1/2 |
| 336 | ***Bbip1*** | Layer1/2,C0 |
| 337 | ***Bbs4*** | Dendritic |
| 338 | ***BC003965*** | C0 |
| 339 | ***BC004004*** | C1,C0 |
| 340 | ***BC005537*** | C1,C0 |
| 341 | ***BC005624*** | C1,C0 |
| 342 | ***BC029722*** | C1,C0,Layer5 |
| 343 | ***BC031181*** | Dendritic,Layer6,C1,C3,Layer1/2,C2,C0,C5,Layer5,Layer3/4 |
| 344 | ***Bc1*** | Layer6,C1,C3,C0,Layer5,Layer3/4 |
| 345 | ***Bcap29*** | Layer6 |
| 346 | ***Bcap31*** | C1 |
| 347 | ***Bcas2*** | Layer1/2,C0,Layer5 |
| 348 | ***Bcl11a*** | Layer1/2,C0,Layer5 |
| 349 | ***Bcl2l1*** | C1 |
| 350 | ***Bcl6*** | Layer1/2 |
| 351 | ***Bcl7a*** | C0 |
| 352 | ***Bclaf1*** | C1 |
| 353 | ***Becn1*** | C1,Layer1/2 |
| 354 | ***Bend6*** | C2,C0 |
| 355 | ***Bet1l*** | Layer1/2 |
| 356 | ***Bex1*** | C0,Layer5 |
| 357 | ***Bex2*** | Dendritic,Layer6,C1,C3,Layer1/2,C2,C0,Layer5,Layer3/4 |
| 358 | ***Bex3*** | C1 |
| 359 | ***Bhlhe22*** | C3,Layer1/2,C0,Layer3/4 |
| 360 | ***Bloc1s1*** | C1,C0 |
| 361 | ***Bloc1s2*** | Layer1/2 |
| 362 | ***Bloc1s4*** | Layer1/2 |
| 363 | ***Bmerb1*** | C1 |
| 364 | ***Bmp1*** | Layer1/2 |
| 365 | ***Bmpr2*** | C1,Layer1/2 |
| 366 | ***Bmyc*** | C1 |
| 367 | ***Bnip3*** | Dendritic,C1,Layer1/2,C0 |
| 368 | ***Bnip3l*** | C1 |
| 369 | ***Bod1*** | C1 |
| 370 | ***Bok*** | C0 |
| 371 | ***Bola1*** | C0 |
| 372 | ***Bola2*** | C1,C0 |
| 373 | ***Bola3*** | Dendritic,Layer6,C1,Layer1/2,C0 |
| 374 | ***Borcs5*** | C0 |
| 375 | ***Borcs6*** | C0 |
| 376 | ***Borcs8*** | Layer1/2 |
| 377 | ***Bphl*** | Dendritic,C0 |
| 378 | ***Brd7*** | C2 |
| 379 | ***Brd9*** | C0 |
| 380 | ***Bri3*** | C1 |
| 381 | ***Bri3bp*** | C1,Layer1/2,Layer5 |
| 382 | ***Brinp1*** | C1,Layer1/2 |
| 383 | ***Brinp2*** | C5 |
| 384 | ***Brk1*** | Dendritic,Layer6,C1,Layer1/2,C0,C5 |
| 385 | ***Brms1l*** | C1,C0 |
| 386 | ***Brpf1*** | Layer1/2 |
| 387 | ***Brsk2*** | C0 |
| 388 | ***Bscl2*** | C1 |
| 389 | ***Bsg*** | C1 |
| 390 | ***Bsn*** | Dendritic,C1,Layer1/2,C0 |
| 391 | ***Btbd2*** | C1 |
| 392 | ***Btbd3*** | C1,Layer1/2,C0 |
| 393 | ***Btbd8*** | Layer1/2 |
| 394 | ***Btf3*** | Dendritic,Layer6,C1,Layer1/2,C0,Layer5 |
| 395 | ***Btf3l4*** | Layer1/2,C0 |
| 396 | ***Btg3*** | Layer1/2 |
| 397 | ***Btrc*** | Layer1/2 |
| 398 | ***Bub3*** | C1,C0 |
| 399 | ***Bud31*** | C1,Layer1/2 |
| 400 | ***Bzw1*** | C1 |
| 401 | ***Bzw2*** | Layer1/2 |
| 402 | ***C1d*** | C0 |
| 403 | ***C1galt1c1*** | Layer1/2 |
| 404 | ***C1qbp*** | C1 |
| 405 | ***C1qtnf4*** | C1 |
| 406 | ***C2cd2l*** | Layer1/2 |
| 407 | ***C2cd5*** | Layer1/2 |
| 408 | ***Cab39*** | C1,C0 |
| 409 | ***Cabin1*** | Layer1/2 |
| 410 | ***Cabp1*** | Layer1/2,C0 |
| 411 | ***Cabp7*** | C0 |
| 412 | ***Cacna1a*** | C1,C0 |
| 413 | ***Cacna1c*** | Layer1/2 |
| 414 | ***Cacna1e*** | C1,Layer1/2 |
| 415 | ***Cacna2d3*** | Layer1/2 |
| 416 | ***Cacnb3*** | Layer1/2 |
| 417 | ***Cacnb4*** | C1 |
| 418 | ***Cacng3*** | Layer1/2 |
| 419 | ***Cadm2*** | C1 |
| 420 | ***Cadps*** | Dendritic,Layer6,C1,C3,Layer1/2,C0,C5,Layer3/4 |
| 421 | ***Cadps2*** | C0 |
| 422 | ***Calb1*** | C3,Layer1/2,C2,C5 |
| 423 | ***Calm1*** | Dendritic,Somatic,Layer6,C1,C3,Layer1/2,C2,C0,C5,Layer5,Layer3/4 |
| 424 | ***Calm2*** | Layer6,C1,C3,Layer1/2,C5,Layer5,Layer3/4 |
| 425 | ***Calm3*** | C1,C3,Layer1/2 |
| 426 | ***Caly*** | Layer6,Layer1/2,C0,Layer5 |
| 427 | ***Camk1d*** | C1,Layer1/2 |
| 428 | ***Camk1g*** | Layer1/2 |
| 429 | ***Camk2a*** | Layer1/2,C5 |
| 430 | ***Camk2b*** | C1,Layer1/2,C2 |
| 431 | ***Camk2d*** | Layer1/2,Layer3/4 |
| 432 | ***Camk2g*** | Layer1/2 |
| 433 | ***Camk2n1*** | Dendritic,C1,Layer1/2 |
| 434 | ***Camk2n2*** | C3 |
| 435 | ***Camkk1*** | Layer1/2,C0 |
| 436 | ***Camta1*** | Dendritic,C1,C0 |
| 437 | ***Camta2*** | C1,Layer1/2,C5 |
| 438 | ***Cand1*** | C1 |
| 439 | ***Cap2*** | C1,Layer1/2,C5,Layer3/4 |
| 440 | ***Capn11*** | C1,Layer1/2 |
| 441 | ***Capza2*** | Dendritic,Somatic,Layer6,C1,Layer1/2,C0,C5,Layer3/4 |
| 442 | ***Capzb*** | Dendritic,C1,C3,Layer1/2,C0,C5 |
| 443 | ***Car11*** | C1,Layer1/2,C0,C5 |
| 444 | ***Car4*** | Layer1/2,Layer3/4 |
| 445 | ***Carmil1*** | Layer1/2 |
| 446 | ***Carmil2*** | C0 |
| 447 | ***Carmil3*** | Layer1/2 |
| 448 | ***Cartpt*** | C1,C3,Layer1/2,C0,Layer3/4 |
| 449 | ***Caskin2*** | Layer1/2 |
| 450 | ***Cbarp*** | Layer1/2,C2,Layer3/4 |
| 451 | ***Cbr1*** | Dendritic,C0,Layer5 |
| 452 | ***Cbr4*** | Layer1/2 |
| 453 | ***Cbx3*** | Dendritic,C0 |
| 454 | ***Cbx7*** | Layer1/2,C0 |
| 455 | ***Ccdc106*** | Layer1/2 |
| 456 | ***Ccdc107*** | Layer5 |
| 457 | ***Ccdc115*** | Layer1/2 |
| 458 | ***Ccdc12*** | C1 |
| 459 | ***Ccdc136*** | C1 |
| 460 | ***Ccdc28a*** | C0 |
| 461 | ***Ccdc32*** | C1 |
| 462 | ***Ccdc58*** | Layer3/4 |
| 463 | ***Ccdc85a*** | Layer1/2,C0 |
| 464 | ***Ccdc85b*** | Layer6,C1,C0 |
| 465 | ***Ccdc92*** | C1 |
| 466 | ***Cck*** | C1,C5 |
| 467 | ***Ccl27a*** | C1 |
| 468 | ***Ccnd1*** | C3 |
| 469 | ***Ccnh*** | C0 |
| 470 | ***Ccnl1*** | Layer1/2 |
| 471 | ***Ccnl2*** | Dendritic |
| 472 | ***Ccsap*** | Layer1/2 |
| 473 | ***Cct2*** | Dendritic,C1,Layer1/2,C0,Layer3/4 |
| 474 | ***Cct5*** | C1,Layer1/2 |
| 475 | ***Cct6a*** | Dendritic,Layer6,C1,Layer1/2,C0,Layer5 |
| 476 | ***Cct8*** | C1,C0 |
| 477 | ***Ccz1*** | C1 |
| 478 | ***Cd2bp2*** | C0 |
| 479 | ***Cd81*** | Dendritic,Layer6,C1,C0 |
| 480 | ***Cdadc1*** | Layer1/2,C0 |
| 481 | ***Cdc123*** | C1,Layer1/2,C0 |
| 482 | ***Cdc23*** | Layer1/2 |
| 483 | ***Cdc42se2*** | Layer1/2,C5 |
| 484 | ***Cdh2*** | C0 |
| 485 | ***Cdip1*** | C1,Layer1/2 |
| 486 | ***Cdipt*** | C1,Layer1/2 |
| 487 | ***Cdk10*** | C1 |
| 488 | ***Cdk11b*** | C0 |
| 489 | ***Cdk16*** | C1,Layer1/2,C2,C0 |
| 490 | ***Cdk17*** | Layer1/2 |
| 491 | ***Cdk2ap1*** | C1 |
| 492 | ***Cdk5*** | C1,Layer1/2,C0 |
| 493 | ***Cdk9*** | C1,Layer5 |
| 494 | ***Cdkl5*** | C1,C0 |
| 495 | ***Cdo1*** | Layer1/2 |
| 496 | ***Cdon*** | Layer1/2 |
| 497 | ***Cds2*** | C1,Layer1/2 |
| 498 | ***Cebpzos*** | C3,C0 |
| 499 | ***Celf2*** | Layer6,C3,Layer1/2,C0 |
| 500 | ***Celf3*** | Layer1/2,Layer3/4 |
| 501 | ***Celf4*** | Dendritic,Somatic,Layer6,Layer1/2,C0,C5,Layer5,Layer3/4 |
| 502 | ***Cend1*** | Layer6,C1,C2,C0 |
| 503 | ***Cep19*** | C1,C0 |
| 504 | ***Cep70*** | Layer1/2 |
| 505 | ***Cers6*** | Layer1/2 |
| 506 | ***Cetn3*** | Layer6,C1,Layer1/2,C0,C5,Layer5 |
| 507 | ***Cfap36*** | Layer6,C1,Layer1/2,Layer5 |
| 508 | ***Cfl1*** | Somatic,Layer6,C1,C3,Layer1/2,C5,Layer3/4 |
| 509 | ***Cfl2*** | C1 |
| 510 | ***Chchd1*** | C1,C0,Layer5 |
| 511 | ***Chchd10*** | Layer6,C1,Layer1/2,C2,C0,Layer5,Layer3/4 |
| 512 | ***Chchd2*** | Dendritic,Somatic,Layer6,C1,C3,Layer1/2,C2,C4,C0,C5,Layer5,Layer3/4 |
| 513 | ***Chchd3*** | C1,C0 |
| 514 | ***Chchd4*** | C1 |
| 515 | ***Chchd6*** | C1 |
| 516 | ***Chchd7*** | C1,Layer1/2,C0 |
| 517 | ***Chd3*** | C1,C2,C0,C5 |
| 518 | ***Chd5*** | C1 |
| 519 | ***Chd9*** | Layer6 |
| 520 | ***Chgb*** | C3,Layer1/2 |
| 521 | ***Chid1*** | Layer1/2 |
| 522 | ***Chka*** | Layer1/2 |
| 523 | ***Chkb*** | C3 |
| 524 | ***Chmp2a*** | C1 |
| 525 | ***Chmp2b*** | C1 |
| 526 | ***Chmp3*** | C1,C3,Layer1/2 |
| 527 | ***Chmp5*** | C1,Layer1/2,C0 |
| 528 | ***Chn1*** | Layer6,C1,C3,Layer1/2,C2,C0,C5,Layer3/4 |
| 529 | ***Chpf*** | C1 |
| 530 | ***Chpf2*** | Layer1/2 |
| 531 | ***Chrac1*** | Layer1/2 |
| 532 | ***Chrd*** | Layer1/2 |
| 533 | ***Chrm1*** | Layer1/2 |
| 534 | ***Chrna4*** | C0 |
| 535 | ***Chst1*** | Layer1/2 |
| 536 | ***Chtop*** | C1,Layer1/2,C0 |
| 537 | ***Churc1*** | Dendritic,C1,C3,Layer1/2,C0 |
| 538 | ***Ciao1*** | C0 |
| 539 | ***Ciao2b*** | C1,C0 |
| 540 | ***Ciao3*** | Layer1/2 |
| 541 | ***Ciapin1*** | C1 |
| 542 | ***Cinp*** | Layer1/2 |
| 543 | ***Cisd1*** | Dendritic,Layer6,C1,C3,Layer1/2,C0,Layer5,Layer3/4 |
| 544 | ***Cisd3*** | C1,C0 |
| 545 | ***Cit*** | C1,C2 |
| 546 | ***Cited2*** | C0 |
| 547 | ***Ckap5*** | C1,C0 |
| 548 | ***Ckmt1*** | C1,C0,Layer5,Layer3/4 |
| 549 | ***Clasp1*** | C1 |
| 550 | ***Clasrp*** | Layer1/2 |
| 551 | ***Clcc1*** | Layer1/2 |
| 552 | ***Clcn2*** | Layer1/2,Layer3/4 |
| 553 | ***Clcn3*** | Dendritic,C1,Layer1/2,C0 |
| 554 | ***Cldn10*** | Dendritic,C0 |
| 555 | ***Cldn5*** | C0 |
| 556 | ***Clec2l*** | C1 |
| 557 | ***Clip3*** | C1,Layer5,Layer3/4 |
| 558 | ***Clk1*** | C0 |
| 559 | ***Clk4*** | Layer1/2 |
| 560 | ***Clns1a*** | C1 |
| 561 | ***Clpp*** | C1 |
| 562 | ***Clptm1*** | C1 |
| 563 | ***Clptm1l*** | C1,Layer1/2 |
| 564 | ***Clstn1*** | Layer6,C1,C3,Layer1/2,C2,C0,Layer5,Layer3/4 |
| 565 | ***Clstn2*** | Layer6,C1,C0 |
| 566 | ***Clstn3*** | C1 |
| 567 | ***Clta*** | C1,Layer1/2,C0 |
| 568 | ***Cltb*** | C1,C0 |
| 569 | ***Cltc*** | C1,Layer1/2,Layer3/4 |
| 570 | ***Clvs2*** | Layer1/2 |
| 571 | ***Cmas*** | C1 |
| 572 | ***Cmpk1*** | Layer6,C1,Layer3/4 |
| 573 | ***Cmtm6*** | Layer1/2 |
| 574 | ***Cnbp*** | Dendritic,Layer6,C1,C3,Layer1/2,C2,C0,C5,Layer5 |
| 575 | ***Cndp2*** | C1 |
| 576 | ***Cnih1*** | Dendritic,C1,C0 |
| 577 | ***Cnih2*** | Dendritic,Layer6,C3,Layer1/2,C0,C5,Layer3/4 |
| 578 | ***Cnksr2*** | C1,Layer1/2 |
| 579 | ***Cnnm1*** | Layer1/2 |
| 580 | ***Cnot1*** | C1 |
| 581 | ***Cnot7*** | C1,Layer1/2 |
| 582 | ***Cnot8*** | C0 |
| 583 | ***Cnp*** | C1 |
| 584 | ***Cnpy2*** | C0 |
| 585 | ***Cnpy3*** | Dendritic |
| 586 | ***Cnr1*** | Dendritic,C1,Layer1/2,C0 |
| 587 | ***Cnrip1*** | Somatic,Layer1/2,C5 |
| 588 | ***Cntfr*** | Layer1/2 |
| 589 | ***Cntn1*** | Dendritic,C1,C2,C0,C5,Layer3/4 |
| 590 | ***Coa3*** | Dendritic,Layer6,C1,Layer1/2,C0,Layer5,Layer3/4 |
| 591 | ***Coa6*** | Dendritic,Layer1/2 |
| 592 | ***Cobl*** | C0 |
| 593 | ***Cog8*** | Layer1/2 |
| 594 | ***Col4a1*** | C3,Layer1/2 |
| 595 | ***Commd3*** | C1,Layer1/2,C0 |
| 596 | ***Commd4*** | Dendritic,C1,Layer1/2,C0,Layer5 |
| 597 | ***Comt*** | Dendritic,Layer6,C1,C3,Layer1/2,C2,C0,Layer3/4 |
| 598 | ***Copa*** | C1,C0 |
| 599 | ***Cope*** | C1,Layer1/2 |
| 600 | ***Copg1*** | C1,C0 |
| 601 | ***Copg2*** | C1 |
| 602 | ***Coprs*** | C1,C0 |
| 603 | ***Cops2*** | C1 |
| 604 | ***Cops3*** | C1 |
| 605 | ***Cops4*** | C1,C0 |
| 606 | ***Cops5*** | C1 |
| 607 | ***Cops6*** | C1 |
| 608 | ***Cops7a*** | C1,Layer1/2,C2 |
| 609 | ***Cops9*** | Dendritic,Somatic,Layer6,C1,Layer1/2,C0 |
| 610 | ***Copz1*** | C1 |
| 611 | ***Coq10a*** | C1,Layer1/2,C0 |
| 612 | ***Coq2*** | Dendritic,C1,Layer1/2,C0,Layer5,Layer3/4 |
| 613 | ***Coq7*** | Dendritic,C1,C0 |
| 614 | ***Coq9*** | C1,Layer1/2 |
| 615 | ***Coro1a*** | Layer1/2 |
| 616 | ***Coro1c*** | C1 |
| 617 | ***Coro2b*** | C1,Layer1/2,Layer3/4 |
| 618 | ***Cox11*** | Layer5 |
| 619 | ***Cox14*** | Dendritic,Layer6,C1,C3,Layer1/2,C0,C5,Layer5,Layer3/4 |
| 620 | ***Cox17*** | Dendritic,Layer6,C1,Layer1/2,C2,C0,Layer5,Layer3/4 |
| 621 | ***Cox20*** | C1,C0 |
| 622 | ***Cox4i1*** | Dendritic,Somatic,Layer6,C1,C3,Layer1/2,C2,C4,C0,C5,Layer5,Layer3/4 |
| 623 | ***Cox5a*** | Dendritic,Layer6,C1,Layer1/2,C0,C5 |
| 624 | ***Cox5b*** | Dendritic,Somatic,Layer6,C1,C3,Layer1/2,C0,C5,Layer5,Layer3/4 |
| 625 | ***Cox6a1*** | Dendritic,Somatic,Layer6,C1,C3,Layer1/2,C2,C0,C5,Layer5,Layer3/4 |
| 626 | ***Cox6b1*** | Dendritic,Somatic,Layer6,C1,C3,Layer1/2,C2,C4,C0,C5,Layer5,Layer3/4 |
| 627 | ***Cox6c*** | Dendritic,Somatic,Layer6,C1,C3,Layer1/2,C2,C4,C0,C5,Layer5,Layer3/4 |
| 628 | ***Cox7a2*** | Dendritic,Somatic,Layer6,C1,C3,Layer1/2,C2,C4,C0,C5,Layer5,Layer3/4 |
| 629 | ***Cox7a2l*** | Dendritic,Layer6,C1,Layer1/2,C4,C0 |
| 630 | ***Cox7b*** | Dendritic,Somatic,Layer6,C1,C3,Layer1/2,C2,C4,C0,C5,Layer5,Layer3/4 |
| 631 | ***Cox7c*** | Dendritic,Somatic,Layer6,C1,C3,Layer1/2,C2,C0,C5,Layer5,Layer3/4 |
| 632 | ***Cox8a*** | Dendritic,Somatic,Layer6,C1,C3,Layer1/2,C2,C4,C0,C5,Layer5,Layer3/4 |
| 633 | ***Cpe*** | Layer1/2,C5 |
| 634 | ***Cplane1*** | Layer5 |
| 635 | ***Cplx1*** | C1,C5 |
| 636 | ***Cpne4*** | Dendritic,C1,C0 |
| 637 | ***Cpne6*** | Layer1/2 |
| 638 | ***Cpne7*** | Layer6,C1,C3,Layer1/2,C0,Layer3/4 |
| 639 | ***Cpne9*** | C2 |
| 640 | ***Cpt1c*** | C1 |
| 641 | ***Crcp*** | Layer1/2 |
| 642 | ***Crebl2*** | C1,C0 |
| 643 | ***Creld1*** | C1 |
| 644 | ***Crip2*** | Layer1/2 |
| 645 | ***Cript*** | C1,Layer1/2,C0 |
| 646 | ***Crk*** | Dendritic,C1,Layer1/2 |
| 647 | ***Crocc*** | Layer1/2 |
| 648 | ***Crot*** | C3 |
| 649 | ***Crtac1*** | Layer1/2,C0 |
| 650 | ***Crtc1*** | Layer1/2 |
| 651 | ***Cry2*** | C1,Layer1/2,C2,C0 |
| 652 | ***Crym*** | C1,C3,C0 |
| 653 | ***Cryzl1*** | C0 |
| 654 | ***Cs*** | C1,C0 |
| 655 | ***Csde1*** | C1 |
| 656 | ***Csf2ra*** | Layer1/2 |
| 657 | ***Csnk1a1*** | C1,C3 |
| 658 | ***Csnk1e*** | C1 |
| 659 | ***Csnk1g2*** | C1,C0 |
| 660 | ***Csnk2a2*** | C1 |
| 661 | ***Csnk2b*** | C1,Layer1/2 |
| 662 | ***Csrnp3*** | C0 |
| 663 | ***Csrp1*** | C0 |
| 664 | ***Cst3*** | Layer1/2 |
| 665 | ***Cst6*** | Layer3/4 |
| 666 | ***Cstb*** | C1 |
| 667 | ***Cstf2*** | Layer1/2,C0 |
| 668 | ***Cstf3*** | Layer1/2,C0 |
| 669 | ***Ctbp1*** | C1,Layer1/2,C2,C0 |
| 670 | ***Ctdnep1*** | C1 |
| 671 | ***Cthrc1*** | Layer1/2 |
| 672 | ***Ctif*** | C1 |
| 673 | ***Ctnnd2*** | Layer1/2,C0 |
| 674 | ***Ctxn1*** | Layer6,C3,Layer1/2,Layer5,Layer3/4 |
| 675 | ***Ctxn2*** | C0 |
| 676 | ***Cuedc2*** | C1 |
| 677 | ***Cul2*** | Layer1/2,C0 |
| 678 | ***Cul3*** | C1 |
| 679 | ***Cuta*** | C1,C0 |
| 680 | ***Cux2*** | Layer1/2 |
| 681 | ***Cwc15*** | Dendritic,Layer6,C1,C0 |
| 682 | ***Cwc27*** | Layer1/2 |
| 683 | ***Cx3cl1*** | C1,C3,Layer1/2 |
| 684 | ***Cxadr*** | Layer5 |
| 685 | ***Cxcl14*** | Layer1/2 |
| 686 | ***Cyb5a*** | Layer6,C0,Layer3/4 |
| 687 | ***Cyc1*** | Dendritic,C1,Layer1/2,C0,C5,Layer5 |
| 688 | ***Cycs*** | Dendritic,Layer6,C1,C3,Layer1/2,C2,C0,C5,Layer5,Layer3/4 |
| 689 | ***Cyfip2*** | C1,Layer1/2,C2,Layer3/4 |
| 690 | ***Cyp2d22*** | C0 |
| 691 | ***Cyp4f15*** | Layer1/2 |
| 692 | ***Cystm1*** | Layer6,C1,Layer1/2,C0,Layer5 |
| 693 | ***D430041D05Rik*** | Layer3/4 |
| 694 | ***D8Ertd738e*** | Layer6,C1,Layer1/2,C0,Layer5 |
| 695 | ***Dab1*** | Layer1/2,C0 |
| 696 | ***Dact3*** | C0 |
| 697 | ***Dad1*** | Dendritic,Layer6,C1,Layer1/2,C0,Layer5 |
| 698 | ***Dalrd3*** | C0 |
| 699 | ***Dap3*** | C1 |
| 700 | ***Dapk1*** | Layer1/2 |
| 701 | ***Dazap2*** | Layer1/2,C0 |
| 702 | ***Dbi*** | C0 |
| 703 | ***Dbn1*** | Layer1/2 |
| 704 | ***Dbndd2*** | C1,C0,C5 |
| 705 | ***Dbnl*** | C1 |
| 706 | ***Dbp*** | C1 |
| 707 | ***Dcaf11*** | C1 |
| 708 | ***Dcaf13*** | Layer1/2 |
| 709 | ***Dcaf7*** | Layer1/2 |
| 710 | ***Dcaf8*** | C0 |
| 711 | ***Dclk2*** | Layer1/2 |
| 712 | ***Dctn1*** | Dendritic,C1,C3,Layer1/2,C0,Layer5,Layer3/4 |
| 713 | ***Dctn2*** | C1 |
| 714 | ***Dctn3*** | Layer6,C1,Layer1/2,C0,Layer5,Layer3/4 |
| 715 | ***Dctn4*** | C1 |
| 716 | ***Dctn5*** | C1,Layer1/2 |
| 717 | ***Dctn6*** | C1,Layer1/2 |
| 718 | ***Dctpp1*** | Layer1/2 |
| 719 | ***Dda1*** | C0 |
| 720 | ***Ddb1*** | C1 |
| 721 | ***Ddb2*** | Layer1/2 |
| 722 | ***Ddhd1*** | C0 |
| 723 | ***Ddit4*** | C0 |
| 724 | ***Ddn*** | C1,C3,Layer1/2,C5 |
| 725 | ***Ddrgk1*** | C0 |
| 726 | ***Ddt*** | C0 |
| 727 | ***Ddx1*** | C1,Layer1/2,C2 |
| 728 | ***Ddx17*** | Layer1/2 |
| 729 | ***Ddx39b*** | Layer1/2,C0 |
| 730 | ***Ddx46*** | Layer1/2 |
| 731 | ***Ddx5*** | C1,C3,Layer1/2,C5,Layer5,Layer3/4 |
| 732 | ***Deaf1*** | C1 |
| 733 | ***Degs1*** | C1 |
| 734 | ***Dele1*** | Layer1/2 |
| 735 | ***Dennd5a*** | C1,C2 |
| 736 | ***Dennd6b*** | Layer1/2,C0 |
| 737 | ***Derl1*** | C1,C0 |
| 738 | ***Dexi*** | C1,C0 |
| 739 | ***Dffb*** | Layer1/2 |
| 740 | ***Dgat1*** | Layer1/2 |
| 741 | ***Dgat2*** | C1,Layer3/4 |
| 742 | ***Dgcr6*** | C1 |
| 743 | ***Dgkb*** | Layer1/2 |
| 744 | ***Dgkd*** | Layer1/2 |
| 745 | ***Dgkz*** | C1,Layer1/2 |
| 746 | ***Dhodh*** | Layer1/2 |
| 747 | ***Dhx30*** | C1,Layer1/2 |
| 748 | ***Dhx33*** | Layer1/2 |
| 749 | ***Diaph2*** | Layer1/2 |
| 750 | ***Dicer1*** | Layer1/2 |
| 751 | ***Dido1*** | Layer1/2 |
| 752 | ***Dipk1b*** | Layer6,C1 |
| 753 | ***Diras2*** | C1 |
| 754 | ***Disp2*** | C1,C2,C0 |
| 755 | ***Disp3*** | Layer1/2 |
| 756 | ***Dkk3*** | Layer1/2,C5 |
| 757 | ***Dlat*** | C1 |
| 758 | ***Dld*** | C1 |
| 759 | ***Dlg1*** | Dendritic |
| 760 | ***Dlg3*** | C1,C5 |
| 761 | ***Dlg4*** | Dendritic,C1,Layer1/2 |
| 762 | ***Dlgap1*** | C1,C3,Layer1/2,C5,Layer5 |
| 763 | ***Dlgap3*** | C1 |
| 764 | ***Dlgap4*** | C1,C3,Layer1/2,Layer3/4 |
| 765 | ***Dlk2*** | Layer1/2 |
| 766 | ***Dlst*** | C1 |
| 767 | ***Dmac2*** | C1,C0 |
| 768 | ***Dmtn*** | Layer1/2,C0 |
| 769 | ***Dmwd*** | C1,C0 |
| 770 | ***Dmxl2*** | Dendritic,C1,Layer1/2,C2 |
| 771 | ***Dnaja1*** | C1,Layer5 |
| 772 | ***Dnaja2*** | Layer6,C1,C3,Layer1/2,C2,C0 |
| 773 | ***Dnaja3*** | C0 |
| 774 | ***Dnaja4*** | Layer1/2 |
| 775 | ***Dnajb11*** | Layer1/2 |
| 776 | ***Dnajb6*** | C1,C3,Layer1/2,Layer5 |
| 777 | ***Dnajc10*** | Dendritic |
| 778 | ***Dnajc15*** | C1 |
| 779 | ***Dnajc16*** | Layer1/2 |
| 780 | ***Dnajc19*** | C1,Layer1/2,C0 |
| 781 | ***Dnajc27*** | C1 |
| 782 | ***Dnajc30*** | C1,Layer1/2,C0 |
| 783 | ***Dnajc5*** | C1 |
| 784 | ***Dnajc6*** | Dendritic,Layer6,C1,Layer1/2,C0,Layer3/4 |
| 785 | ***Dnajc7*** | Layer1/2 |
| 786 | ***Dnajc8*** | Layer6,C0 |
| 787 | ***Dnajc9*** | Layer1/2 |
| 788 | ***Dnal1*** | Layer1/2 |
| 789 | ***Dnal4*** | Layer6,Layer1/2,C0 |
| 790 | ***Dner*** | C0 |
| 791 | ***Dnlz*** | C1,C0 |
| 792 | ***Dnm1*** | C1,Layer1/2,C2,C0 |
| 793 | ***Dnm1l*** | C1,Layer1/2 |
| 794 | ***Dnpep*** | C1,Layer1/2 |
| 795 | ***Dock10*** | C3 |
| 796 | ***Dock9*** | C0 |
| 797 | ***Dohh*** | C1 |
| 798 | ***Dop1b*** | Layer1/2,C0 |
| 799 | ***Dpagt1*** | Layer1/2 |
| 800 | ***Dph3*** | C1,C0 |
| 801 | ***Dpm1*** | Layer6,C0 |
| 802 | ***Dpm2*** | Layer1/2 |
| 803 | ***Dpp10*** | Layer1/2 |
| 804 | ***Dpy19l1*** | C1 |
| 805 | ***Dpy19l3*** | Layer1/2 |
| 806 | ***Dpy30*** | C1,C0 |
| 807 | ***Dpysl4*** | Layer1/2,C0 |
| 808 | ***Drap1*** | Dendritic,Layer6,C1,C3,Layer1/2,C2,C4,C0,C5,Layer5,Layer3/4 |
| 809 | ***Drc1*** | Layer1/2 |
| 810 | ***Drg1*** | C1 |
| 811 | ***Drg2*** | C1,C0 |
| 812 | ***Dst*** | Layer6,C1,C3,C0 |
| 813 | ***Dstn*** | C1,Layer1/2,Layer3/4 |
| 814 | ***Dtd1*** | C1,Layer1/2,C0 |
| 815 | ***Dtnbp1*** | C1,Layer1/2 |
| 816 | ***Dtx3*** | C1 |
| 817 | ***Dtymk*** | Layer1/2,C0 |
| 818 | ***Dusp11*** | Layer1/2 |
| 819 | ***Dusp14*** | Layer1/2 |
| 820 | ***Dusp28*** | Layer1/2 |
| 821 | ***Dusp5*** | Layer1/2 |
| 822 | ***Dync1h1*** | C3 |
| 823 | ***Dync1i1*** | C1,C2,C0 |
| 824 | ***Dync1i2*** | C0 |
| 825 | ***Dync1li1*** | Layer1/2 |
| 826 | ***Dynll1*** | Dendritic,Somatic,Layer6,C1,C3,Layer1/2,C2,C4,C0,C5,Layer5,Layer3/4 |
| 827 | ***Dynlrb1*** | Dendritic,Layer6,C1,Layer1/2,C0,Layer5,Layer3/4 |
| 828 | ***Dynlt1a*** | Layer1/2 |
| 829 | ***Dynlt3*** | C1,C5 |
| 830 | ***Dyrk1a*** | C0 |
| 831 | ***Dzank1*** | Dendritic,C0,Layer5 |
| 832 | ***E130307A14Rik*** | Layer1/2 |
| 833 | ***Ech1*** | C0 |
| 834 | ***Echs1*** | C0 |
| 835 | ***Eci2*** | C0 |
| 836 | ***Edf1*** | Dendritic,Layer6,C1,C3,Layer1/2,C0,C5,Layer5 |
| 837 | ***Eef1a1*** | Dendritic,Somatic,Layer6,C3,Layer1/2,C0,C5,Layer5,Layer3/4 |
| 838 | ***Eef1b2*** | Dendritic,Somatic,Layer6,C1,C3,Layer1/2,C0 |
| 839 | ***Eef1e1*** | C1,C0 |
| 840 | ***Eef1g*** | Dendritic,C1 |
| 841 | ***Efhd2*** | Layer6,C1,C3,Layer1/2,C0,Layer5,Layer3/4 |
| 842 | ***Efl1*** | Layer1/2 |
| 843 | ***Efna3*** | Layer1/2 |
| 844 | ***Efnb3*** | C0 |
| 845 | ***Efr3a*** | C0 |
| 846 | ***Eftud2*** | C1,Layer1/2 |
| 847 | ***Egln1*** | Layer1/2,C0 |
| 848 | ***Egr3*** | C3,Layer1/2 |
| 849 | ***Ehd3*** | C1 |
| 850 | ***Ei24*** | C1,Layer1/2 |
| 851 | ***Eid1*** | Somatic,C0,C5,Layer5 |
| 852 | ***Eid2b*** | Layer1/2 |
| 853 | ***Eif1*** | Dendritic,Layer6,C1,C3,Layer1/2,C2,C0,C5,Layer5 |
| 854 | ***Eif1ad*** | C0 |
| 855 | ***Eif1ax*** | C1,Layer1/2,C0 |
| 856 | ***Eif1b*** | Layer6,C1,Layer1/2,Layer5 |
| 857 | ***Eif2a*** | Layer1/2 |
| 858 | ***Eif2b1*** | Layer1/2 |
| 859 | ***Eif2b5*** | C1 |
| 860 | ***Eif2d*** | Layer1/2 |
| 861 | ***Eif2s2*** | Layer6,C1 |
| 862 | ***Eif3c*** | C1,C0 |
| 863 | ***Eif3e*** | C0 |
| 864 | ***Eif3f*** | Dendritic,Layer6,C1,C0 |
| 865 | ***Eif3h*** | Layer6,C1,C0,Layer5 |
| 866 | ***Eif3i*** | C1,C0 |
| 867 | ***Eif3k*** | Dendritic,Layer6,C1,Layer1/2,C0 |
| 868 | ***Eif3m*** | C1,C0 |
| 869 | ***Eif4a1*** | Layer6,C1,Layer1/2 |
| 870 | ***Eif4a2*** | Dendritic,Somatic,Layer6,C1,Layer1/2,C0,Layer5,Layer3/4 |
| 871 | ***Eif4a3*** | C1,C0 |
| 872 | ***Eif4e*** | C1 |
| 873 | ***Eif4g1*** | C1,Layer1/2,C0 |
| 874 | ***Eif4g2*** | C1,Layer1/2,C2 |
| 875 | ***Eif4g3*** | C1,C3,Layer1/2 |
| 876 | ***Eif4h*** | C1,Layer1/2 |
| 877 | ***Eif5*** | C1 |
| 878 | ***Eif5a*** | Dendritic,Somatic,Layer6,C1,C3,Layer1/2,C4,C0,C5,Layer5,Layer3/4 |
| 879 | ***Eif5a2*** | C1,C2,C0 |
| 880 | ***Eif6*** | C1 |
| 881 | ***Elavl4*** | C1,C0 |
| 882 | ***Elk1*** | Layer1/2 |
| 883 | ***Elmo1*** | C1,C0 |
| 884 | ***Elmo2*** | Layer1/2,C5 |
| 885 | ***Elmod1*** | Dendritic,C1,Layer5 |
| 886 | ***Elob*** | Dendritic,Somatic,Layer6,C1,C3,Layer1/2,C4,C0,C5,Layer5,Layer3/4 |
| 887 | ***Eloc*** | Dendritic,Layer6,C1,C3,Layer1/2,C2,C0 |
| 888 | ***Elovl6*** | C1 |
| 889 | ***Elp1*** | C1 |
| 890 | ***Elp5*** | Layer6,C1,C0 |
| 891 | ***Emc1*** | C1 |
| 892 | ***Emc10*** | C1,Layer1/2 |
| 893 | ***Emc2*** | Dendritic,C1,Layer1/2,C0 |
| 894 | ***Emc3*** | C1 |
| 895 | ***Emc4*** | Layer6,C1,Layer1/2,C0 |
| 896 | ***Emc6*** | Dendritic,C1 |
| 897 | ***Emc7*** | C1,C0 |
| 898 | ***Emc8*** | C0 |
| 899 | ***Emc9*** | C1 |
| 900 | ***Emg1*** | C1,Layer1/2 |
| 901 | ***Eml1*** | Layer1/2 |
| 902 | ***Eml6*** | Layer1/2 |
| 903 | ***Enc1*** | Dendritic,Layer6,C3,Layer1/2,C0,C5 |
| 904 | ***Endog*** | C0 |
| 905 | ***Enho*** | Dendritic,C1,C0 |
| 906 | ***Eno2*** | C1 |
| 907 | ***Ensa*** | Layer1/2,C5 |
| 908 | ***Entpd6*** | C0 |
| 909 | ***Entr1*** | Layer1/2 |
| 910 | ***Eny2*** | C1 |
| 911 | ***Ep400*** | C0 |
| 912 | ***Epb41l2*** | Layer1/2 |
| 913 | ***Epb41l3*** | C1 |
| 914 | ***Epb41l4aos*** | C0,Layer5 |
| 915 | ***Epc1*** | Layer1/2 |
| 916 | ***Epdr1*** | Layer1/2,C0 |
| 917 | ***Epha4*** | C1 |
| 918 | ***Ephx4*** | C1,C0 |
| 919 | ***Epm2aip1*** | C0 |
| 920 | ***Eprs*** | C0 |
| 921 | ***Eps15*** | C1,C0 |
| 922 | ***Erc2*** | Dendritic,C1,Layer1/2 |
| 923 | ***Ercc5*** | Layer1/2 |
| 924 | ***Ercc6*** | Layer1/2 |
| 925 | ***Esd*** | C1,C0 |
| 926 | ***Etfrf1*** | Layer1/2 |
| 927 | ***Etnk1*** | C1,C2,C0 |
| 928 | ***Etnppl*** | Layer1/2 |
| 929 | ***Ets2*** | Layer1/2,Layer3/4 |
| 930 | ***Etv5*** | C1 |
| 931 | ***Evc2*** | Layer1/2 |
| 932 | ***Ewsr1*** | C1,Layer1/2,Layer5 |
| 933 | ***Exd2*** | C0 |
| 934 | ***Exoc1*** | Layer1/2,C0 |
| 935 | ***Exoc3*** | C1 |
| 936 | ***Exosc4*** | C1 |
| 937 | ***Exosc7*** | Layer1/2 |
| 938 | ***Exosc9*** | Layer1/2 |
| 939 | ***Extl2*** | C1 |
| 940 | ***Ezh1*** | C0 |
| 941 | ***F3*** | C0 |
| 942 | ***F730043M19Rik*** | Layer1/2 |
| 943 | ***Faah*** | C1,Layer1/2 |
| 944 | ***Fabp3*** | Layer6,C1 |
| 945 | ***Fabp5*** | C1 |
| 946 | ***Fam104a*** | C1 |
| 947 | ***Fam120a*** | C1 |
| 948 | ***Fam120b*** | C0 |
| 949 | ***Fam126b*** | C1 |
| 950 | ***Fam131a*** | C1,C3,Layer1/2 |
| 951 | ***Fam155a*** | C0 |
| 952 | ***Fam162a*** | C1 |
| 953 | ***Fam163b*** | Layer1/2 |
| 954 | ***Fam168b*** | Layer1/2,Layer3/4 |
| 955 | ***Fam173a*** | C1,Layer1/2,C0 |
| 956 | ***Fam174a*** | C0,C5 |
| 957 | ***Fam189a1*** | Layer1/2 |
| 958 | ***Fam214a*** | C0 |
| 959 | ***Fam241b*** | C0 |
| 960 | ***Fam49a*** | C1,C5 |
| 961 | ***Fam57b*** | C0 |
| 962 | ***Fam8a1*** | Dendritic,C1 |
| 963 | ***Fam92a*** | Layer1/2 |
| 964 | ***Far1*** | C1,Layer1/2,C0 |
| 965 | ***Fastk*** | C1,Layer1/2 |
| 966 | ***Fau*** | Dendritic,Somatic,Layer6,C1,C3,Layer1/2,C4,C0,C5,Layer5,Layer3/4 |
| 967 | ***Fbf1*** | Layer1/2 |
| 968 | ***Fbh1*** | Layer1/2,C0 |
| 969 | ***Fbxl16*** | Layer6,C1,Layer1/2,C2,C0,Layer5,Layer3/4 |
| 970 | ***Fbxl2*** | Layer1/2 |
| 971 | ***Fbxo11*** | Layer1/2 |
| 972 | ***Fbxo2*** | C0 |
| 973 | ***Fbxo27*** | Layer1/2 |
| 974 | ***Fbxo41*** | Layer1/2,C0 |
| 975 | ***Fbxo44*** | C1,C2,C0 |
| 976 | ***Fbxo6*** | Layer1/2 |
| 977 | ***Fbxo9*** | C1,C0 |
| 978 | ***Fbxw5*** | C1 |
| 979 | ***Fbxw7*** | C1,C0 |
| 980 | ***Fcho2*** | Layer1/2 |
| 981 | ***Fdx1*** | Layer1/2 |
| 982 | ***Fdx2*** | Layer6,C1,Layer1/2,C0,C5 |
| 983 | ***Fez1*** | C1,Layer1/2,Layer5 |
| 984 | ***Fez2*** | C0 |
| 985 | ***Fezf2*** | Layer6 |
| 986 | ***Fgf12*** | C1 |
| 987 | ***Fgf13*** | Dendritic,C1,C2,C0 |
| 988 | ***Fgfr1*** | C0 |
| 989 | ***Fgfr1op2*** | C1,Layer1/2 |
| 990 | ***Fggy*** | Layer1/2 |
| 991 | ***Fh1*** | C1 |
| 992 | ***Fhl2*** | Layer1/2 |
| 993 | ***Filip1*** | Layer1/2 |
| 994 | ***Fkbp1a*** | C1,C3,Layer1/2,C5,Layer3/4 |
| 995 | ***Fkbp1b*** | Layer1/2,C0 |
| 996 | ***Fkbp2*** | C1,C0 |
| 997 | ***Fkbp3*** | Dendritic,Somatic,Layer6,C1,Layer1/2,C2,C0,Layer3/4 |
| 998 | ***Fkbp8*** | Layer6,C1,Layer1/2,C0 |
| 999 | ***Fkrp*** | Layer1/2 |
| 1000 | ***Flii*** | C0 |
| 1001 | ***Flot1*** | Layer1/2 |
| 1002 | ***Flywch1*** | Layer6,C1,C2,C0,Layer5 |
| 1003 | ***Fmc1*** | Dendritic,Layer6,C1,Layer1/2,C0,Layer5 |
| 1004 | ***Fmnl1*** | Layer1/2 |
| 1005 | ***Fn3k*** | C0 |
| 1006 | ***Fnbp4*** | Layer1/2 |
| 1007 | ***Fndc4*** | C1 |
| 1008 | ***Fnip1*** | Layer1/2 |
| 1009 | ***Foxg1*** | C1 |
| 1010 | ***Frmd4a*** | Layer1/2 |
| 1011 | ***Frrs1l*** | C1,Layer1/2,C0,C5 |
| 1012 | ***Fry*** | C1,C0 |
| 1013 | ***Fsd1*** | Layer1/2 |
| 1014 | ***Fth1*** | Dendritic,Somatic,Layer6,C3,Layer1/2,C0,C5,Layer5,Layer3/4 |
| 1015 | ***Ftl1*** | Somatic,Layer6,C0 |
| 1016 | ***Ftx*** | Layer1/2 |
| 1017 | ***Fundc1*** | C0 |
| 1018 | ***Fundc2*** | C1,C0 |
| 1019 | ***Fuom*** | C1,Layer5 |
| 1020 | ***Fut9*** | Layer6,C0,Layer5 |
| 1021 | ***Fxr2*** | C1,C0 |
| 1022 | ***Fxyd7*** | C1,Layer1/2,C0 |
| 1023 | ***Fzd3*** | Layer1/2,C0 |
| 1024 | ***G3bp2*** | C1,C0 |
| 1025 | ***Gabarap*** | C1,C0 |
| 1026 | ***Gabarapl1*** | Layer6,C1,Layer1/2,C2,C0,Layer5,Layer3/4 |
| 1027 | ***Gabarapl2*** | Dendritic,Layer6,C1,C3,Layer1/2,C0,Layer5,Layer3/4 |
| 1028 | ***Gabbr1*** | C1 |
| 1029 | ***Gabbr2*** | C1,Layer1/2,C4,C0 |
| 1030 | ***Gabra1*** | C2 |
| 1031 | ***Gabra4*** | C5 |
| 1032 | ***Gabrb3*** | Layer1/2,C2 |
| 1033 | ***Gabrg2*** | Layer1/2 |
| 1034 | ***Gad1*** | C1 |
| 1035 | ***Gad2*** | C1 |
| 1036 | ***Gadd45b*** | Layer1/2 |
| 1037 | ***Gak*** | C1 |
| 1038 | ***Galnt9*** | Layer6,C1,C0 |
| 1039 | ***Gars*** | C1 |
| 1040 | ***Gas5*** | Dendritic,Somatic,Layer6,C1,C3,Layer1/2,C0,Layer5 |
| 1041 | ***Gatad1*** | C1 |
| 1042 | ***Gatc*** | Layer3/4 |
| 1043 | ***Gatd3a*** | Layer1/2 |
| 1044 | ***Gatm*** | C1 |
| 1045 | ***Gba2*** | Layer1/2,C0 |
| 1046 | ***Gbf1*** | C0 |
| 1047 | ***Gclc*** | C0 |
| 1048 | ***Gclm*** | C1,C0 |
| 1049 | ***Gcsh*** | C1 |
| 1050 | ***Gdap1*** | C1,C0 |
| 1051 | ***Gdap1l1*** | C1 |
| 1052 | ***Gde1*** | Layer6,C1,Layer1/2 |
| 1053 | ***Gdi1*** | Dendritic,Somatic,Layer6,C1,Layer1/2,C0,C5,Layer5,Layer3/4 |
| 1054 | ***Gdi2*** | C1 |
| 1055 | ***Get4*** | C1,C0 |
| 1056 | ***Gfm2*** | Layer1/2 |
| 1057 | ***Gfod1*** | Layer1/2,C0 |
| 1058 | ***Gfra4*** | C1,Layer1/2,C0 |
| 1059 | ***Gga1*** | C1 |
| 1060 | ***Gga3*** | C1,Layer1/2,C0 |
| 1061 | ***Ggct*** | C1 |
| 1062 | ***Ggt7*** | C1,Layer1/2,C2,C0 |
| 1063 | ***Ghitm*** | Somatic,C1,C3,Layer1/2,C0,C5 |
| 1064 | ***Gid8*** | Layer1/2 |
| 1065 | ***Ginm1*** | Layer1/2 |
| 1066 | ***Git1*** | C1 |
| 1067 | ***Gldc*** | Layer1/2 |
| 1068 | ***Gle1*** | C0 |
| 1069 | ***Glo1*** | C1,C0 |
| 1070 | ***Glrb*** | Dendritic,C1,C3,Layer1/2,C0,Layer5 |
| 1071 | ***Glrx*** | C1,C0 |
| 1072 | ***Glrx2*** | Dendritic,C1,Layer1/2 |
| 1073 | ***Glrx3*** | C1 |
| 1074 | ***Glrx5*** | C1,C0 |
| 1075 | ***Gls*** | Dendritic,Layer6,C1,Layer1/2,C0,C5,Layer5,Layer3/4 |
| 1076 | ***Glud1*** | C0 |
| 1077 | ***Glyr1*** | C0 |
| 1078 | ***Gm10419*** | Layer1/2,C0 |
| 1079 | ***Gm11549*** | Layer6,Layer1/2,Layer3/4 |
| 1080 | ***Gm11808*** | Dendritic,C1,Layer1/2,C0,Layer5 |
| 1081 | ***Gm16286*** | C1,Layer1/2,C0 |
| 1082 | ***Gm1673*** | C1 |
| 1083 | ***Gm17018*** | Dendritic,C1 |
| 1084 | ***Gm19531*** | Layer1/2 |
| 1085 | ***Gm2000*** | Layer1/2 |
| 1086 | ***Gm27032*** | Layer6,Layer1/2 |
| 1087 | ***Gm28151*** | Layer1/2 |
| 1088 | ***Gm34466*** | Layer1/2 |
| 1089 | ***Gm3764*** | C3,Layer1/2,C0,Layer3/4 |
| 1090 | ***Gm3839*** | C1,Layer1/2,C0,Layer5 |
| 1091 | ***Gm42418*** | Dendritic |
| 1092 | ***Gm45716*** | Dendritic |
| 1093 | ***Gm49207*** | Layer1/2 |
| 1094 | ***Gm49980*** | Dendritic,Layer6,C1,C3,Layer1/2,C2,C0,C5,Layer5 |
| 1095 | ***Gm9885*** | Layer1/2 |
| 1096 | ***Gmeb1*** | Layer1/2 |
| 1097 | ***Gmppa*** | Layer1/2 |
| 1098 | ***Gnai1*** | Dendritic,C1 |
| 1099 | ***Gnal*** | C1 |
| 1100 | ***Gnao1*** | C1,C3,Layer1/2,C5 |
| 1101 | ***Gnaq*** | C1 |
| 1102 | ***Gnas*** | Dendritic,Somatic,Layer6,C1,C3,Layer1/2,C2,C4,C0,C5,Layer5,Layer3/4 |
| 1103 | ***Gnb1*** | C1 |
| 1104 | ***Gnb2*** | Layer1/2,Layer5 |
| 1105 | ***Gnb5*** | Layer1/2 |
| 1106 | ***Gng10*** | Layer1/2 |
| 1107 | ***Gng13*** | Dendritic,Somatic,Layer6,C2,C0 |
| 1108 | ***Gng2*** | Dendritic |
| 1109 | ***Gng3*** | Layer6,C1,Layer1/2,C0 |
| 1110 | ***Gng5*** | C0 |
| 1111 | ***Gnl1*** | C1 |
| 1112 | ***Gnpat*** | Layer5 |
| 1113 | ***Gnpda2*** | Layer1/2 |
| 1114 | ***Golga7*** | C1 |
| 1115 | ***Gorasp2*** | Layer1/2 |
| 1116 | ***Got1*** | C1,C0 |
| 1117 | ***Got2*** | C1,C2 |
| 1118 | ***Gpat4*** | C1 |
| 1119 | ***Gpcpd1*** | Layer1/2,C0 |
| 1120 | ***Gphn*** | Dendritic,Layer1/2,C0 |
| 1121 | ***Gpi1*** | C1,C5 |
| 1122 | ***Gpkow*** | Layer1/2 |
| 1123 | ***Gpm6a*** | Layer6,C3,Layer1/2,Layer5,Layer3/4 |
| 1124 | ***Gpr158*** | Dendritic,C1,Layer1/2,C0,Layer3/4 |
| 1125 | ***Gpr162*** | C1,Layer1/2,C2,C0,C5 |
| 1126 | ***Gpr19*** | Layer1/2 |
| 1127 | ***Gpr22*** | Layer1/2,C0 |
| 1128 | ***Gprin1*** | Layer1/2,C0 |
| 1129 | ***Gps1*** | Dendritic,C1,C3,Layer1/2,C0 |
| 1130 | ***Gpsm1*** | Layer1/2 |
| 1131 | ***Gpx1*** | C1,Layer1/2 |
| 1132 | ***Gpx4*** | Dendritic,Somatic,Layer6,C1,C3,Layer1/2,C4,C0,Layer5,Layer3/4 |
| 1133 | ***Gramd1a*** | C1,Layer1/2 |
| 1134 | ***Grasp*** | Layer1/2 |
| 1135 | ***Grb14*** | C1 |
| 1136 | ***Grb2*** | Layer1/2 |
| 1137 | ***Grcc10*** | Dendritic,Somatic,Layer6,C1,C3,Layer1/2,C2,C4,C0,C5,Layer5,Layer3/4 |
| 1138 | ***Gria1*** | Dendritic,Layer6,C5 |
| 1139 | ***Gria2*** | C3,Layer1/2 |
| 1140 | ***Gria3*** | C1,C3,Layer1/2,C2 |
| 1141 | ***Gria4*** | C1 |
| 1142 | ***Grik5*** | C5 |
| 1143 | ***Grin1*** | C1,Layer1/2,C2 |
| 1144 | ***Grin2a*** | C1,Layer1/2 |
| 1145 | ***Grina*** | C1,Layer1/2 |
| 1146 | ***Grk2*** | C0 |
| 1147 | ***Grk3*** | C1,Layer1/2,C0 |
| 1148 | ***Grk6*** | C1 |
| 1149 | ***Grm1*** | Layer1/2 |
| 1150 | ***Grm5*** | C3 |
| 1151 | ***Grpel1*** | C1 |
| 1152 | ***Grsf1*** | C1,C0 |
| 1153 | ***Gse1*** | C0 |
| 1154 | ***Gsg1l*** | Layer1/2 |
| 1155 | ***Gsk3a*** | Layer1/2 |
| 1156 | ***Gsk3b*** | C2 |
| 1157 | ***Gstm1*** | Dendritic,C3,C0 |
| 1158 | ***Gstm4*** | Layer1/2 |
| 1159 | ***Gstm5*** | Dendritic,Layer6,C1,C2,C0,Layer5 |
| 1160 | ***Gsto1*** | C0 |
| 1161 | ***Gt(ROSA)26Sor*** | Layer1/2 |
| 1162 | ***Gtdc1*** | Layer1/2 |
| 1163 | ***Gtf2a2*** | C1,Layer1/2,C0,Layer3/4 |
| 1164 | ***Gtf2b*** | Layer1/2,C0 |
| 1165 | ***Gtf2h5*** | Layer6,C1,Layer1/2,C0,Layer3/4 |
| 1166 | ***Gtf3c1*** | C1,Layer1/2 |
| 1167 | ***Gtf3c3*** | Layer1/2 |
| 1168 | ***Gtpbp4*** | Layer1/2 |
| 1169 | ***Gtpbp6*** | Layer1/2 |
| 1170 | ***Gucy1a1*** | Dendritic,C1,Layer1/2,C0 |
| 1171 | ***Gucy1b1*** | Layer1/2,Layer3/4 |
| 1172 | ***Guf1*** | Layer1/2 |
| 1173 | ***Guk1*** | Dendritic,Layer6,C1,C3,Layer1/2,C0,C5,Layer5,Layer3/4 |
| 1174 | ***H1f0*** | C0 |
| 1175 | ***H2afj*** | C1,Layer1/2 |
| 1176 | ***H2afx*** | Layer1/2 |
| 1177 | ***H2afy*** | Dendritic,C0 |
| 1178 | ***H2afz*** | Dendritic,Somatic,Layer6,C1,C3,Layer1/2,C2,C0,Layer5,Layer3/4 |
| 1179 | ***H3f3a*** | Dendritic,Layer6,C1,C3,Layer1/2,C0,C5,Layer5,Layer3/4 |
| 1180 | ***H3f3b*** | Dendritic,Layer6,C1,C3,Layer1/2,C2,C4,C0,C5,Layer5,Layer3/4 |
| 1181 | ***Habp4*** | C1,Layer1/2,C2 |
| 1182 | ***Hacd3*** | C1,Layer1/2 |
| 1183 | ***Hace1*** | Layer1/2 |
| 1184 | ***Hagh*** | Layer6,C1 |
| 1185 | ***Haghl*** | C1 |
| 1186 | ***Hccs*** | Dendritic,Somatic,Layer6,C1,C3,Layer1/2,C2,C0,Layer5,Layer3/4 |
| 1187 | ***Hcfc1r1*** | Somatic,Layer6,C1,Layer1/2,C0,Layer5 |
| 1188 | ***Hcn1*** | C0 |
| 1189 | ***Hdac11*** | C1 |
| 1190 | ***Hdac2*** | Layer1/2 |
| 1191 | ***Hdac3*** | C1 |
| 1192 | ***Hdac7*** | Layer1/2 |
| 1193 | ***Hdgf*** | C1 |
| 1194 | ***Heatr1*** | Layer1/2 |
| 1195 | ***Hebp1*** | C1,Layer1/2,C0 |
| 1196 | ***Hectd1*** | C1,C0 |
| 1197 | ***Hectd4*** | C1 |
| 1198 | ***Hemk1*** | Layer1/2 |
| 1199 | ***Herc1*** | C1,Layer1/2 |
| 1200 | ***Herc2*** | C1,C2,C0 |
| 1201 | ***Herc3*** | Layer3/4 |
| 1202 | ***Hgsnat*** | C1 |
| 1203 | ***Higd1a*** | C1,Layer1/2,C0 |
| 1204 | ***Higd2a*** | Dendritic,Layer6,C1,Layer1/2,C0,C5,Layer5,Layer3/4 |
| 1205 | ***Hilpda*** | Layer1/2 |
| 1206 | ***Hint1*** | Dendritic,Somatic,Layer6,C1,C3,Layer1/2,C4,C0,C5,Layer5,Layer3/4 |
| 1207 | ***Hint2*** | C1 |
| 1208 | ***Hipk3*** | C1,C0 |
| 1209 | ***Hira*** | Layer1/2,C0 |
| 1210 | ***Hivep2*** | Layer6,C1,C0 |
| 1211 | ***Hmbs*** | Layer1/2 |
| 1212 | ***Hmgb1*** | Layer6,C1,C3,Layer1/2,C4,C0,Layer5,Layer3/4 |
| 1213 | ***Hmgcl*** | Layer1/2 |
| 1214 | ***Hmgn2*** | Layer6,Layer1/2,Layer3/4 |
| 1215 | ***Hmgn3*** | C5 |
| 1216 | ***Hmox2*** | C1 |
| 1217 | ***Hnrnpa1*** | C5 |
| 1218 | ***Hnrnpa2b1*** | Dendritic,Layer6,C3,Layer1/2,C0,C5,Layer5,Layer3/4 |
| 1219 | ***Hnrnpa3*** | Layer1/2,C0 |
| 1220 | ***Hnrnpc*** | C1,Layer1/2,C0 |
| 1221 | ***Hnrnpd*** | Layer1/2,C0 |
| 1222 | ***Hnrnpdl*** | Layer1/2,Layer5 |
| 1223 | ***Hnrnph1*** | C3,Layer1/2,C2,C0,Layer5,Layer3/4 |
| 1224 | ***Hnrnph2*** | C1,Layer1/2 |
| 1225 | ***Hnrnph3*** | C0 |
| 1226 | ***Hnrnpk*** | C3,Layer1/2,C4,Layer3/4 |
| 1227 | ***Hnrnpm*** | C1,Layer1/2 |
| 1228 | ***Hnrnpu*** | C1,C3,Layer1/2,C5 |
| 1229 | ***Homer2*** | Layer1/2 |
| 1230 | ***Hopx*** | C1,C0,Layer5 |
| 1231 | ***Hp1bp3*** | Dendritic,C1,Layer1/2,C2,C0,Layer5,Layer3/4 |
| 1232 | ***Hpca*** | Layer6 |
| 1233 | ***Hpcal1*** | C5 |
| 1234 | ***Hprt*** | Dendritic,Layer6,C1,C3,Layer1/2,C2,C4,Layer5 |
| 1235 | ***Hps5*** | Layer1/2 |
| 1236 | ***Hras*** | Dendritic,Layer6,C1,Layer1/2,C5,Layer5 |
| 1237 | ***Hrh3*** | C2 |
| 1238 | ***Hs3st4*** | C0 |
| 1239 | ***Hsbp1*** | Dendritic,Somatic,Layer6,C1,C3,Layer1/2,C4,C0,C5,Layer5,Layer3/4 |
| 1240 | ***Hsd11b1*** | C0 |
| 1241 | ***Hsd17b10*** | C0 |
| 1242 | ***Hsd17b12*** | C1 |
| 1243 | ***Hsd17b4*** | C0 |
| 1244 | ***Hsf1*** | Layer1/2 |
| 1245 | ***Hsp90aa1*** | Dendritic,Layer6,C1,C3 |
| 1246 | ***Hsp90ab1*** | C1,Layer1/2 |
| 1247 | ***Hspa12a*** | C1 |
| 1248 | ***Hspa4l*** | C1,C2 |
| 1249 | ***Hspa9*** | C1 |
| 1250 | ***Hspb11*** | Layer1/2 |
| 1251 | ***Hspbp1*** | C1 |
| 1252 | ***Hspd1*** | C1,C0 |
| 1253 | ***Hspe1*** | Layer6,C1,Layer1/2,C0,Layer5,Layer3/4 |
| 1254 | ***Htatsf1*** | C1 |
| 1255 | ***Htra1*** | Dendritic,C3 |
| 1256 | ***Huwe1*** | C1 |
| 1257 | ***Hypk*** | C1,Layer1/2 |
| 1258 | ***Ica1*** | C1,C0 |
| 1259 | ***Ica1l*** | Layer1/2 |
| 1260 | ***Icam5*** | Layer6,C1,C3,Layer1/2 |
| 1261 | ***Idh3a*** | Layer6,C1,C0,C5,Layer3/4 |
| 1262 | ***Idh3b*** | Dendritic,Layer6,C1,C3,Layer1/2,C2,C0,Layer3/4 |
| 1263 | ***Idh3g*** | Dendritic,C1,C0 |
| 1264 | ***Ids*** | Layer1/2 |
| 1265 | ***Ift20*** | C1,C0,Layer5 |
| 1266 | ***Igf1r*** | C0 |
| 1267 | ***Igfbp6*** | Layer1/2 |
| 1268 | ***Igfbp7*** | C2,C0 |
| 1269 | ***Igflr1*** | Layer1/2 |
| 1270 | ***Igfn1*** | Layer1/2 |
| 1271 | ***Igsf3*** | Layer1/2 |
| 1272 | ***Ildr2*** | C2 |
| 1273 | ***Immt*** | C1 |
| 1274 | ***Imp3*** | Layer6,Layer1/2 |
| 1275 | ***Impa1*** | C1,Layer1/2,C0 |
| 1276 | ***Ina*** | C0 |
| 1277 | ***Inip*** | Layer1/2 |
| 1278 | ***Inka2*** | Layer1/2 |
| 1279 | ***Inpp4a*** | C2 |
| 1280 | ***Inpp5a*** | C1 |
| 1281 | ***Inpp5b*** | Layer1/2 |
| 1282 | ***Inpp5j*** | C0 |
| 1283 | ***Inpp5k*** | Layer6,Layer1/2,C0 |
| 1284 | ***Insyn1*** | C1 |
| 1285 | ***Ints12*** | Layer1/2 |
| 1286 | ***Ints6l*** | Layer1/2 |
| 1287 | ***Ints7*** | Layer1/2 |
| 1288 | ***Ip6k2*** | Layer1/2,C0 |
| 1289 | ***Ipo5*** | Layer1/2,C0 |
| 1290 | ***Ipo7*** | C1,Layer1/2 |
| 1291 | ***Iqsec2*** | C1,Layer1/2 |
| 1292 | ***Iqsec3*** | C0 |
| 1293 | ***Irak1*** | Dendritic |
| 1294 | ***Irs2*** | Layer1/2,C0 |
| 1295 | ***Isca1*** | Dendritic,C1,Layer1/2 |
| 1296 | ***Isca2*** | C1,Layer1/2,C0 |
| 1297 | ***Iscu*** | Dendritic,Layer6,C1,Layer1/2,C4,C0,Layer5,Layer3/4 |
| 1298 | ***Itch*** | C0 |
| 1299 | ***Itfg1*** | C1,Layer1/2,C0,Layer5,Layer3/4 |
| 1300 | ***Itga4*** | Layer1/2 |
| 1301 | ***Itm2b*** | Layer6,C1,Layer1/2,C0,Layer5,Layer3/4 |
| 1302 | ***Itpa*** | Layer1/2 |
| 1303 | ***Itpka*** | C1,Layer1/2 |
| 1304 | ***Itpr1*** | Layer1/2,C2,C0,Layer5 |
| 1305 | ***Itsn1*** | C0 |
| 1306 | ***Jagn1*** | Layer1/2 |
| 1307 | ***Jak1*** | C0,C5 |
| 1308 | ***Jak2*** | Layer1/2 |
| 1309 | ***Jakmip1*** | Layer1/2 |
| 1310 | ***Jkamp*** | C1 |
| 1311 | ***Jph3*** | Layer1/2 |
| 1312 | ***Jph4*** | Layer1/2 |
| 1313 | ***Jtb*** | Layer1/2,C0 |
| 1314 | ***Jun*** | Layer1/2 |
| 1315 | ***Kalrn*** | C1 |
| 1316 | ***Kansl1*** | Layer1/2 |
| 1317 | ***Kansl2*** | C0 |
| 1318 | ***Kat5*** | Layer1/2,C0 |
| 1319 | ***Kbtbd2*** | C1,C2,C0 |
| 1320 | ***Kcnab1*** | C1 |
| 1321 | ***Kcnab3*** | C0 |
| 1322 | ***Kcnb1*** | C1,Layer1/2 |
| 1323 | ***Kcnc2*** | C1,C0 |
| 1324 | ***Kcnd3*** | C1,C0 |
| 1325 | ***Kcnf1*** | Layer1/2,C0,Layer5 |
| 1326 | ***Kcng2*** | Layer1/2 |
| 1327 | ***Kcnh3*** | Layer1/2 |
| 1328 | ***Kcnip1*** | Dendritic |
| 1329 | ***Kcnip2*** | Layer1/2 |
| 1330 | ***Kcnip3*** | C1,C5,Layer3/4 |
| 1331 | ***Kcnip4*** | C1 |
| 1332 | ***Kcnj3*** | C1 |
| 1333 | ***Kcnj4*** | Layer1/2 |
| 1334 | ***Kcnk1*** | C1 |
| 1335 | ***Kcnma1*** | C0 |
| 1336 | ***Kcnmb4*** | Layer6,Layer1/2,C0,Layer3/4 |
| 1337 | ***Kcnn2*** | C0 |
| 1338 | ***Kcnq2*** | C0 |
| 1339 | ***Kcnq3*** | C1 |
| 1340 | ***Kcnq5*** | Layer1/2 |
| 1341 | ***Kcnt1*** | Layer1/2,C0 |
| 1342 | ***Kcnv1*** | Layer1/2 |
| 1343 | ***Kctd1*** | Layer1/2 |
| 1344 | ***Kctd13*** | C1 |
| 1345 | ***Kctd17*** | C1,C2 |
| 1346 | ***Kctd2*** | C1 |
| 1347 | ***Kctd3*** | C0 |
| 1348 | ***Kctd4*** | Dendritic,C3,Layer1/2 |
| 1349 | ***Kdm3a*** | Layer1/2 |
| 1350 | ***Keap1*** | C0 |
| 1351 | ***Kidins220*** | C1 |
| 1352 | ***Kif1bp*** | C1,Layer1/2,C0,C5,Layer3/4 |
| 1353 | ***Kif3c*** | C0 |
| 1354 | ***Kif5a*** | Layer6,C3,Layer1/2,Layer3/4 |
| 1355 | ***Kif5b*** | C0 |
| 1356 | ***Kif5c*** | C2,C0 |
| 1357 | ***Kifap3*** | Dendritic,Layer6,C1,Layer1/2,C0,Layer5,Layer3/4 |
| 1358 | ***Kifc2*** | Layer6,C1,C2,C0 |
| 1359 | ***Klc1*** | Dendritic,C1,Layer1/2,C2,C0,C5,Layer3/4 |
| 1360 | ***Klf9*** | Dendritic,Layer6,C1,C3,Layer1/2,C0 |
| 1361 | ***Klhdc2*** | C1 |
| 1362 | ***Klhdc3*** | C1,Layer1/2 |
| 1363 | ***Klhl2*** | Dendritic,Somatic,Layer1/2,C0,C5 |
| 1364 | ***Klhl23*** | Layer1/2 |
| 1365 | ***Klhl26*** | Layer1/2,C0 |
| 1366 | ***Klk8*** | Layer1/2 |
| 1367 | ***Kmt5b*** | Layer1/2 |
| 1368 | ***Kpna1*** | C1 |
| 1369 | ***Kpnb1*** | C1 |
| 1370 | ***Kras*** | Layer1/2 |
| 1371 | ***Krt10*** | Layer1/2,Layer3/4 |
| 1372 | ***Krtcap2*** | C1,Layer1/2,C0 |
| 1373 | ***Kxd1*** | C0 |
| 1374 | ***Lamp1*** | C1,C0 |
| 1375 | ***Lamtor1*** | C1 |
| 1376 | ***Lamtor2*** | Layer6,C1,C3,C0 |
| 1377 | ***Lamtor4*** | Layer6,C1,Layer1/2,C0 |
| 1378 | ***Lamtor5*** | C1,Layer1/2 |
| 1379 | ***Lancl1*** | C1,Layer1/2,C0,C5,Layer3/4 |
| 1380 | ***Large1*** | Layer1/2 |
| 1381 | ***Lcmt1*** | C1 |
| 1382 | ***Ldha*** | Layer1/2,C5 |
| 1383 | ***Ldhb*** | Layer6,C1,Layer1/2,C2,C0 |
| 1384 | ***Lef1*** | C1 |
| 1385 | ***Lemd3*** | C3 |
| 1386 | ***Leprotl1*** | C1 |
| 1387 | ***Letm1*** | C1 |
| 1388 | ***Letmd1*** | Layer1/2 |
| 1389 | ***Lgalsl*** | C0 |
| 1390 | ***Lgi1*** | Dendritic |
| 1391 | ***Lhfp*** | C1 |
| 1392 | ***Lhx2*** | Layer3/4 |
| 1393 | ***Lias*** | C1,Layer1/2 |
| 1394 | ***Lifr*** | Layer1/2 |
| 1395 | ***Limd2*** | Dendritic,C3,Layer1/2 |
| 1396 | ***Lin7b*** | C1,Layer1/2,C0 |
| 1397 | ***Lin7c*** | Layer1/2 |
| 1398 | ***Lingo1*** | C1,Layer1/2,C5,Layer3/4 |
| 1399 | ***Lipt2*** | Layer1/2 |
| 1400 | ***Llph*** | C1,C0 |
| 1401 | ***Lmbrd2*** | C1 |
| 1402 | ***Lmo1*** | Layer1/2 |
| 1403 | ***Lmo3*** | C0,C5 |
| 1404 | ***Lmo4*** | C1 |
| 1405 | ***Lmo7*** | Layer1/2 |
| 1406 | ***Lmtk2*** | Layer1/2 |
| 1407 | ***Lonp2*** | C0 |
| 1408 | ***Lratd1*** | Layer1/2 |
| 1409 | ***Lrba*** | Layer1/2 |
| 1410 | ***Lrfn2*** | Layer1/2 |
| 1411 | ***Lrfn4*** | Layer1/2 |
| 1412 | ***Lrfn5*** | C0 |
| 1413 | ***Lrp11*** | C1,Layer1/2 |
| 1414 | ***Lrrc28*** | Layer1/2 |
| 1415 | ***Lrrc45*** | Layer1/2 |
| 1416 | ***Lrrc49*** | C1 |
| 1417 | ***Lrrc4b*** | C1,C2 |
| 1418 | ***Lrrc57*** | Layer1/2 |
| 1419 | ***Lrrfip1*** | Layer1/2 |
| 1420 | ***Lsamp*** | C1,C0,C5 |
| 1421 | ***Lsm4*** | Layer6,C1,C0 |
| 1422 | ***Lsm6*** | C1,C0 |
| 1423 | ***Lsm7*** | Layer6,C1,C3,Layer1/2,C0,Layer5 |
| 1424 | ***Ltk*** | Layer1/2 |
| 1425 | ***Ltn1*** | Layer1/2 |
| 1426 | ***Luzp1*** | C0 |
| 1427 | ***Ly6a*** | Dendritic,C1,C2,C0 |
| 1428 | ***Ly6c1*** | Dendritic,Layer6,C1,C2,Layer5 |
| 1429 | ***Ly6h*** | Dendritic,Layer6,Layer1/2,C0,C5,Layer5 |
| 1430 | ***Lypd1*** | C3 |
| 1431 | ***Lyrm2*** | Layer1/2 |
| 1432 | ***Lysmd2*** | Layer6,C1,C0 |
| 1433 | ***Lyst*** | Layer1/2 |
| 1434 | ***Lztfl1*** | Layer1/2 |
| 1435 | ***Lztr1*** | C1,Layer1/2,C0 |
| 1436 | ***Lzts3*** | Layer1/2,C0 |
| 1437 | ***Macf1*** | C2 |
| 1438 | ***Madd*** | C1,Layer1/2 |
| 1439 | ***Maf1*** | C1,Layer5 |
| 1440 | ***Maged2*** | C0 |
| 1441 | ***Magee1*** | C0 |
| 1442 | ***Magi2*** | Layer1/2,C0 |
| 1443 | ***Magoh*** | C0 |
| 1444 | ***Magohb*** | Layer1/2 |
| 1445 | ***Mal2*** | Layer1/2,C5 |
| 1446 | ***Malat1*** | C3 |
| 1447 | ***Man1a2*** | Layer1/2 |
| 1448 | ***Manbal*** | Dendritic,Layer1/2 |
| 1449 | ***Maneal*** | C0 |
| 1450 | ***Map11*** | C0 |
| 1451 | ***Map1b*** | C1 |
| 1452 | ***Map1lc3a*** | C1 |
| 1453 | ***Map1lc3b*** | C1,Layer1/2,C0,Layer5 |
| 1454 | ***Map2k1*** | Dendritic,Layer6,C3,Layer1/2,C5,Layer3/4 |
| 1455 | ***Map2k2*** | C1 |
| 1456 | ***Map2k5*** | C0 |
| 1457 | ***Map3k12*** | Layer1/2 |
| 1458 | ***Map3k5*** | Layer1/2 |
| 1459 | ***Map3k6*** | Layer1/2 |
| 1460 | ***Map4*** | C1 |
| 1461 | ***Map4k3*** | Layer1/2,C0 |
| 1462 | ***Map7d2*** | C1,C0 |
| 1463 | ***Map9*** | Layer1/2 |
| 1464 | ***Mapk1*** | Dendritic,C1,C3,Layer1/2,C0,C5,Layer3/4 |
| 1465 | ***Mapk10*** | C1,Layer1/2,C2,Layer3/4 |
| 1466 | ***Mapk3*** | C1 |
| 1467 | ***Mapk8ip2*** | C1,C0 |
| 1468 | ***Mapk8ip3*** | Layer6,C1,Layer1/2,C2,C4,C0,Layer5 |
| 1469 | ***Mapk9*** | C0 |
| 1470 | ***Mapkbp1*** | Layer1/2 |
| 1471 | ***Mapre2*** | Dendritic,C1,Layer1/2,C0 |
| 1472 | ***Mapre3*** | C1,Layer1/2 |
| 1473 | ***Marc2*** | Layer1/2,C0 |
| 1474 | ***March2*** | C0 |
| 1475 | ***March5*** | C1 |
| 1476 | ***March6*** | Layer1/2 |
| 1477 | ***Mark4*** | C1 |
| 1478 | ***Mast1*** | C0 |
| 1479 | ***Mast2*** | C1 |
| 1480 | ***Mast3*** | C1,Layer1/2,C5 |
| 1481 | ***Mat2b*** | Layer6,C1,Layer1/2 |
| 1482 | ***Matk*** | Dendritic,Layer1/2,C0 |
| 1483 | ***Matr3*** | Dendritic,Somatic,C1,C3,Layer1/2,C2,Layer5,Layer3/4 |
| 1484 | ***Mau2*** | Layer1/2 |
| 1485 | ***Maz*** | C1,Layer1/2,C0 |
| 1486 | ***Mbd3*** | C1 |
| 1487 | ***Mbip*** | Layer1/2 |
| 1488 | ***Mbp*** | C0 |
| 1489 | ***Mcee*** | Layer1/2,C0 |
| 1490 | ***Mcfd2*** | Layer1/2 |
| 1491 | ***Mcm3ap*** | Layer1/2 |
| 1492 | ***Mcrs1*** | C1,C0 |
| 1493 | ***Mcts1*** | C1 |
| 1494 | ***Mdh1*** | Dendritic,Layer6,C1,Layer1/2,C2,C0,C5,Layer5,Layer3/4 |
| 1495 | ***Mdh2*** | C1 |
| 1496 | ***Mdp1*** | C1,Layer1/2 |
| 1497 | ***Me3*** | C0 |
| 1498 | ***Mea1*** | C1,C0 |
| 1499 | ***Mecp2*** | C0 |
| 1500 | ***Med10*** | Layer1/2 |
| 1501 | ***Med15*** | Layer1/2 |
| 1502 | ***Med16*** | Layer1/2 |
| 1503 | ***Med21*** | C0 |
| 1504 | ***Med22*** | Layer1/2,C0 |
| 1505 | ***Med24*** | C1,Layer1/2,C0 |
| 1506 | ***Med27*** | Layer1/2 |
| 1507 | ***Med28*** | Layer6,C1,Layer1/2,C0,Layer3/4 |
| 1508 | ***Med29*** | C1 |
| 1509 | ***Med30*** | C0 |
| 1510 | ***Med9*** | C0 |
| 1511 | ***Mef2c*** | Layer6,C1 |
| 1512 | ***Mef2d*** | Layer1/2 |
| 1513 | ***Meg3*** | Layer1/2,Layer5,Layer3/4 |
| 1514 | ***Megf9*** | C1,C0 |
| 1515 | ***Mest*** | Layer1/2 |
| 1516 | ***Metap1d*** | Layer1/2 |
| 1517 | ***Mettl22*** | Layer1/2 |
| 1518 | ***Mettl25*** | Layer1/2 |
| 1519 | ***Mfap1b*** | C0 |
| 1520 | ***Mff*** | Dendritic,Somatic,Layer6,C1,Layer1/2,C0 |
| 1521 | ***Mfn2*** | C1 |
| 1522 | ***Mfsd10*** | Layer1/2 |
| 1523 | ***Mfsd4a*** | Layer1/2 |
| 1524 | ***Mgat3*** | Layer1/2,C2 |
| 1525 | ***Mgat4a*** | Layer1/2 |
| 1526 | ***Mgrn1*** | C1,Layer1/2,Layer5,Layer3/4 |
| 1527 | ***Mgst1*** | C0 |
| 1528 | ***Mgst3*** | Layer6,C1,Layer1/2,C0,Layer5,Layer3/4 |
| 1529 | ***Mia3*** | C1 |
| 1530 | ***Mical2*** | Layer1/2 |
| 1531 | ***Micos10*** | Dendritic,Layer6,C1,Layer1/2,C0,C5,Layer3/4 |
| 1532 | ***Micos13*** | Dendritic,Layer6,C1,Layer1/2,C0,Layer5 |
| 1533 | ***Micu1*** | C1,Layer1/2,C0 |
| 1534 | ***Mien1*** | Layer6,C1,Layer1/2,C0 |
| 1535 | ***Mif*** | Somatic,Layer6,C1,Layer1/2,C4,C0,Layer5,Layer3/4 |
| 1536 | ***Miga2*** | C1 |
| 1537 | ***Mindy3*** | Layer1/2,Layer3/4 |
| 1538 | ***Mink1*** | C1,Layer1/2,C5 |
| 1539 | ***Mipep*** | Layer1/2 |
| 1540 | ***Mirg*** | Layer1/2 |
| 1541 | ***Mkrn1*** | C1,Layer1/2 |
| 1542 | ***Mkx*** | Layer1/2 |
| 1543 | ***Mlf2*** | Layer6,C1,Layer1/2,C2,C0,C5 |
| 1544 | ***Mllt11*** | Layer6,C1,Layer1/2 |
| 1545 | ***Mmd*** | Layer6,C1,Layer1/2 |
| 1546 | ***Mmd2*** | Dendritic,Layer1/2 |
| 1547 | ***Mmp17*** | Dendritic |
| 1548 | ***Mmp24*** | Layer1/2 |
| 1549 | ***Mn1*** | Layer1/2 |
| 1550 | ***Mob2*** | C1 |
| 1551 | ***Mob4*** | C1 |
| 1552 | ***Mocs2*** | C1,C0 |
| 1553 | ***Morf4l1*** | C1,C2 |
| 1554 | ***Morf4l2*** | Layer1/2,Layer5 |
| 1555 | ***Mpc1*** | Dendritic,Layer6,C1,C3,Layer1/2,C2,C0,C5,Layer5,Layer3/4 |
| 1556 | ***Mpc2*** | Dendritic,Layer6,C1,C3,Layer1/2,C0,C5,Layer5 |
| 1557 | ***Mpdu1*** | C1,C3 |
| 1558 | ***Mpi*** | C1,Layer1/2,C0 |
| 1559 | ***Mppe1*** | Layer1/2 |
| 1560 | ***Mprip*** | C1,Layer1/2,C2 |
| 1561 | ***Mpv17*** | C0 |
| 1562 | ***Mras*** | C1,Layer1/2 |
| 1563 | ***Mrfap1*** | Dendritic,Layer6,C1,C3,Layer1/2,C0,C5,Layer5,Layer3/4 |
| 1564 | ***Mroh1*** | C1 |
| 1565 | ***Mrpl10*** | C1,C0 |
| 1566 | ***Mrpl11*** | C1 |
| 1567 | ***Mrpl12*** | C1 |
| 1568 | ***Mrpl13*** | C1,Layer1/2,C0 |
| 1569 | ***Mrpl14*** | Layer6,C1,Layer1/2 |
| 1570 | ***Mrpl15*** | C1 |
| 1571 | ***Mrpl17*** | C1 |
| 1572 | ***Mrpl18*** | Layer6,C1 |
| 1573 | ***Mrpl20*** | Dendritic,Layer6,C1,Layer1/2,C0,Layer5,Layer3/4 |
| 1574 | ***Mrpl21*** | C1 |
| 1575 | ***Mrpl23*** | Layer6,C1,Layer1/2,C0,Layer5 |
| 1576 | ***Mrpl27*** | C1,C3,Layer1/2,C0 |
| 1577 | ***Mrpl3*** | Layer1/2 |
| 1578 | ***Mrpl30*** | C1,Layer1/2 |
| 1579 | ***Mrpl32*** | C0 |
| 1580 | ***Mrpl33*** | Dendritic,Somatic,Layer6,C1,C3,Layer1/2,C0,Layer5 |
| 1581 | ***Mrpl34*** | C1,C0 |
| 1582 | ***Mrpl36*** | C1,Layer1/2,C0 |
| 1583 | ***Mrpl4*** | C1,C0 |
| 1584 | ***Mrpl40*** | C1,C0 |
| 1585 | ***Mrpl41*** | Dendritic,Layer6,C1,Layer1/2,C2,C0,Layer5,Layer3/4 |
| 1586 | ***Mrpl42*** | C1,Layer1/2 |
| 1587 | ***Mrpl43*** | C1 |
| 1588 | ***Mrpl45*** | C1 |
| 1589 | ***Mrpl46*** | C1 |
| 1590 | ***Mrpl48*** | C1,C0 |
| 1591 | ***Mrpl51*** | Dendritic,C1,C0 |
| 1592 | ***Mrpl52*** | Dendritic,C1,C0 |
| 1593 | ***Mrpl53*** | Layer6,C1,Layer1/2,C0,Layer5 |
| 1594 | ***Mrpl54*** | C1,Layer1/2,C0 |
| 1595 | ***Mrpl55*** | C1 |
| 1596 | ***Mrpl57*** | Layer6,C1,Layer1/2,C0,Layer5 |
| 1597 | ***Mrpl58*** | Layer6,C1,Layer5 |
| 1598 | ***Mrpl9*** | C1 |
| 1599 | ***Mrps10*** | C1 |
| 1600 | ***Mrps12*** | Layer6,C1,Layer1/2,C0,Layer3/4 |
| 1601 | ***Mrps14*** | C1,Layer1/2,C0 |
| 1602 | ***Mrps15*** | C1,C0 |
| 1603 | ***Mrps16*** | C1,C0 |
| 1604 | ***Mrps17*** | C0 |
| 1605 | ***Mrps18a*** | C1 |
| 1606 | ***Mrps18b*** | C1,C0 |
| 1607 | ***Mrps18c*** | Layer6,C0 |
| 1608 | ***Mrps21*** | Layer6,C1,Layer1/2,C0,C5,Layer5,Layer3/4 |
| 1609 | ***Mrps23*** | C1 |
| 1610 | ***Mrps24*** | C1,C0 |
| 1611 | ***Mrps25*** | C1,C0 |
| 1612 | ***Mrps26*** | C1,Layer1/2 |
| 1613 | ***Mrps30*** | Layer1/2 |
| 1614 | ***Mrps33*** | Dendritic,Somatic,Layer6,C1,Layer1/2,C0,C5,Layer5,Layer3/4 |
| 1615 | ***Mrps34*** | C1 |
| 1616 | ***Mrps36*** | Layer6,C3,Layer1/2,C0,Layer5 |
| 1617 | ***Mrps5*** | C1 |
| 1618 | ***Mrps7*** | C1 |
| 1619 | ***Mrtfa*** | Layer1/2 |
| 1620 | ***Msantd4*** | C1,C0 |
| 1621 | ***Msra*** | Layer5,Layer3/4 |
| 1622 | ***Mt1*** | C5 |
| 1623 | ***Mt3*** | Layer1/2,C5 |
| 1624 | ***Mtch1*** | C1 |
| 1625 | ***Mtch2*** | C1 |
| 1626 | ***Mtfp1*** | C1,Layer1/2,C0 |
| 1627 | ***Mtg1*** | Layer1/2 |
| 1628 | ***Mthfsl*** | Layer1/2 |
| 1629 | ***Mtln*** | Dendritic,C1,Layer1/2,C0 |
| 1630 | ***Mtmr12*** | Layer1/2 |
| 1631 | ***Mtor*** | C1,C0 |
| 1632 | ***Mtpap*** | Layer1/2 |
| 1633 | ***Mtpn*** | Layer1/2 |
| 1634 | ***Mttp*** | Layer1/2 |
| 1635 | ***Mul1*** | C1 |
| 1636 | ***Mycbp*** | Layer1/2 |
| 1637 | ***Mycbp2*** | C1,C0,Layer5 |
| 1638 | ***Mydgf*** | C1 |
| 1639 | ***Myh10*** | C1 |
| 1640 | ***Myh14*** | Layer1/2 |
| 1641 | ***Myl12b*** | Layer6,C1,Layer1/2,C0,Layer5,Layer3/4 |
| 1642 | ***Myl6*** | C1,C5 |
| 1643 | ***Myo5a*** | Dendritic,C1 |
| 1644 | ***Myo5b*** | Layer1/2 |
| 1645 | ***Myt1l*** | C1 |
| 1646 | ***Mzt1*** | Dendritic,C1,C3,Layer1/2 |
| 1647 | ***Mzt2*** | C1 |
| 1648 | ***N4bp2l1*** | Layer1/2 |
| 1649 | ***Naa20*** | Layer6,C1,Layer1/2 |
| 1650 | ***Naa25*** | Layer1/2 |
| 1651 | ***Naa30*** | Layer1/2 |
| 1652 | ***Naa38*** | Dendritic,Somatic,Layer6,C1,Layer1/2,C0,Layer5,Layer3/4 |
| 1653 | ***Naa60*** | C1,Layer1/2,C0 |
| 1654 | ***Naaa*** | C0 |
| 1655 | ***Nabp2*** | C1 |
| 1656 | ***Naca*** | Dendritic,Somatic,Layer6,C1,Layer1/2,C4,C0,Layer5,Layer3/4 |
| 1657 | ***Nap1l1*** | C1,Layer1/2 |
| 1658 | ***Nap1l2*** | C1,C0 |
| 1659 | ***Nap1l4*** | C1 |
| 1660 | ***Nap1l5*** | Dendritic,C0,Layer5 |
| 1661 | ***Napa*** | C1,Layer1/2 |
| 1662 | ***Napb*** | Dendritic,C1,Layer1/2,C2,C0,Layer3/4 |
| 1663 | ***Napg*** | C1,C0 |
| 1664 | ***Nars*** | Dendritic,C1,Layer1/2,C0 |
| 1665 | ***Nat14*** | Layer1/2,C0 |
| 1666 | ***Naxd*** | C1 |
| 1667 | ***Naxe*** | C1 |
| 1668 | ***Nbas*** | C0 |
| 1669 | ***Nbea*** | C1 |
| 1670 | ***Nbr1*** | C1,Layer1/2,C0 |
| 1671 | ***Ncald*** | Dendritic,Layer6,Layer1/2,C2,C4,C0,Layer5,Layer3/4 |
| 1672 | ***Ncaph2*** | C1,C0 |
| 1673 | ***Ncdn*** | C1,Layer1/2,C2,C5 |
| 1674 | ***Nckap1*** | C1,C3,Layer1/2,C2,C0,C5,Layer5,Layer3/4 |
| 1675 | ***Ncoa1*** | C1,C5 |
| 1676 | ***Ncoa2*** | Layer1/2,C0 |
| 1677 | ***Ncoa7*** | C0 |
| 1678 | ***Ncor2*** | Layer1/2 |
| 1679 | ***Ncs1*** | Dendritic,C1,Layer1/2,C2,C0 |
| 1680 | ***Ncstn*** | Layer1/2 |
| 1681 | ***Ndel1*** | C1 |
| 1682 | ***Ndfip1*** | Dendritic,Somatic,Layer6,C1,C3,Layer1/2,C2,C4,C0,Layer5,Layer3/4 |
| 1683 | ***Ndfip2*** | C1 |
| 1684 | ***Ndn*** | Layer5 |
| 1685 | ***Ndrg1*** | C0 |
| 1686 | ***Ndrg2*** | Dendritic,C1,C2,C0 |
| 1687 | ***Ndrg3*** | C1,C3,Layer1/2,C0,C5,Layer5 |
| 1688 | ***Ndrg4*** | Dendritic,Somatic,Layer6,C1,C3,Layer1/2,C2,C0,C5,Layer5,Layer3/4 |
| 1689 | ***Ndufa1*** | Somatic,C1,C0 |
| 1690 | ***Ndufa10*** | C1 |
| 1691 | ***Ndufa11*** | Dendritic,Layer6,C1,Layer1/2,C2,C4,Layer5 |
| 1692 | ***Ndufa12*** | Dendritic,Somatic,Layer6,C1,C3,Layer1/2,C0,C5,Layer5,Layer3/4 |
| 1693 | ***Ndufa13*** | Dendritic,Layer6,C1,C3,Layer1/2,C2,C4,C0,C5,Layer5,Layer3/4 |
| 1694 | ***Ndufa2*** | Dendritic,Layer6,C1,C3,Layer1/2,C4,C0 |
| 1695 | ***Ndufa3*** | C1,C0 |
| 1696 | ***Ndufa4*** | Dendritic,Somatic,Layer6,C1,C3,Layer1/2,C4,C0,C5,Layer5,Layer3/4 |
| 1697 | ***Ndufa5*** | Dendritic,Layer6,C1,C3,Layer1/2,C2,C4,C0,C5,Layer5,Layer3/4 |
| 1698 | ***Ndufa6*** | Dendritic,Somatic,Layer6,C1,C3,Layer1/2,C2,C4,C0,Layer5,Layer3/4 |
| 1699 | ***Ndufa7*** | Dendritic,Somatic,Layer6,C1,C3,Layer1/2,C4,C0,C5,Layer5,Layer3/4 |
| 1700 | ***Ndufa8*** | Dendritic,Layer6,C1,Layer1/2,C0 |
| 1701 | ***Ndufa9*** | Dendritic,Layer6,C1,C0,C5 |
| 1702 | ***Ndufab1*** | Dendritic,Layer6,C1,C3,Layer1/2,C4,C0,C5,Layer5,Layer3/4 |
| 1703 | ***Ndufaf1*** | C0 |
| 1704 | ***Ndufaf2*** | C1 |
| 1705 | ***Ndufaf3*** | Layer6,C1,C0,Layer5 |
| 1706 | ***Ndufaf5*** | C1,C0 |
| 1707 | ***Ndufaf8*** | C1,Layer1/2,C0 |
| 1708 | ***Ndufb10*** | Dendritic,Layer6,C1,C0 |
| 1709 | ***Ndufb11*** | Dendritic,Layer6,C1,Layer1/2,C0,Layer5,Layer3/4 |
| 1710 | ***Ndufb1-ps*** | Dendritic,Layer6,C1,Layer1/2,C2,C0,C5,Layer5,Layer3/4 |
| 1711 | ***Ndufb2*** | Dendritic,Somatic,Layer6,C1,C3,Layer1/2,C2,C0,C5,Layer5,Layer3/4 |
| 1712 | ***Ndufb3*** | Dendritic,Layer6,C1,C3,Layer1/2,C2,C4,C0,C5,Layer5,Layer3/4 |
| 1713 | ***Ndufb4*** | Dendritic,Somatic,Layer6,C1,C3,Layer1/2,C2,C4,C0,C5,Layer5,Layer3/4 |
| 1714 | ***Ndufb5*** | Dendritic,Layer6,C1,C3,Layer1/2,C2,C4,C0,Layer5 |
| 1715 | ***Ndufb6*** | Dendritic,Layer6,C1,Layer1/2,C4,C0,C5,Layer5,Layer3/4 |
| 1716 | ***Ndufb7*** | C1,C0,Layer5 |
| 1717 | ***Ndufb8*** | Dendritic,Layer6,C1,Layer1/2,C2,C4,C0,Layer5,Layer3/4 |
| 1718 | ***Ndufb9*** | Dendritic,Somatic,Layer6,C1,C3,Layer1/2,C4,C0,C5,Layer5,Layer3/4 |
| 1719 | ***Ndufc1*** | Dendritic,Somatic,Layer6,C1,C3,Layer1/2,C2,C0,C5,Layer5 |
| 1720 | ***Ndufc2*** | Dendritic,Somatic,Layer6,C1,C3,Layer1/2,C2,C0,Layer5,Layer3/4 |
| 1721 | ***Ndufs1*** | Dendritic,C1 |
| 1722 | ***Ndufs2*** | Dendritic,Layer6,C1,Layer1/2,C2,C0,C5 |
| 1723 | ***Ndufs3*** | C1,Layer1/2,C0,C5 |
| 1724 | ***Ndufs4*** | Dendritic,Layer6,C1,Layer1/2,C0,Layer3/4 |
| 1725 | ***Ndufs5*** | Dendritic,Somatic,Layer6,C1,C3,Layer1/2,C2,C4,C0,C5,Layer5,Layer3/4 |
| 1726 | ***Ndufs6*** | C1 |
| 1727 | ***Ndufs7*** | Layer6,C1,C3,Layer1/2,C0,Layer5 |
| 1728 | ***Ndufs8*** | Dendritic,Layer6,C1,Layer1/2,C0,Layer5 |
| 1729 | ***Ndufv2*** | Dendritic,Layer6,C1,C3,Layer1/2,C2,C4,C0,C5,Layer5,Layer3/4 |
| 1730 | ***Ndufv3*** | Somatic,Layer6,C1,Layer1/2,C0,C5,Layer5 |
| 1731 | ***Necab1*** | Dendritic,Layer5 |
| 1732 | ***Necab2*** | Layer1/2,C5 |
| 1733 | ***Necab3*** | Layer1/2,C0 |
| 1734 | ***Necap1*** | Layer6,C1,Layer1/2,C2,C0,Layer5,Layer3/4 |
| 1735 | ***Nectin1*** | Layer1/2 |
| 1736 | ***Nedd4*** | Dendritic,Layer6,Layer1/2,Layer5 |
| 1737 | ***Nedd4l*** | Layer1/2,C0,C5,Layer3/4 |
| 1738 | ***Nedd8*** | Dendritic,Somatic,Layer6,C1,C3,Layer1/2,C0,Layer5,Layer3/4 |
| 1739 | ***Nefl*** | Dendritic,Somatic,Layer6,C3,Layer1/2,C0,Layer3/4 |
| 1740 | ***Negr1*** | C3 |
| 1741 | ***Nek1*** | Layer1/2 |
| 1742 | ***Nelfa*** | Layer5 |
| 1743 | ***Nell2*** | Layer1/2,C2,C5 |
| 1744 | ***Nenf*** | Dendritic,Layer6,C1,C0,Layer5 |
| 1745 | ***Neo1*** | C1 |
| 1746 | ***Neto1*** | C0 |
| 1747 | ***Neu1*** | Layer1/2 |
| 1748 | ***Neurl1a*** | Dendritic,Layer1/2,C0 |
| 1749 | ***Neurl4*** | C1 |
| 1750 | ***Neurod1*** | Layer1/2 |
| 1751 | ***Neurod2*** | C1,Layer1/2,C0 |
| 1752 | ***Nfe2l1*** | C1 |
| 1753 | ***Nfib*** | Dendritic |
| 1754 | ***Nfkbia*** | C1,Layer1/2,C0 |
| 1755 | ***Nfs1*** | Layer1/2,C0 |
| 1756 | ***Ngef*** | Layer1/2,C0 |
| 1757 | ***Nhlrc3*** | Layer1/2 |
| 1758 | ***Nhp2*** | C1 |
| 1759 | ***Nicn1*** | Dendritic,C1,Layer1/2 |
| 1760 | ***Nipsnap1*** | C1,C0 |
| 1761 | ***Nipsnap2*** | C0 |
| 1762 | ***Nisch*** | Dendritic,Layer6,C1,Layer1/2,C0,Layer3/4 |
| 1763 | ***Nkain4*** | C0 |
| 1764 | ***Nlk*** | C1,Layer1/2 |
| 1765 | ***Nln*** | Layer1/2 |
| 1766 | ***Nmd3*** | Layer1/2 |
| 1767 | ***Nme1*** | Dendritic,Layer6,C1,Layer1/2,C4,C0,C5 |
| 1768 | ***Nme2*** | C5 |
| 1769 | ***Nme5*** | Layer6,Layer1/2 |
| 1770 | ***Nme7*** | Layer1/2 |
| 1771 | ***Nmt1*** | C1,Layer1/2,C0 |
| 1772 | ***Noc2l*** | Layer1/2 |
| 1773 | ***Noct*** | C1,Layer1/2 |
| 1774 | ***Nol7*** | Dendritic,C1,C3,Layer1/2,C0,Layer5 |
| 1775 | ***Nol9*** | Layer1/2 |
| 1776 | ***Nono*** | C1 |
| 1777 | ***Nop10*** | Dendritic,Layer6,C1,C3,Layer1/2,C2,C0,Layer5,Layer3/4 |
| 1778 | ***Nop14*** | Layer1/2,Layer3/4 |
| 1779 | ***Nop16*** | Layer1/2 |
| 1780 | ***Npc1*** | C1 |
| 1781 | ***Npdc1*** | C1,Layer1/2,C0 |
| 1782 | ***Npm1*** | Dendritic,Layer6,C1,Layer1/2,C2,C0 |
| 1783 | ***Nptn*** | C1,C3,Layer1/2,C2,C5 |
| 1784 | ***Nptx1*** | C1,Layer1/2,C0 |
| 1785 | ***Nptxr*** | Layer1/2 |
| 1786 | ***Nr1d2*** | C1,C2 |
| 1787 | ***Nr1h2*** | C0 |
| 1788 | ***Nrbp1*** | C1 |
| 1789 | ***Nrbp2*** | C0 |
| 1790 | ***Nrcam*** | C1,Layer1/2 |
| 1791 | ***Nrd1*** | C1,Layer1/2,C0 |
| 1792 | ***Nrgn*** | C1,C3 |
| 1793 | ***Nrip1*** | Layer1/2 |
| 1794 | ***Nrip3*** | C1,C0 |
| 1795 | ***Nrn1*** | Dendritic,Layer6,C1,C3,Layer1/2,C2,C0,Layer5,Layer3/4 |
| 1796 | ***Nrsn1*** | Dendritic,Layer6,C1,C0,Layer5,Layer3/4 |
| 1797 | ***Nrxn1*** | Layer6,C1,C3,Layer1/2,C2,C0,C5,Layer3/4 |
| 1798 | ***Nrxn3*** | C1,C0 |
| 1799 | ***Nsa2*** | C0 |
| 1800 | ***Nsd2*** | Layer1/2,C0 |
| 1801 | ***Nsf*** | Dendritic,C1,Layer1/2,C2,C0,Layer3/4 |
| 1802 | ***Nsg1*** | Dendritic,Somatic,Layer6,C1,Layer1/2,C2,C0,Layer5,Layer3/4 |
| 1803 | ***Nsg2*** | Layer1/2,C2,Layer3/4 |
| 1804 | ***Nsmaf*** | Layer1/2 |
| 1805 | ***Nsmce3*** | Layer3/4 |
| 1806 | ***Nsmf*** | C1,C0 |
| 1807 | ***Nsun2*** | C1 |
| 1808 | ***Nt5c*** | C1 |
| 1809 | ***Nt5c2*** | Layer1/2 |
| 1810 | ***Nt5c3*** | C1,C0 |
| 1811 | ***Nt5dc3*** | C1 |
| 1812 | ***Nt5m*** | C1,Layer1/2,C0,C5 |
| 1813 | ***Ntan1*** | C0 |
| 1814 | ***Ntpcr*** | C0 |
| 1815 | ***Ntrk2*** | Layer1/2 |
| 1816 | ***Ntrk3*** | C1,Layer1/2,C0 |
| 1817 | ***Ntsr1*** | C1 |
| 1818 | ***Ntsr2*** | Dendritic |
| 1819 | ***Nudc*** | C1 |
| 1820 | ***Nudt16l1*** | Layer1/2 |
| 1821 | ***Nudt18*** | C1,C0 |
| 1822 | ***Nudt19*** | C1,C0,Layer5 |
| 1823 | ***Nudt3*** | C1,C3,Layer1/2,C0 |
| 1824 | ***Nudt9*** | C1,C0 |
| 1825 | ***Numa1*** | C0 |
| 1826 | ***Numbl*** | Layer1/2 |
| 1827 | ***Nup107*** | Layer1/2 |
| 1828 | ***Nup133*** | Layer1/2 |
| 1829 | ***Nup155*** | Layer1/2 |
| 1830 | ***Nup35*** | Layer1/2 |
| 1831 | ***Nvl*** | Layer1/2 |
| 1832 | ***Nxf1*** | Layer1/2 |
| 1833 | ***Nxt1*** | Layer1/2 |
| 1834 | ***Oaz1*** | Dendritic,Somatic,Layer6,C1,C3,Layer1/2,C4,C0,Layer5,Layer3/4 |
| 1835 | ***Oaz2*** | Layer1/2 |
| 1836 | ***Ociad1*** | Layer6,C1,C3,Layer1/2,C2,C0,Layer3/4 |
| 1837 | ***Ociad2*** | C1,C3,Layer1/2,C5 |
| 1838 | ***Ocrl*** | C0,Layer3/4 |
| 1839 | ***Odc1*** | C1,C0 |
| 1840 | ***Oga*** | Dendritic,C1,C3,Layer1/2,C0,Layer5,Layer3/4 |
| 1841 | ***Ogdh*** | C1,Layer1/2 |
| 1842 | ***Ogfrl1*** | C2 |
| 1843 | ***Ogt*** | Layer1/2,C0,Layer5 |
| 1844 | ***Oip5os1*** | Dendritic,Layer1/2,Layer5 |
| 1845 | ***Ola1*** | C1,C2 |
| 1846 | ***Olfm1*** | Dendritic,Layer6,C1,C3,Layer1/2,C2,C4,C0,Layer5,Layer3/4 |
| 1847 | ***Opa1*** | C1,Layer1/2 |
| 1848 | ***Ophn1*** | Layer1/2 |
| 1849 | ***Orc2*** | Layer1/2 |
| 1850 | ***Osbpl1a*** | C1 |
| 1851 | ***Ost4*** | Dendritic,Layer6,C1,C0,Layer5 |
| 1852 | ***Otub2*** | Layer1/2 |
| 1853 | ***Otud4*** | Layer1/2 |
| 1854 | ***Otud5*** | Layer1/2 |
| 1855 | ***Otud7a*** | C1 |
| 1856 | ***Ovol2*** | Layer1/2,Layer5 |
| 1857 | ***Oxct1*** | C1 |
| 1858 | ***Oxr1*** | C1,C0,Layer5,Layer3/4 |
| 1859 | ***P3h3*** | Layer1/2 |
| 1860 | ***P4htm*** | C1 |
| 1861 | ***Pafah1b1*** | Dendritic,C1,Layer3/4 |
| 1862 | ***Paics*** | C1,C0 |
| 1863 | ***Paip2*** | Dendritic,Layer6,C1,C0 |
| 1864 | ***Pak1*** | C1 |
| 1865 | ***Pak6*** | Layer1/2 |
| 1866 | ***Pakap.1*** | C1,C0 |
| 1867 | ***Pam16*** | Dendritic,Layer6,C1,Layer1/2,C0,C5,Layer5 |
| 1868 | ***Pantr1*** | Layer5 |
| 1869 | ***Parg*** | Layer1/2 |
| 1870 | ***Park7*** | C1,Layer1/2,C0 |
| 1871 | ***Parp6*** | C1,Layer1/2,C0 |
| 1872 | ***Parvb*** | Layer1/2 |
| 1873 | ***Paxip1*** | Layer1/2 |
| 1874 | ***Paxx*** | C1,C0,C5 |
| 1875 | ***Pcbp1*** | C1 |
| 1876 | ***Pcbp2*** | Dendritic,C1 |
| 1877 | ***Pcbp3*** | C1 |
| 1878 | ***Pcbp4*** | C1 |
| 1879 | ***Pcca*** | Layer1/2 |
| 1880 | ***Pccb*** | Layer1/2,C0 |
| 1881 | ***Pcdh7*** | C0 |
| 1882 | ***Pcdhb7*** | Layer1/2 |
| 1883 | ***Pcif1*** | C1,C0 |
| 1884 | ***Pcmt1*** | C1,Layer1/2 |
| 1885 | ***Pcmtd1*** | Layer1/2 |
| 1886 | ***Pcna*** | C3,Layer1/2 |
| 1887 | ***Pcnp*** | C0 |
| 1888 | ***Pcnx2*** | C1,C0 |
| 1889 | ***Pcp4*** | Layer6,C1,C0,C5 |
| 1890 | ***Pcp4l1*** | C1 |
| 1891 | ***Pcsk1n*** | Dendritic,Somatic,Layer6,Layer1/2,C0 |
| 1892 | ***Pcsk2*** | C1,Layer1/2,C0,Layer3/4 |
| 1893 | ***Pcsk7*** | Layer1/2 |
| 1894 | ***Pdcd10*** | C1 |
| 1895 | ***Pdcd5*** | Layer6,C1,Layer1/2,C0,Layer5 |
| 1896 | ***Pdcd6*** | Layer6,C1,Layer1/2,C5 |
| 1897 | ***Pde2a*** | Layer6,C1,Layer1/2,C0,Layer5,Layer3/4 |
| 1898 | ***Pde4b*** | Layer1/2 |
| 1899 | ***Pde4dip*** | Layer1/2 |
| 1900 | ***Pdgfb*** | Layer1/2 |
| 1901 | ***Pdha1*** | C1 |
| 1902 | ***Pdhb*** | C1,Layer1/2,C0 |
| 1903 | ***Pdk1*** | Layer1/2 |
| 1904 | ***Pdk2*** | Layer1/2 |
| 1905 | ***Pdlim7*** | Layer1/2 |
| 1906 | ***Pdp1*** | C1,Layer1/2,C2 |
| 1907 | ***Pds5a*** | Layer1/2 |
| 1908 | ***Pds5b*** | Layer1/2,C2 |
| 1909 | ***Pdxp*** | Dendritic,Layer6,C1,Layer1/2,C0,Layer5 |
| 1910 | ***Pdzd11*** | C1,Layer1/2,C0 |
| 1911 | ***Pea15a*** | C1,C3,C5 |
| 1912 | ***Peak1*** | Layer1/2 |
| 1913 | ***Pebp1*** | C1,Layer1/2,C0,C5 |
| 1914 | ***Pef1*** | C1,C2 |
| 1915 | ***Per3*** | Layer1/2,Layer3/4 |
| 1916 | ***Pet100*** | C1 |
| 1917 | ***Pex11b*** | C2 |
| 1918 | ***Pex16*** | Layer1/2 |
| 1919 | ***Pex19*** | C1,C0 |
| 1920 | ***Pex5l*** | C1,C0,C5 |
| 1921 | ***Pfdn1*** | Layer6,C1,C3,Layer1/2,C0,Layer5,Layer3/4 |
| 1922 | ***Pfdn2*** | Layer6,C1,Layer1/2,C0 |
| 1923 | ***Pfdn4*** | C1,C0 |
| 1924 | ***Pfdn5*** | Dendritic,Layer6,C1,C3,Layer1/2,C4,C0,Layer5 |
| 1925 | ***Pfdn6*** | C1,C0 |
| 1926 | ***Pfkfb2*** | Layer1/2 |
| 1927 | ***Pfkm*** | Dendritic,Layer1/2,C0,C5 |
| 1928 | ***Pfkp*** | C1,C5 |
| 1929 | ***Pfn2*** | Layer6,C1,C3,Layer1/2,C0,Layer5 |
| 1930 | ***Pgam1*** | Dendritic,Layer6,C1,Layer1/2,C2,C0,C5,Layer5,Layer3/4 |
| 1931 | ***Pgam5*** | Layer1/2 |
| 1932 | ***Pgbd5*** | Dendritic,C1,Layer1/2,C0 |
| 1933 | ***Pgk1*** | Dendritic,Layer6,C1,Layer1/2,C2,C0,Layer3/4 |
| 1934 | ***Pgm2l1*** | C1,Layer1/2,C0,Layer5 |
| 1935 | ***Pgrmc1*** | Dendritic,Layer6,Layer5 |
| 1936 | ***Phactr1*** | Layer1/2 |
| 1937 | ***Phactr3*** | C1,C0 |
| 1938 | ***Phax*** | C0 |
| 1939 | ***Phb2*** | C1,C0 |
| 1940 | ***Phf12*** | Layer1/2 |
| 1941 | ***Phf20*** | C1,C0 |
| 1942 | ***Phf20l1*** | Layer1/2,C0 |
| 1943 | ***Phf24*** | C1,Layer1/2,C0,Layer3/4 |
| 1944 | ***Phf5a*** | Dendritic,C1,C0 |
| 1945 | ***Phka2*** | Layer1/2 |
| 1946 | ***Phpt1*** | Dendritic,C1,C0 |
| 1947 | ***Phyh*** | C1,C0 |
| 1948 | ***Phyhipl*** | Dendritic,C1,Layer1/2 |
| 1949 | ***Pi4ka*** | C1,Layer1/2,Layer3/4 |
| 1950 | ***Pias3*** | Layer1/2 |
| 1951 | ***Pigh*** | Layer1/2 |
| 1952 | ***Pigp*** | C1,C0 |
| 1953 | ***Pigs*** | C1,Layer1/2,C0 |
| 1954 | ***Pigyl*** | C1 |
| 1955 | ***Pik3cb*** | Layer1/2 |
| 1956 | ***Pik3r4*** | Layer1/2 |
| 1957 | ***Pim2*** | Layer1/2 |
| 1958 | ***Pim3*** | C1,C0 |
| 1959 | ***Pin1*** | Layer6,C1,Layer1/2,Layer5 |
| 1960 | ***Pin4*** | C1 |
| 1961 | ***Pink1*** | Layer6,C1,C3,Layer1/2,C2,C0,Layer5,Layer3/4 |
| 1962 | ***Pip4p1*** | Layer6,C1 |
| 1963 | ***Pip5k1a*** | Layer1/2 |
| 1964 | ***Pitpna*** | C1,Layer1/2,C0 |
| 1965 | ***Pitpnm1*** | C1,Layer1/2 |
| 1966 | ***Pja1*** | C3,Layer1/2,Layer5 |
| 1967 | ***Pja2*** | Dendritic,C1,C3,Layer1/2,C0 |
| 1968 | ***Pkd1*** | Layer1/2 |
| 1969 | ***Pkig*** | C1,Layer1/2,C5,Layer5 |
| 1970 | ***Pkm*** | C1 |
| 1971 | ***Pknox2*** | Layer1/2 |
| 1972 | ***Pkp2*** | Layer1/2 |
| 1973 | ***Plaat1*** | Layer1/2 |
| 1974 | ***Plcb4*** | C1,C0 |
| 1975 | ***Plcxd2*** | C1,C0 |
| 1976 | ***Pld3*** | Layer1/2 |
| 1977 | ***Plec*** | Layer1/2 |
| 1978 | ***Plekhb2*** | C1,C2,C0 |
| 1979 | ***Plekhg5*** | Layer1/2 |
| 1980 | ***Plekhj1*** | C1,Layer1/2 |
| 1981 | ***Plk2*** | Layer6,Layer1/2,Layer3/4 |
| 1982 | ***Plpbp*** | C1 |
| 1983 | ***Plpp3*** | C0 |
| 1984 | ***Plpp5*** | Layer1/2 |
| 1985 | ***Plpp6*** | Layer1/2,Layer5 |
| 1986 | ***Plppr2*** | Layer1/2 |
| 1987 | ***Pls3*** | C1,Layer1/2 |
| 1988 | ***Plxna2*** | Layer1/2 |
| 1989 | ***Plxnd1*** | Layer1/2 |
| 1990 | ***Pmm1*** | Dendritic,Layer6,C1,C3,Layer1/2,C2,C0,C5,Layer5 |
| 1991 | ***Pnisr*** | C1,Layer1/2,C0 |
| 1992 | ***Pnkd*** | C0 |
| 1993 | ***Pnmal1*** | Layer1/2 |
| 1994 | ***Pnpla8*** | C1,C0 |
| 1995 | ***Pnrc1*** | C0 |
| 1996 | ***Pofut2*** | Layer1/2 |
| 1997 | ***Polb*** | Layer1/2,C0 |
| 1998 | ***Poldip2*** | C1 |
| 1999 | ***Polr2c*** | C1,Layer1/2,C0 |
| 2000 | ***Polr2e*** | C1 |
| 2001 | ***Polr2f*** | Dendritic,Layer6,C1,Layer1/2,C0 |
| 2002 | ***Polr2g*** | C1,Layer1/2,C0,Layer5,Layer3/4 |
| 2003 | ***Polr2i*** | C1,Layer1/2,C0 |
| 2004 | ***Polr2j*** | C1,Layer1/2,C0 |
| 2005 | ***Polr2k*** | Dendritic,Layer6,C1,Layer1/2,C0,Layer5 |
| 2006 | ***Polr3f*** | Layer1/2 |
| 2007 | ***Polr3gl*** | Layer1/2 |
| 2008 | ***Polr3h*** | C0 |
| 2009 | ***Pomp*** | Layer6,C1,Layer1/2,C0 |
| 2010 | ***Pon2*** | C0 |
| 2011 | ***Pop5*** | Dendritic,C1,C3,Layer1/2,C5 |
| 2012 | ***Pop7*** | Layer1/2,C0 |
| 2013 | ***Porcn*** | Layer1/2,C0 |
| 2014 | ***Pou4f1*** | C1 |
| 2015 | ***Pou6f1*** | C0 |
| 2016 | ***Ppa1*** | C1 |
| 2017 | ***Ppa2*** | C1 |
| 2018 | ***Ppfia2*** | Dendritic,Layer1/2 |
| 2019 | ***Ppia*** | Dendritic,Somatic,Layer6,C1,C3,Layer1/2,C4,C0,C5,Layer5,Layer3/4 |
| 2020 | ***Ppib*** | C1,Layer1/2 |
| 2021 | ***Ppig*** | C0 |
| 2022 | ***Ppil4*** | Layer1/2 |
| 2023 | ***Ppip5k1*** | C0 |
| 2024 | ***Ppm1a*** | C1,C2,C0 |
| 2025 | ***Ppm1e*** | Layer6,C1,Layer1/2,C0,C5,Layer5,Layer3/4 |
| 2026 | ***Ppm1h*** | C1 |
| 2027 | ***Ppm1k*** | C0,Layer5 |
| 2028 | ***Ppme1*** | C1 |
| 2029 | ***Ppp1ca*** | Layer1/2 |
| 2030 | ***Ppp1cb*** | C1,C3,C5 |
| 2031 | ***Ppp1cc*** | C1,C2,C0 |
| 2032 | ***Ppp1r11*** | Layer6,C1,Layer1/2,C0,Layer5 |
| 2033 | ***Ppp1r12a*** | Layer1/2 |
| 2034 | ***Ppp1r12c*** | C1,Layer1/2 |
| 2035 | ***Ppp1r13b*** | C1 |
| 2036 | ***Ppp1r14b*** | C1,C0 |
| 2037 | ***Ppp1r1a*** | Layer1/2,C5 |
| 2038 | ***Ppp1r2*** | Dendritic,Layer6,C1,C3,Layer1/2,C2,C5 |
| 2039 | ***Ppp1r37*** | Layer1/2 |
| 2040 | ***Ppp1r9a*** | Layer1/2 |
| 2041 | ***Ppp2ca*** | C1,C3,Layer1/2,C5 |
| 2042 | ***Ppp2r1a*** | Dendritic,Layer6,C1,C3,Layer1/2,C2,C0,Layer5,Layer3/4 |
| 2043 | ***Ppp2r2c*** | Dendritic,C1,Layer1/2 |
| 2044 | ***Ppp2r3c*** | C0 |
| 2045 | ***Ppp2r5a*** | C0 |
| 2046 | ***Ppp2r5b*** | Dendritic,Layer6,C1,C2 |
| 2047 | ***Ppp2r5c*** | C1,C2,C0 |
| 2048 | ***Ppp2r5e*** | C0 |
| 2049 | ***Ppp3ca*** | C1,C3,Layer1/2,C2,C5,Layer3/4 |
| 2050 | ***Ppp3cb*** | Layer6,C1,C3,Layer1/2,C2,C0,C5,Layer5,Layer3/4 |
| 2051 | ***Ppp3r1*** | C3,Layer1/2 |
| 2052 | ***Ppp4r1*** | Layer1/2 |
| 2053 | ***Ppp4r4*** | C2 |
| 2054 | ***Ppp5c*** | C1,C0,Layer3/4 |
| 2055 | ***Ppp6c*** | C1,C0 |
| 2056 | ***Ppp6r3*** | C0 |
| 2057 | ***Ppt1*** | C1 |
| 2058 | ***Praf2*** | Layer6,C1,C0 |
| 2059 | ***Prag1*** | Layer1/2 |
| 2060 | ***Prdx1*** | Layer5 |
| 2061 | ***Prdx2*** | Layer6,C1,C5 |
| 2062 | ***Prdx3*** | C1,C0,Layer3/4 |
| 2063 | ***Prdx5*** | Dendritic,Layer6,C1,Layer1/2,C2,C0,C5,Layer5,Layer3/4 |
| 2064 | ***Prelid1*** | Layer6,C1,Layer1/2,C0,Layer5,Layer3/4 |
| 2065 | ***Prelid3b*** | C1 |
| 2066 | ***Prex1*** | Layer1/2 |
| 2067 | ***Prickle2*** | Layer1/2 |
| 2068 | ***Prkacb*** | Dendritic,Layer6,Layer1/2,Layer5,Layer3/4 |
| 2069 | ***Prkag1*** | Layer1/2,C0 |
| 2070 | ***Prkar1a*** | Dendritic,Layer6,C3,Layer1/2,C0 |
| 2071 | ***Prkar1b*** | C1,Layer1/2 |
| 2072 | ***Prkce*** | C1,C0,C5 |
| 2073 | ***Prmt5*** | C1 |
| 2074 | ***Prpf19*** | C1 |
| 2075 | ***Prpf40b*** | Layer1/2 |
| 2076 | ***Prpf8*** | C1,C2,C0 |
| 2077 | ***Prps1*** | C1,C0 |
| 2078 | ***Prr13*** | C1,C0 |
| 2079 | ***Prrc2a*** | C1,Layer1/2 |
| 2080 | ***Prrc2b*** | Layer1/2,C5,Layer5 |
| 2081 | ***Prrt1*** | C1,Layer1/2,C2,C0 |
| 2082 | ***Prrt3*** | C0 |
| 2083 | ***Prune2*** | C1,C0 |
| 2084 | ***Prxl2a*** | Dendritic,C1,Layer1/2,C0,C5,Layer5 |
| 2085 | ***Prxl2b*** | C1 |
| 2086 | ***Psap*** | Layer1/2 |
| 2087 | ***Psd*** | Layer1/2 |
| 2088 | ***Psd3*** | Dendritic,Layer1/2,C0,Layer5,Layer3/4 |
| 2089 | ***Psenen*** | Dendritic,C1,Layer1/2,C0 |
| 2090 | ***Psma2*** | C1 |
| 2091 | ***Psma3*** | C1,C0,Layer3/4 |
| 2092 | ***Psma5*** | Layer1/2 |
| 2093 | ***Psma6*** | C1,Layer1/2,C0 |
| 2094 | ***Psma7*** | C1,C0 |
| 2095 | ***Psmb1*** | Layer6,C1,C3,Layer1/2,C0,Layer5 |
| 2096 | ***Psmb2*** | C1 |
| 2097 | ***Psmb3*** | C0 |
| 2098 | ***Psmb4*** | C1 |
| 2099 | ***Psmb5*** | Dendritic,C1,Layer1/2,C0,Layer3/4 |
| 2100 | ***Psmb6*** | C1,C4,C0,Layer5 |
| 2101 | ***Psmb7*** | C1,C0 |
| 2102 | ***Psmc1*** | C1 |
| 2103 | ***Psmc3*** | C1 |
| 2104 | ***Psmc4*** | C1 |
| 2105 | ***Psmc5*** | C0 |
| 2106 | ***Psmc6*** | C1,C0 |
| 2107 | ***Psmd12*** | C1 |
| 2108 | ***Psmd13*** | C1 |
| 2109 | ***Psmd14*** | Layer1/2 |
| 2110 | ***Psmd2*** | C1,C2,C0 |
| 2111 | ***Psmd4*** | C1 |
| 2112 | ***Psmd6*** | C1 |
| 2113 | ***Psmd8*** | C1,Layer1/2,C0 |
| 2114 | ***Psme3*** | C1 |
| 2115 | ***Psme4*** | Layer1/2 |
| 2116 | ***Psmg4*** | C0 |
| 2117 | ***Pspc1*** | C0 |
| 2118 | ***Psph*** | Layer1/2 |
| 2119 | ***Pstk*** | C0 |
| 2120 | ***Ptbp2*** | Layer6,Layer1/2 |
| 2121 | ***Ptcd2*** | C1,C0 |
| 2122 | ***Ptges2*** | C1,C0 |
| 2123 | ***Ptk2*** | C3,Layer1/2 |
| 2124 | ***Ptk2b*** | Somatic,Layer6,C1,C3,Layer1/2,C0,C5,Layer5,Layer3/4 |
| 2125 | ***Ptma*** | Layer6,C3,C0 |
| 2126 | ***Ptms*** | C1,C3,Layer1/2,C5 |
| 2127 | ***Ptp4a3*** | Layer1/2 |
| 2128 | ***Ptpmt1*** | C1 |
| 2129 | ***Ptpn11*** | C1 |
| 2130 | ***Ptpn14*** | Layer1/2 |
| 2131 | ***Ptpn3*** | Layer1/2 |
| 2132 | ***Ptpn4*** | C1,C0 |
| 2133 | ***Ptpn5*** | C1,Layer1/2 |
| 2134 | ***Ptpra*** | C1,C0,C5 |
| 2135 | ***Ptprd*** | C1 |
| 2136 | ***Ptprn*** | C1,Layer1/2,C2,C5 |
| 2137 | ***Ptprs*** | C1,Layer1/2,C0,C5,Layer5,Layer3/4 |
| 2138 | ***Ptrh2*** | Layer1/2 |
| 2139 | ***Pum3*** | Layer1/2 |
| 2140 | ***Purb*** | C1 |
| 2141 | ***Pwp1*** | Layer1/2 |
| 2142 | ***Pxdn*** | Layer1/2 |
| 2143 | ***Pygb*** | C0 |
| 2144 | ***Qrich1*** | C1 |
| 2145 | ***Qsox2*** | Layer1/2 |
| 2146 | ***R3hdm2*** | C1,Layer1/2 |
| 2147 | ***Rab10*** | Layer1/2 |
| 2148 | ***Rab11a*** | C1,Layer1/2,C0 |
| 2149 | ***Rab11b*** | Layer6,C1,Layer1/2,C0 |
| 2150 | ***Rab14*** | C1,Layer1/2,C0,Layer5,Layer3/4 |
| 2151 | ***Rab15*** | Layer1/2 |
| 2152 | ***Rab18*** | C1,C0 |
| 2153 | ***Rab1a*** | C1,C2,C0 |
| 2154 | ***Rab24*** | Layer6,C1,C0 |
| 2155 | ***Rab28*** | C0 |
| 2156 | ***Rab2a*** | C1,C3,Layer1/2,C0 |
| 2157 | ***Rab3a*** | Layer6,C1,Layer1/2,C2,C0,Layer3/4 |
| 2158 | ***Rab40b*** | Layer1/2 |
| 2159 | ***Rab4a*** | C1 |
| 2160 | ***Rab4b*** | Layer6,C1,C0 |
| 2161 | ***Rab5a*** | C1,C0 |
| 2162 | ***Rab5if*** | C1 |
| 2163 | ***Rab6a*** | Dendritic,Layer6,C1,Layer1/2,C0,C5,Layer3/4 |
| 2164 | ***Rab6b*** | C1,Layer1/2,C5,Layer3/4 |
| 2165 | ***Rab7*** | C1,Layer1/2 |
| 2166 | ***Rabac1*** | Layer6,C1,Layer1/2,C0,Layer5 |
| 2167 | ***Rabggtb*** | Layer1/2,C0 |
| 2168 | ***Rabif*** | Layer1/2 |
| 2169 | ***Rabl2*** | Layer1/2 |
| 2170 | ***Rabl6*** | Layer1/2,C2,C0 |
| 2171 | ***Rac1*** | Layer6,C1,C3,Layer1/2,C0,C5,Layer5,Layer3/4 |
| 2172 | ***Rack1*** | C1,Layer1/2,C0 |
| 2173 | ***Rad21*** | C0 |
| 2174 | ***Rad23a*** | Dendritic,C1,C0 |
| 2175 | ***Raf1*** | Layer1/2 |
| 2176 | ***Rala*** | C1 |
| 2177 | ***Ralgapb*** | Layer1/2,C0 |
| 2178 | ***Ralyl*** | Layer1/2 |
| 2179 | ***Ramac*** | Layer1/2 |
| 2180 | ***Ramp1*** | Layer6,Layer1/2,C0,Layer5 |
| 2181 | ***Ramp2*** | C1,Layer1/2,C0 |
| 2182 | ***Ran*** | Dendritic,C1,Layer1/2,C0 |
| 2183 | ***Ranbp1*** | C0 |
| 2184 | ***Ranbp9*** | Layer1/2 |
| 2185 | ***Rangap1*** | C1,Layer1/2 |
| 2186 | ***Rap1gap*** | C1,Layer1/2,C2,C0,Layer3/4 |
| 2187 | ***Rap1gds1*** | C1,C2,C0,Layer3/4 |
| 2188 | ***Rapgef2*** | C5 |
| 2189 | ***Rapgef4*** | C1,C0,C5 |
| 2190 | ***Rapgef5*** | C5 |
| 2191 | ***Rapgef6*** | Layer1/2 |
| 2192 | ***Rapgefl1*** | Layer1/2 |
| 2193 | ***Rasa3*** | Layer1/2 |
| 2194 | ***Rasd1*** | Layer1/2 |
| 2195 | ***Rasgef1a*** | Layer1/2 |
| 2196 | ***Rasgrf1*** | Layer1/2,C0,Layer3/4 |
| 2197 | ***Rasgrf2*** | Dendritic,Layer1/2 |
| 2198 | ***Rasgrp1*** | C1,Layer1/2,C2,Layer5,Layer3/4 |
| 2199 | ***Rasl10a*** | Layer1/2 |
| 2200 | ***Rasl10b*** | C5 |
| 2201 | ***Rasl11b*** | Layer1/2 |
| 2202 | ***Rb1*** | Layer1/2 |
| 2203 | ***Rbbp6*** | Layer1/2 |
| 2204 | ***Rbbp7*** | Layer1/2,C2,C5 |
| 2205 | ***Rbfox3*** | C1,C3,Layer1/2 |
| 2206 | ***Rbis*** | Layer6,Layer1/2,Layer5 |
| 2207 | ***Rbm22*** | Layer1/2 |
| 2208 | ***Rbm26*** | Layer1/2 |
| 2209 | ***Rbm28*** | Layer1/2 |
| 2210 | ***Rbm33*** | Layer1/2 |
| 2211 | ***Rbmx*** | Layer1/2 |
| 2212 | ***Rbx1*** | Dendritic,Layer6,C1,C3,Layer1/2,C0,Layer5,Layer3/4 |
| 2213 | ***Rcan1*** | Layer1/2 |
| 2214 | ***Rcc1*** | Layer1/2 |
| 2215 | ***Rcc1l*** | Layer1/2 |
| 2216 | ***Rcc2*** | C1 |
| 2217 | ***Rchy1*** | C0 |
| 2218 | ***Rcn2*** | Dendritic |
| 2219 | ***Rdh13*** | Layer5 |
| 2220 | ***Reep2*** | C1 |
| 2221 | ***Reep5*** | C1,C2,C0 |
| 2222 | ***Rell2*** | Dendritic,C1,Layer1/2,C2,C0 |
| 2223 | ***Reps2*** | C1,C5 |
| 2224 | ***Rer1*** | C0 |
| 2225 | ***Resp18*** | Layer6,C3,Layer1/2,Layer5,Layer3/4 |
| 2226 | ***Retreg2*** | C1,Layer1/2 |
| 2227 | ***Rex1bd*** | C1,Layer1/2,C0 |
| 2228 | ***Rexo1*** | Layer1/2 |
| 2229 | ***Rexo2*** | C1 |
| 2230 | ***Rfx1*** | Layer1/2 |
| 2231 | ***Rfx3*** | Layer1/2 |
| 2232 | ***Rgcc*** | Layer1/2 |
| 2233 | ***Rgs10*** | C5 |
| 2234 | ***Rgs14*** | Layer1/2 |
| 2235 | ***Rgs7*** | Layer1/2 |
| 2236 | ***Rgs7bp*** | C1 |
| 2237 | ***Rgs8*** | C1,C0 |
| 2238 | ***Rheb*** | Dendritic,Layer6,C1,Layer1/2,C0 |
| 2239 | ***Rhoa*** | C0 |
| 2240 | ***Rhob*** | C3,Layer1/2 |
| 2241 | ***Rhot1*** | C1 |
| 2242 | ***Rhpn1*** | Layer1/2 |
| 2243 | ***Rian*** | C3,Layer1/2,C2,Layer5,Layer3/4 |
| 2244 | ***Ric3*** | C1 |
| 2245 | ***Rilpl1*** | Layer1/2,C0 |
| 2246 | ***Rimklb*** | Layer1/2 |
| 2247 | ***Rims1*** | C1,C0 |
| 2248 | ***Rims2*** | C0 |
| 2249 | ***Rims3*** | C2 |
| 2250 | ***Rin1*** | Layer1/2 |
| 2251 | ***Ripor1*** | Layer1/2 |
| 2252 | ***Rit2*** | Dendritic,C1,C0,Layer5 |
| 2253 | ***Rmc1*** | Layer1/2 |
| 2254 | ***Rmnd5b*** | Layer1/2,C0 |
| 2255 | ***Rnasek*** | Layer6,C1,Layer1/2,Layer5 |
| 2256 | ***Rnf10*** | C0 |
| 2257 | ***Rnf11*** | Dendritic,Layer6,C1,Layer1/2,C2,C0,Layer5,Layer3/4 |
| 2258 | ***Rnf112*** | Dendritic,Somatic,Layer1/2 |
| 2259 | ***Rnf115*** | C1 |
| 2260 | ***Rnf123*** | Layer1/2 |
| 2261 | ***Rnf126*** | C1,Layer1/2 |
| 2262 | ***Rnf13*** | C1,C0 |
| 2263 | ***Rnf130*** | C0 |
| 2264 | ***Rnf14*** | Somatic,C1,C3 |
| 2265 | ***Rnf167*** | C1,Layer1/2 |
| 2266 | ***Rnf187*** | C1,Layer1/2,C0,Layer5 |
| 2267 | ***Rnf2*** | Layer1/2 |
| 2268 | ***Rnf215*** | Layer1/2 |
| 2269 | ***Rnf220*** | C1 |
| 2270 | ***Rnf227*** | Dendritic,C0,Layer5 |
| 2271 | ***Rnf4*** | C0 |
| 2272 | ***Rnf41*** | Layer1/2 |
| 2273 | ***Rnf5*** | C1,C0 |
| 2274 | ***Rnf7*** | Layer6,C1,C0,Layer5 |
| 2275 | ***Rnps1*** | Layer1/2,C2,C0 |
| 2276 | ***Rogdi*** | Layer6,C1,Layer1/2,C0,C5,Layer5 |
| 2277 | ***Romo1*** | Dendritic,Layer6,C1,Layer1/2,C4,C0,C5,Layer5,Layer3/4 |
| 2278 | ***Rora*** | C1 |
| 2279 | ***Rorb*** | C2 |
| 2280 | ***Rpa1*** | C0 |
| 2281 | ***Rpp21*** | C1,C0 |
| 2282 | ***Rprml*** | Layer6,Layer1/2,Layer5,Layer3/4 |
| 2283 | ***Rpusd1*** | Layer1/2 |
| 2284 | ***Rraga*** | C1,Layer1/2 |
| 2285 | ***Rragd*** | Layer1/2 |
| 2286 | ***Rreb1*** | Layer1/2 |
| 2287 | ***Rrnad1*** | Layer1/2 |
| 2288 | ***Rrp1*** | C1,Layer1/2,C2 |
| 2289 | ***Rrp7a*** | C1,C0 |
| 2290 | ***Rsl1d1*** | C1 |
| 2291 | ***Rsl24d1*** | C0 |
| 2292 | ***Rsrp1*** | C3,Layer1/2,C0,Layer5,Layer3/4 |
| 2293 | ***Rtca*** | C1,Layer1/2 |
| 2294 | ***Rtcb*** | C1 |
| 2295 | ***Rtf1*** | C0 |
| 2296 | ***Rtf2*** | C1,C0 |
| 2297 | ***Rtl8a*** | C1,C3,Layer1/2,C2,Layer3/4 |
| 2298 | ***Rtl8b*** | C1,C3 |
| 2299 | ***Rtn1*** | Dendritic,Somatic,Layer6,C1,C3,Layer1/2,C2,C4,C0,C5,Layer5,Layer3/4 |
| 2300 | ***Rtn3*** | Layer6,C1,Layer1/2,C4,Layer5,Layer3/4 |
| 2301 | ***Rtn4*** | Layer6,C1,Layer1/2 |
| 2302 | ***Rtn4r*** | Layer1/2 |
| 2303 | ***Rtn4rl1*** | Layer1/2 |
| 2304 | ***Rtraf*** | Layer6,C1,Layer1/2,C0 |
| 2305 | ***Rubcn*** | Layer1/2 |
| 2306 | ***Rufy3*** | Dendritic,C1,C0 |
| 2307 | ***Rundc3a*** | C1,Layer1/2,C2,C0,C5 |
| 2308 | ***Rusc1*** | C1 |
| 2309 | ***Rusc2*** | C1,C0 |
| 2310 | ***Rwdd1*** | C0 |
| 2311 | ***Rxylt1*** | Layer1/2 |
| 2312 | ***Ryr2*** | Dendritic,Layer1/2,C0,Layer3/4 |
| 2313 | ***S100a10*** | C1,Layer1/2,C0 |
| 2314 | ***S1pr1*** | Dendritic,C0 |
| 2315 | ***Sacm1l*** | C1,C2 |
| 2316 | ***Sae1*** | C1 |
| 2317 | ***Samd8*** | Layer1/2 |
| 2318 | ***Samm50*** | C1 |
| 2319 | ***Sap18*** | Layer6,C0 |
| 2320 | ***Sap30*** | Layer1/2 |
| 2321 | ***Sap30l*** | C0 |
| 2322 | ***Sar1b*** | Layer1/2,C0 |
| 2323 | ***Saraf*** | Dendritic,Layer1/2,C0,Layer3/4 |
| 2324 | ***Sarnp*** | C1,Layer1/2,C0 |
| 2325 | ***Sars*** | C1 |
| 2326 | ***Satb1*** | C0,Layer5 |
| 2327 | ***Sbds*** | C0 |
| 2328 | ***Sbno2*** | Layer1/2 |
| 2329 | ***Scamp3*** | Layer1/2 |
| 2330 | ***Scamp5*** | C1,Layer1/2,C0 |
| 2331 | ***Scand1*** | C1 |
| 2332 | ***Scara3*** | Layer1/2 |
| 2333 | ***Scarb2*** | C1 |
| 2334 | ***Scd1*** | C0 |
| 2335 | ***Scg5*** | Dendritic,Layer6,C1,Layer1/2,C0,Layer5,Layer3/4 |
| 2336 | ***Schip1*** | C1,C3,Layer1/2,C4,C5,Layer3/4 |
| 2337 | ***Scn1a*** | C1,C2,C0 |
| 2338 | ***Scn1b*** | C1,Layer1/2,C0,C5 |
| 2339 | ***Scn2a*** | Layer6,Layer1/2,Layer5,Layer3/4 |
| 2340 | ***Scn2b*** | C1 |
| 2341 | ***Scn3b*** | C1,Layer1/2,Layer3/4 |
| 2342 | ***Scnm1*** | Dendritic,Layer5,Layer3/4 |
| 2343 | ***Scoc*** | Dendritic,Layer6,C1,C3,Layer1/2,C0 |
| 2344 | ***Scp2*** | C1 |
| 2345 | ***Scrn3*** | Layer1/2 |
| 2346 | ***Scrt2*** | Layer1/2 |
| 2347 | ***Sdcbp*** | C1,Layer1/2,C0,C5 |
| 2348 | ***Sdf2*** | Dendritic |
| 2349 | ***Sdf4*** | C3 |
| 2350 | ***Sdha*** | Layer6,C1,Layer1/2,C0,Layer5,Layer3/4 |
| 2351 | ***Sdhaf1*** | Layer1/2 |
| 2352 | ***Sdhaf4*** | C1,Layer1/2,C0 |
| 2353 | ***Sdhb*** | Layer6,C1,Layer1/2,C4,C0,C5,Layer5,Layer3/4 |
| 2354 | ***Sdhc*** | C1,C0 |
| 2355 | ***Sdhd*** | C1,Layer1/2 |
| 2356 | ***Sec11c*** | C1 |
| 2357 | ***Sec13*** | C0 |
| 2358 | ***Sec14l1*** | C1,Layer1/2 |
| 2359 | ***Sec14l2*** | Dendritic,Layer1/2 |
| 2360 | ***Sec22b*** | C1 |
| 2361 | ***Sec23a*** | C1 |
| 2362 | ***Sec23b*** | C1,C0 |
| 2363 | ***Sec24b*** | Layer1/2 |
| 2364 | ***Sec61a2*** | Layer1/2 |
| 2365 | ***Sec61b*** | C1,C0 |
| 2366 | ***Sec61g*** | Dendritic,Layer6,C1,C4,C0,Layer5 |
| 2367 | ***Sec62*** | C1 |
| 2368 | ***Secisbp2l*** | C0 |
| 2369 | ***Selenof*** | Dendritic,Somatic,Layer6,C1,C3,Layer1/2,C0,Layer5,Layer3/4 |
| 2370 | ***Selenok*** | Dendritic,Layer6,C1,C3,Layer1/2,C0,Layer5,Layer3/4 |
| 2371 | ***Selenom*** | C1,C3,C4,C0 |
| 2372 | ***Selenop*** | Dendritic,C3 |
| 2373 | ***Selenos*** | C1,Layer1/2,C0 |
| 2374 | ***Selenot*** | C1 |
| 2375 | ***Selenow*** | Layer6,C1,C3,Layer1/2,C0,C5 |
| 2376 | ***Sem1*** | Dendritic,Layer6,C1,C3,Layer1/2,C0,Layer5 |
| 2377 | ***Sema6d*** | C1 |
| 2378 | ***Senp2*** | C0 |
| 2379 | ***Senp3*** | Layer1/2 |
| 2380 | ***Sept3*** | Dendritic,C1,C0 |
| 2381 | ***Sept4*** | C1 |
| 2382 | ***Sept9*** | C5 |
| 2383 | ***Serbp1*** | C1,C3 |
| 2384 | ***Serf2*** | Dendritic,Layer6,C1,Layer1/2,C0,C5,Layer5 |
| 2385 | ***Serinc1*** | Dendritic,Layer6,C1,C3,Layer1/2,C2,C0,C5,Layer5,Layer3/4 |
| 2386 | ***Serinc3*** | C1,Layer1/2 |
| 2387 | ***Serp2*** | Dendritic,Layer6,C1,C3,Layer1/2,C0,C5,Layer5,Layer3/4 |
| 2388 | ***Serpini1*** | C0,Layer5 |
| 2389 | ***Sesn1*** | Layer1/2,C0 |
| 2390 | ***Setd1a*** | Layer1/2 |
| 2391 | ***Setd3*** | C0 |
| 2392 | ***Sez6*** | C1,Layer1/2,C0,Layer5 |
| 2393 | ***Sez6l*** | Dendritic,Layer1/2,C0,Layer5,Layer3/4 |
| 2394 | ***Sez6l2*** | C3,C5 |
| 2395 | ***Sf3b2*** | C1 |
| 2396 | ***Sf3b5*** | Layer6,C1,Layer1/2,C0,C5,Layer5 |
| 2397 | ***Sf3b6*** | Dendritic,Layer6,C1,C0,Layer5 |
| 2398 | ***Sft2d1*** | Layer3/4 |
| 2399 | ***Sfxn3*** | C1 |
| 2400 | ***Sgip1*** | C1,Layer1/2,C0 |
| 2401 | ***Sgk1*** | Dendritic,Layer6,C1,C3,C2,C0,Layer5 |
| 2402 | ***Sgpp2*** | C2 |
| 2403 | ***Sgsm1*** | Layer1/2 |
| 2404 | ***Sh2b1*** | C1,Layer1/2 |
| 2405 | ***Sh2d5*** | Layer1/2 |
| 2406 | ***Sh3bgrl3*** | Layer6,C1,Layer1/2,C0,C5 |
| 2407 | ***Sh3gl1*** | Layer1/2 |
| 2408 | ***Sh3gl2*** | C1,C4,C0 |
| 2409 | ***Sh3glb2*** | C1,Layer1/2 |
| 2410 | ***Shank2*** | Layer1/2,C0 |
| 2411 | ***Shisa4*** | C1,C0 |
| 2412 | ***Sik3*** | C1 |
| 2413 | ***Sirpa*** | Layer1/2,Layer3/4 |
| 2414 | ***Sirt3*** | Layer6,C1,C0 |
| 2415 | ***Skiv2l*** | C0 |
| 2416 | ***Skp1a*** | C1 |
| 2417 | ***Slc12a5*** | C1,C3,Layer1/2,C2,C0,Layer5,Layer3/4 |
| 2418 | ***Slc17a7*** | C1,Layer1/2,C0,Layer3/4 |
| 2419 | ***Slc18b1*** | C5,Layer3/4 |
| 2420 | ***Slc1a1*** | C1 |
| 2421 | ***Slc1a2*** | Dendritic,Layer1/2,C5,Layer3/4 |
| 2422 | ***Slc20a1*** | C1,C0 |
| 2423 | ***Slc20a2*** | C0 |
| 2424 | ***Slc22a17*** | C1,C3,Layer1/2 |
| 2425 | ***Slc24a2*** | C1,Layer1/2,C5 |
| 2426 | ***Slc24a4*** | Layer1/2 |
| 2427 | ***Slc25a11*** | C1,Layer1/2 |
| 2428 | ***Slc25a12*** | C1 |
| 2429 | ***Slc25a19*** | Layer1/2 |
| 2430 | ***Slc25a22*** | Dendritic,C1,Layer1/2,C0,Layer5 |
| 2431 | ***Slc25a3*** | Dendritic,Layer6,C1,Layer1/2,C2,C0,C5,Layer3/4 |
| 2432 | ***Slc25a33*** | C0 |
| 2433 | ***Slc25a39*** | C1 |
| 2434 | ***Slc25a4*** | Dendritic,Layer6,C1,C3,Layer1/2,C2,C0,C5,Layer5,Layer3/4 |
| 2435 | ***Slc25a46*** | C1 |
| 2436 | ***Slc25a5*** | Layer6,C1 |
| 2437 | ***Slc25a51*** | Layer1/2 |
| 2438 | ***Slc27a4*** | C1 |
| 2439 | ***Slc2a1*** | Dendritic,C1,Layer1/2,C0,Layer3/4 |
| 2440 | ***Slc2a3*** | C1,Layer1/2,C2,C0,C5 |
| 2441 | ***Slc30a3*** | C1,Layer1/2 |
| 2442 | ***Slc32a1*** | C1 |
| 2443 | ***Slc35a1*** | Layer1/2 |
| 2444 | ***Slc35a5*** | C1,Layer1/2,C0,Layer3/4 |
| 2445 | ***Slc35c2*** | C1 |
| 2446 | ***Slc35e2*** | Layer1/2 |
| 2447 | ***Slc35f4*** | Layer1/2 |
| 2448 | ***Slc37a4*** | Layer1/2 |
| 2449 | ***Slc38a1*** | C1 |
| 2450 | ***Slc38a2*** | C1,C0 |
| 2451 | ***Slc38a7*** | Layer1/2 |
| 2452 | ***Slc39a3*** | C1,C0 |
| 2453 | ***Slc45a1*** | C1 |
| 2454 | ***Slc48a1*** | C1 |
| 2455 | ***Slc4a10*** | Layer6,C1,C3,Layer1/2,Layer5 |
| 2456 | ***Slc4a3*** | C1 |
| 2457 | ***Slc50a1*** | C0 |
| 2458 | ***Slc6a1*** | Dendritic,C1,C3,Layer1/2 |
| 2459 | ***Slc6a17*** | C1,C5 |
| 2460 | ***Slc6a8*** | C1 |
| 2461 | ***Slc7a14*** | Layer1/2 |
| 2462 | ***Slc7a4*** | Layer1/2,C0 |
| 2463 | ***Slc8a1*** | C1 |
| 2464 | ***Slc9a5*** | Layer1/2 |
| 2465 | ***Slc9a9*** | Layer1/2 |
| 2466 | ***Slirp*** | Dendritic,C1 |
| 2467 | ***Slit1*** | Layer1/2 |
| 2468 | ***Slitrk1*** | C3,Layer1/2,C0 |
| 2469 | ***Smad3*** | Layer1/2 |
| 2470 | ***Smap1*** | C1 |
| 2471 | ***Smap2*** | C1,Layer1/2 |
| 2472 | ***Smarca4*** | C1,Layer1/2,C0 |
| 2473 | ***Smarcd1*** | Layer3/4 |
| 2474 | ***Smarcd3*** | C1 |
| 2475 | ***Smdt1*** | Dendritic,Layer6,C1,C3,Layer1/2,C0,Layer5 |
| 2476 | ***Smg1*** | Layer1/2,Layer5 |
| 2477 | ***Smim10l1*** | Dendritic,Layer6,C1,C3,Layer1/2,C2,C0,Layer5 |
| 2478 | ***Smim12*** | Dendritic,C1,Layer1/2,C0 |
| 2479 | ***Smim13*** | C1,Layer1/2 |
| 2480 | ***Smim14*** | C0 |
| 2481 | ***Smim15*** | C0 |
| 2482 | ***Smim18*** | Layer1/2,C0 |
| 2483 | ***Smim19*** | C1 |
| 2484 | ***Smim20*** | C0 |
| 2485 | ***Smim26*** | Dendritic,Layer6,C1,Layer1/2,C0 |
| 2486 | ***Smim4*** | C0 |
| 2487 | ***Smim7*** | C1,Layer1/2 |
| 2488 | ***Smoc2*** | Layer1/2 |
| 2489 | ***Smpd4*** | Layer1/2 |
| 2490 | ***Sms*** | C0 |
| 2491 | ***Smurf1*** | Layer1/2 |
| 2492 | ***Smyd2*** | C0 |
| 2493 | ***Snap25*** | Dendritic,Layer6,C1,C3,Layer1/2,C2,C0,C5,Layer3/4 |
| 2494 | ***Snap47*** | C1,Layer1/2,C0,Layer3/4 |
| 2495 | ***Snap91*** | Dendritic,C1,Layer1/2 |
| 2496 | ***Snapin*** | C0 |
| 2497 | ***Snca*** | Dendritic,C0 |
| 2498 | ***Sncb*** | C0,C5 |
| 2499 | ***Snf8*** | C1,C0 |
| 2500 | ***Snhg1*** | Layer6,Layer1/2,Layer5 |
| 2501 | ***Snhg11*** | C3,Layer1/2,C2,Layer5,Layer3/4 |
| 2502 | ***Snhg12*** | Dendritic,Layer1/2,C0 |
| 2503 | ***Snhg20*** | Dendritic,Layer1/2,C0 |
| 2504 | ***Snhg6*** | Layer1/2,C0 |
| 2505 | ***Snhg8*** | Layer6,C1,Layer1/2,C0 |
| 2506 | ***Snhg9*** | Layer1/2 |
| 2507 | ***Snn*** | Layer1/2,C0,C5 |
| 2508 | ***Snph*** | C1,C0 |
| 2509 | ***Snrk*** | C0 |
| 2510 | ***Snrnp200*** | C1,Layer1/2 |
| 2511 | ***Snrpb*** | Layer1/2 |
| 2512 | ***Snrpd1*** | Layer6,C1,Layer1/2,C0,Layer5 |
| 2513 | ***Snrpd2*** | C1,C0 |
| 2514 | ***Snrpd3*** | C1,C0 |
| 2515 | ***Snrpe*** | C1,C3,Layer1/2,C4,C0 |
| 2516 | ***Snrpg*** | Layer6,Layer1/2,Layer5 |
| 2517 | ***Snrpn*** | Layer6,C1,Layer1/2,C2,C4,C0,Layer5,Layer3/4 |
| 2518 | ***Sntb2*** | Layer1/2 |
| 2519 | ***Snu13*** | Dendritic,Layer6,C3,Layer1/2,C0 |
| 2520 | ***Snx10*** | C1 |
| 2521 | ***Snx2*** | C1,C0 |
| 2522 | ***Snx24*** | Layer1/2 |
| 2523 | ***Snx3*** | Layer1/2 |
| 2524 | ***Sod2*** | Dendritic,C1,Layer1/2,C2,C0 |
| 2525 | ***Son*** | Layer6,C1,C3,Layer1/2,C2,C0,Layer3/4 |
| 2526 | ***Sorbs2*** | Layer1/2 |
| 2527 | ***Sorcs2*** | C2 |
| 2528 | ***Sorl1*** | Dendritic,Layer6 |
| 2529 | ***Sort1*** | Layer1/2,C5,Layer3/4 |
| 2530 | ***Sowaha*** | C3,Layer1/2 |
| 2531 | ***Spaca6*** | Layer1/2 |
| 2532 | ***Spag5*** | Layer1/2 |
| 2533 | ***Spag9*** | Layer6,C1,Layer1/2,C2,C0,Layer5 |
| 2534 | ***Sparcl1*** | Dendritic,Layer6,C1,Layer1/2,C0,C5,Layer5 |
| 2535 | ***Spats2l*** | Layer1/2 |
| 2536 | ***Spcs1*** | Dendritic,Layer6,C1,Layer1/2,C0,Layer5 |
| 2537 | ***Spcs2*** | C0 |
| 2538 | ***Spg21*** | Layer1/2 |
| 2539 | ***Sphkap*** | C1,Layer1/2,C4,C0,Layer3/4 |
| 2540 | ***Spin1*** | Dendritic,Layer1/2,Layer5 |
| 2541 | ***Spink8*** | Layer3/4 |
| 2542 | ***Spire1*** | Layer1/2,C0 |
| 2543 | ***Spns1*** | C1,C0 |
| 2544 | ***Spns2*** | Layer6,Layer1/2,C0 |
| 2545 | ***Spock1*** | C2,C5 |
| 2546 | ***Spop*** | C1 |
| 2547 | ***Sppl2b*** | Layer1/2 |
| 2548 | ***Spred1*** | Layer1/2 |
| 2549 | ***Spred2*** | Layer1/2 |
| 2550 | ***Sprn*** | C1 |
| 2551 | ***Sptan1*** | C1,Layer1/2,C2,C4,C5 |
| 2552 | ***Sptb*** | Layer1/2,C0 |
| 2553 | ***Sptbn1*** | Dendritic,Somatic,C1,Layer1/2,C2,C0,C5,Layer5,Layer3/4 |
| 2554 | ***Sptbn2*** | C1,C5 |
| 2555 | ***Sptbn4*** | C1,Layer1/2,C2,C0,C5,Layer3/4 |
| 2556 | ***Sqstm1*** | C1,Layer1/2 |
| 2557 | ***Sra1*** | C1,Layer1/2,C0 |
| 2558 | ***Sri*** | C1,C0 |
| 2559 | ***Srm*** | C1 |
| 2560 | ***Srp14*** | Layer6,C1,Layer1/2,C0,C5,Layer5,Layer3/4 |
| 2561 | ***Srp19*** | C1,C3,C0,Layer5 |
| 2562 | ***Srp72*** | C1 |
| 2563 | ***Srp9*** | Layer6,C1,Layer1/2,C0,Layer5 |
| 2564 | ***Srpk2*** | Layer6,C1,C2,C0,Layer5 |
| 2565 | ***Srpr*** | C0 |
| 2566 | ***Srrm1*** | Layer1/2,C0 |
| 2567 | ***Srsf11*** | C1,Layer1/2 |
| 2568 | ***Srsf3*** | C3 |
| 2569 | ***Srsf5*** | Dendritic,Layer6,C1,C3,Layer1/2,C2,C0,Layer5,Layer3/4 |
| 2570 | ***Srxn1*** | C1,Layer1/2,C0 |
| 2571 | ***Ssbp2*** | Layer6,Layer1/2,C0,Layer5 |
| 2572 | ***Ssbp4*** | Layer1/2 |
| 2573 | ***Ssna1*** | C1 |
| 2574 | ***Ssr2*** | C1,Layer1/2,C0 |
| 2575 | ***Ssr4*** | Layer1/2,C0 |
| 2576 | ***Ssrp1*** | C1 |
| 2577 | ***Ssu72*** | C1,Layer1/2,Layer3/4 |
| 2578 | ***St6galnac5*** | C0 |
| 2579 | ***St6galnac6*** | C1 |
| 2580 | ***St7l*** | Layer1/2 |
| 2581 | ***St8sia5*** | Layer1/2 |
| 2582 | ***Stard3*** | Layer1/2 |
| 2583 | ***Stard3nl*** | C0 |
| 2584 | ***Stau2*** | C1,C0 |
| 2585 | ***Stip1*** | C1 |
| 2586 | ***Stk11*** | C0 |
| 2587 | ***Stk16*** | C0 |
| 2588 | ***Stk25*** | C1,Layer1/2 |
| 2589 | ***Stk32c*** | Layer1/2 |
| 2590 | ***Stk39*** | C1 |
| 2591 | ***Stk4*** | Layer1/2 |
| 2592 | ***Stmn1*** | Dendritic,Layer6,C1,C3,Layer1/2,C4,Layer5,Layer3/4 |
| 2593 | ***Stmn2*** | Layer6,C1,C0 |
| 2594 | ***Stmn3*** | Dendritic,Somatic,Layer6,C1,Layer1/2,C2,C4,C0,Layer3/4 |
| 2595 | ***Stmn4*** | C1,Layer1/2 |
| 2596 | ***Stoml1*** | C1,C0 |
| 2597 | ***Stoml2*** | C1 |
| 2598 | ***Strap*** | C1,Layer1/2,C0 |
| 2599 | ***Strbp*** | C1 |
| 2600 | ***Strn4*** | Layer1/2 |
| 2601 | ***Stub1*** | C1 |
| 2602 | ***Stum*** | C5 |
| 2603 | ***Stx12*** | Layer6,C1,C0,Layer5,Layer3/4 |
| 2604 | ***Stx16*** | C0 |
| 2605 | ***Stx1a*** | Layer1/2 |
| 2606 | ***Stx6*** | Layer1/2 |
| 2607 | ***Stx7*** | C1 |
| 2608 | ***Stxbp1*** | C1,Layer1/2,C2 |
| 2609 | ***Stxbp5*** | C0 |
| 2610 | ***Stxbp6*** | C3 |
| 2611 | ***Sub1*** | Dendritic,Layer6,C3,Layer1/2,C0,Layer5,Layer3/4 |
| 2612 | ***Sucla2*** | C1 |
| 2613 | ***Suclg1*** | C1 |
| 2614 | ***Sulf2*** | C1 |
| 2615 | ***Sult4a1*** | C1,Layer1/2,C2,C0,Layer3/4 |
| 2616 | ***Sumo1*** | Layer6,C1,C0,Layer5 |
| 2617 | ***Sumo2*** | Dendritic,Somatic,Layer6,Layer1/2,C4,C0,C5,Layer5 |
| 2618 | ***Sun1*** | Layer1/2 |
| 2619 | ***Supt4a*** | C1 |
| 2620 | ***Supt5*** | C1 |
| 2621 | ***Surf1*** | Dendritic,Layer6,C1,C0 |
| 2622 | ***Suz12*** | Layer1/2 |
| 2623 | ***Sv2a*** | C1 |
| 2624 | ***Sv2b*** | Layer6,C1,Layer1/2,C0,Layer5,Layer3/4 |
| 2625 | ***Svbp*** | Layer5 |
| 2626 | ***Svip*** | C0 |
| 2627 | ***Svop*** | Dendritic,Layer1/2,C0 |
| 2628 | ***Swi5*** | Dendritic,Somatic,Layer6,C1,C3,Layer1/2,C0,Layer5 |
| 2629 | ***Sybu*** | C0 |
| 2630 | ***Syn2*** | Layer6,C1,Layer1/2,C5,Layer3/4 |
| 2631 | ***Syngr3*** | Layer1/2,Layer5 |
| 2632 | ***Synj1*** | Dendritic,C1,C0,Layer5,Layer3/4 |
| 2633 | ***Synj2*** | Layer1/2 |
| 2634 | ***Synpr*** | Dendritic |
| 2635 | ***Syp*** | C1,Layer1/2,C2,C0,Layer3/4 |
| 2636 | ***Sys1*** | Dendritic,C1,C3,Layer1/2,C0 |
| 2637 | ***Syt11*** | C1,C3 |
| 2638 | ***Syt13*** | C1,C0 |
| 2639 | ***Syt16*** | Layer1/2,C0 |
| 2640 | ***Syt17*** | C1,Layer1/2,C0 |
| 2641 | ***Syt3*** | C1 |
| 2642 | ***Syt5*** | Layer6,Layer5 |
| 2643 | ***Syt7*** | C1,C5 |
| 2644 | ***Tac2*** | Layer1/2 |
| 2645 | ***Tacc1*** | Layer1/2 |
| 2646 | ***Tada3*** | Layer1/2 |
| 2647 | ***Taf1*** | C0 |
| 2648 | ***Taf10*** | C1 |
| 2649 | ***Taf11*** | Layer1/2 |
| 2650 | ***Taf1b*** | Layer1/2 |
| 2651 | ***Taf1c*** | Layer1/2 |
| 2652 | ***Taf1d*** | Layer1/2 |
| 2653 | ***Taf5l*** | Layer1/2 |
| 2654 | ***Tafa2*** | Layer1/2,C0 |
| 2655 | ***Tafa5*** | C3 |
| 2656 | ***Tagap1*** | Layer1/2 |
| 2657 | ***Tagln3*** | C1,Layer1/2 |
| 2658 | ***Tango2*** | Dendritic,C1,Layer1/2 |
| 2659 | ***Taok1*** | Dendritic,Layer1/2,C2 |
| 2660 | ***Tardbp*** | Layer1/2 |
| 2661 | ***Tasp1*** | Layer1/2 |
| 2662 | ***Tatdn3*** | Layer6,Layer1/2 |
| 2663 | ***Tax1bp1*** | C1 |
| 2664 | ***Taz*** | Layer1/2,Layer5 |
| 2665 | ***Tbc1d9b*** | Dendritic,C0 |
| 2666 | ***Tbca*** | Dendritic,Somatic,Layer6,C1,C3,Layer1/2,C4,C0,Layer5 |
| 2667 | ***Tbcb*** | C1 |
| 2668 | ***Tbce*** | Layer1/2 |
| 2669 | ***Tbr1*** | C1,C0 |
| 2670 | ***Tcaf1*** | Dendritic,Layer1/2,C0 |
| 2671 | ***Tceal1*** | Layer1/2,C0 |
| 2672 | ***Tceal3*** | Layer1/2,C0,Layer5 |
| 2673 | ***Tceal5*** | C1,C5 |
| 2674 | ***Tceal6*** | Layer1/2 |
| 2675 | ***Tceal8*** | Layer6,C1 |
| 2676 | ***Tceal9*** | C0 |
| 2677 | ***Tcerg1l*** | Layer1/2 |
| 2678 | ***Tcf25*** | C1 |
| 2679 | ***Tcf4*** | C5 |
| 2680 | ***Tcf7l2*** | C2 |
| 2681 | ***Tcp1*** | C1,C3,Layer1/2,C0 |
| 2682 | ***Tcta*** | Layer1/2,C0 |
| 2683 | ***Tctex1d2*** | Layer6,Layer1/2 |
| 2684 | ***Tdg*** | Layer1/2 |
| 2685 | ***Tecpr1*** | C1 |
| 2686 | ***Ten1*** | C1 |
| 2687 | ***Tepsin*** | Layer1/2 |
| 2688 | ***Tesc*** | Layer1/2 |
| 2689 | ***Tex264*** | C1 |
| 2690 | ***Tfb2m*** | Layer1/2 |
| 2691 | ***Tfg*** | C1 |
| 2692 | ***Tfr2*** | Layer1/2 |
| 2693 | ***Tfrc*** | C1 |
| 2694 | ***Tgm2*** | Layer1/2 |
| 2695 | ***Thap7*** | Layer1/2 |
| 2696 | ***Thoc7*** | C1 |
| 2697 | ***Thra*** | Dendritic,C1,C3,Layer1/2,C0,C5 |
| 2698 | ***Thrb*** | Layer1/2 |
| 2699 | ***Thumpd1*** | C0 |
| 2700 | ***Thy1*** | C2 |
| 2701 | ***Thyn1*** | C1,Layer1/2,C0 |
| 2702 | ***Tia1*** | Layer1/2,C0 |
| 2703 | ***Tiam1*** | Layer1/2,C0 |
| 2704 | ***Tiam2*** | Layer1/2 |
| 2705 | ***Timm10*** | Layer6,C1,C3,Layer1/2,C0,C5,Layer5 |
| 2706 | ***Timm10b*** | C1,Layer1/2 |
| 2707 | ***Timm13*** | Layer6,C1,C3,Layer1/2,C0,C5,Layer5 |
| 2708 | ***Timm17a*** | C1,C0 |
| 2709 | ***Timm23*** | Layer6,C1,C0,Layer5 |
| 2710 | ***Timm8b*** | Dendritic,Somatic,Layer6,C1,C3,Layer1/2,C0,Layer5,Layer3/4 |
| 2711 | ***Tle5*** | Layer6,C1,Layer1/2,C2,C4,C0 |
| 2712 | ***Tln1*** | C1 |
| 2713 | ***Tm2d1*** | Layer6,C0 |
| 2714 | ***Tm2d2*** | Layer6,C1,C0,C5 |
| 2715 | ***Tm2d3*** | C1 |
| 2716 | ***Tm9sf3*** | C3 |
| 2717 | ***Tma7*** | Dendritic,Somatic,Layer6,C1,C3,Layer1/2,C0,Layer5,Layer3/4 |
| 2718 | ***Tmbim4*** | C0 |
| 2719 | ***Tmco1*** | C1,C0 |
| 2720 | ***Tmed7*** | Dendritic |
| 2721 | ***Tmed9*** | Layer1/2,C0 |
| 2722 | ***Tmeff1*** | C0 |
| 2723 | ***Tmeff2*** | Layer1/2 |
| 2724 | ***Tmem109*** | Layer1/2 |
| 2725 | ***Tmem11*** | C0 |
| 2726 | ***Tmem121*** | Layer1/2 |
| 2727 | ***Tmem126a*** | Somatic,Layer1/2,C0 |
| 2728 | ***Tmem128*** | C1 |
| 2729 | ***Tmem135*** | C1 |
| 2730 | ***Tmem147*** | C1 |
| 2731 | ***Tmem14a*** | C1,Layer1/2,C0 |
| 2732 | ***Tmem14c*** | C0 |
| 2733 | ***Tmem151a*** | C3,Layer1/2 |
| 2734 | ***Tmem160*** | C1 |
| 2735 | ***Tmem167*** | C1 |
| 2736 | ***Tmem178*** | C0,C5 |
| 2737 | ***Tmem183a*** | C2 |
| 2738 | ***Tmem184c*** | C0 |
| 2739 | ***Tmem191c*** | Layer1/2 |
| 2740 | ***Tmem198*** | Layer1/2 |
| 2741 | ***Tmem205*** | C0 |
| 2742 | ***Tmem208*** | C1 |
| 2743 | ***Tmem209*** | Layer1/2,Layer3/4 |
| 2744 | ***Tmem222*** | C1,Layer1/2 |
| 2745 | ***Tmem223*** | Dendritic,C1,Layer1/2,C0 |
| 2746 | ***Tmem234*** | C1 |
| 2747 | ***Tmem240*** | Layer1/2 |
| 2748 | ***Tmem242*** | C1 |
| 2749 | ***Tmem243*** | C0 |
| 2750 | ***Tmem256*** | Dendritic,Layer6,C1,Layer1/2,C0,C5,Layer5 |
| 2751 | ***Tmem258*** | Layer6,C1 |
| 2752 | ***Tmem259*** | Layer6,C1 |
| 2753 | ***Tmem30a*** | Dendritic,C1,C3,Layer1/2,Layer3/4 |
| 2754 | ***Tmem33*** | C1 |
| 2755 | ***Tmem38a*** | C1 |
| 2756 | ***Tmem50a*** | Layer6,C1,Layer1/2,C0,Layer5 |
| 2757 | ***Tmem59*** | Dendritic,Layer6,C1,C3,Layer1/2,C0 |
| 2758 | ***Tmem59l*** | C1,Layer1/2,C0 |
| 2759 | ***Tmem63b*** | C1 |
| 2760 | ***Tmem70*** | C1 |
| 2761 | ***Tmem9b*** | Dendritic,C0,Layer5 |
| 2762 | ***Tmod2*** | C1,C0 |
| 2763 | ***Tmsb10*** | C1,C0 |
| 2764 | ***Tmsb4x*** | Dendritic,Layer6,C3,Layer1/2,C4,C0,C5,Layer5,Layer3/4 |
| 2765 | ***Tmub2*** | C1 |
| 2766 | ***Tmx2*** | C1 |
| 2767 | ***Tnfrsf21*** | C1,C3 |
| 2768 | ***Tnks*** | C0 |
| 2769 | ***Tnnt1*** | C1 |
| 2770 | ***Tnrc6c*** | C5 |
| 2771 | ***Tns3*** | C0 |
| 2772 | ***Toe1*** | Layer1/2 |
| 2773 | ***Tollip*** | C1,C0 |
| 2774 | ***Tomm20*** | C1,C3,Layer1/2,C2 |
| 2775 | ***Tomm22*** | C1,Layer1/2,C0 |
| 2776 | ***Tomm34*** | C1,Layer1/2 |
| 2777 | ***Tomm40*** | C1 |
| 2778 | ***Tomm40l*** | C1 |
| 2779 | ***Tomm5*** | Dendritic,Layer6,C1,Layer1/2,C0 |
| 2780 | ***Tomm6*** | C1,Layer1/2,C0 |
| 2781 | ***Tomm7*** | Dendritic,Layer6,C1,C3,Layer1/2,C0,Layer5 |
| 2782 | ***Tomm70a*** | C1 |
| 2783 | ***Tox*** | C2 |
| 2784 | ***Tox4*** | Layer1/2 |
| 2785 | ***Tpgs1*** | C1 |
| 2786 | ***Tpgs2*** | C1,C0 |
| 2787 | ***Tpi1*** | C1,C3,Layer1/2,C5,Layer5,Layer3/4 |
| 2788 | ***Tpm1*** | C1,C3,Layer1/2 |
| 2789 | ***Tprgl*** | Dendritic,Layer6,C1,C3,Layer1/2,C0,Layer5 |
| 2790 | ***Tpst2*** | Layer1/2 |
| 2791 | ***Tpt1*** | Dendritic,Somatic,Layer6,C1,C3,Layer1/2,C4,C0,C5,Layer5,Layer3/4 |
| 2792 | ***Traf7*** | Layer1/2 |
| 2793 | ***Trafd1*** | Layer1/2,C0 |
| 2794 | ***Trank1*** | Layer1/2,C0 |
| 2795 | ***Trap1*** | C1 |
| 2796 | ***Trappc1*** | C1 |
| 2797 | ***Trappc12*** | C0 |
| 2798 | ***Trappc2*** | C1,C0,Layer5 |
| 2799 | ***Trappc6b*** | Dendritic,C1,Layer1/2 |
| 2800 | ***Trim2*** | C1,C3,C0 |
| 2801 | ***Trim3*** | C1,Layer1/2,Layer3/4 |
| 2802 | ***Trim32*** | Dendritic,Layer6,C1,Layer1/2 |
| 2803 | ***Trim44*** | C2 |
| 2804 | ***Trim9*** | Layer1/2,C5 |
| 2805 | ***Trio*** | Layer6,C1,Layer1/2 |
| 2806 | ***Trir*** | C1 |
| 2807 | ***Trmt1*** | Layer1/2 |
| 2808 | ***Trmt112*** | Layer6,C1,C0 |
| 2809 | ***Trmt2a*** | Layer1/2 |
| 2810 | ***Trnp1*** | Dendritic,C1,C0,C5,Layer3/4 |
| 2811 | ***Trp53i11*** | C3,Layer1/2 |
| 2812 | ***Trpc1*** | Layer1/2 |
| 2813 | ***Trpc4ap*** | C1,C0 |
| 2814 | ***Trrap*** | Dendritic |
| 2815 | ***Tsc22d1*** | Dendritic,Layer6,C1,C3,Layer1/2,C0,C5,Layer5,Layer3/4 |
| 2816 | ***Tsc22d3*** | C2 |
| 2817 | ***Tsen15*** | C1,Layer1/2,C0 |
| 2818 | ***Tsn*** | C1,C0 |
| 2819 | ***Tsnax*** | Dendritic,Layer1/2 |
| 2820 | ***Tspan13*** | Layer6,C1,C3,C0,C5,Layer5,Layer3/4 |
| 2821 | ***Tspan3*** | Dendritic,C1 |
| 2822 | ***Tspan5*** | C1,Layer1/2,C2,C0 |
| 2823 | ***Tspan7*** | Dendritic,Layer6,C1,C3,Layer1/2,C2,C0,Layer5,Layer3/4 |
| 2824 | ***Tspyl1*** | C1,Layer1/2 |
| 2825 | ***Tspyl4*** | Dendritic,Layer6,C1,Layer1/2,C2,C0,Layer5,Layer3/4 |
| 2826 | ***Ttc14*** | C0 |
| 2827 | ***Ttc19*** | C1 |
| 2828 | ***Ttc3*** | Dendritic,Somatic,C1,Layer1/2,Layer5 |
| 2829 | ***Ttc4*** | C0,Layer5 |
| 2830 | ***Ttc7b*** | C5 |
| 2831 | ***Ttc9*** | C1 |
| 2832 | ***Ttc9b*** | C3,Layer1/2,C0 |
| 2833 | ***Ttll7*** | C1 |
| 2834 | ***Ttpal*** | Layer1/2 |
| 2835 | ***Ttyh1*** | C1,Layer1/2,C2,C0,Layer5 |
| 2836 | ***Tub*** | Layer1/2 |
| 2837 | ***Tuba1b*** | C1,C4,Layer3/4 |
| 2838 | ***Tuba4a*** | C1 |
| 2839 | ***Tubb3*** | C1,Layer1/2,C2,C0,Layer3/4 |
| 2840 | ***Tusc1*** | Layer1/2 |
| 2841 | ***Tusc2*** | C1,Layer1/2 |
| 2842 | ***Tusc3*** | Layer1/2,Layer5 |
| 2843 | ***Twf2*** | C1,Layer1/2 |
| 2844 | ***Txn1*** | Dendritic,Layer6,C1,C3,Layer1/2,C2,Layer5 |
| 2845 | ***Txn2*** | C1,C0 |
| 2846 | ***Txndc16*** | C0 |
| 2847 | ***Txndc9*** | C1,C0 |
| 2848 | ***Txnip*** | C0 |
| 2849 | ***Tyro3*** | Layer1/2 |
| 2850 | ***U2af1l4*** | Layer1/2 |
| 2851 | ***U2surp*** | C0 |
| 2852 | ***Uap1*** | C0 |
| 2853 | ***Uba1*** | C1,C0 |
| 2854 | ***Uba3*** | Dendritic,C1,Layer1/2 |
| 2855 | ***Uba5*** | Layer1/2,C0 |
| 2856 | ***Uba52*** | Dendritic,Somatic,Layer6,C1,C3,Layer1/2,C2,C4,C0,C5,Layer5,Layer3/4 |
| 2857 | ***Ubald1*** | Layer1/2 |
| 2858 | ***Ubb*** | Dendritic,Somatic,Layer6,C1,C3,Layer1/2,C2,C0,C5,Layer5,Layer3/4 |
| 2859 | ***Ubc*** | C1,C0 |
| 2860 | ***Ube2a*** | C1 |
| 2861 | ***Ube2b*** | Somatic,Layer6,Layer1/2,C0 |
| 2862 | ***Ube2d2a*** | Dendritic |
| 2863 | ***Ube2d3*** | C1,Layer1/2 |
| 2864 | ***Ube2e1*** | C1,C3,C0 |
| 2865 | ***Ube2e3*** | C1,C3,Layer1/2,C5 |
| 2866 | ***Ube2g2*** | Layer1/2 |
| 2867 | ***Ube2i*** | C0 |
| 2868 | ***Ube2j2*** | Layer1/2 |
| 2869 | ***Ube2k*** | Dendritic,C1 |
| 2870 | ***Ube2l3*** | C1 |
| 2871 | ***Ube2n*** | Dendritic,C1,Layer1/2,C0,Layer3/4 |
| 2872 | ***Ube2o*** | Layer1/2 |
| 2873 | ***Ube2q2*** | C1,Layer1/2,C0 |
| 2874 | ***Ube2ql1*** | Layer6,C1,C3,Layer1/2,C2,C0,Layer3/4 |
| 2875 | ***Ube2r2*** | C1 |
| 2876 | ***Ube2v1*** | C1 |
| 2877 | ***Ube2v2*** | C1,Layer1/2,Layer3/4 |
| 2878 | ***Ube2w*** | C1,Layer1/2 |
| 2879 | ***Ube3b*** | C1,Layer1/2,C0 |
| 2880 | ***Ube3c*** | Dendritic,C1,C0 |
| 2881 | ***Ubl4a*** | C1 |
| 2882 | ***Ubl5*** | Dendritic,Layer6,C1,C3,Layer1/2,C0,Layer5 |
| 2883 | ***Ubl7*** | C1,C0 |
| 2884 | ***Ublcp1*** | C1,C0 |
| 2885 | ***Ubn2*** | Layer1/2 |
| 2886 | ***Ubox5*** | Layer1/2 |
| 2887 | ***Ubqln2*** | C1,Layer1/2,C0 |
| 2888 | ***Ubqln4*** | C0 |
| 2889 | ***Ubr3*** | C1,Layer1/2 |
| 2890 | ***Ubtf*** | C1,C0 |
| 2891 | ***Ubxn1*** | C0 |
| 2892 | ***Ubxn6*** | C1 |
| 2893 | ***Uchl1*** | Dendritic,Layer6,C1,Layer1/2,C2,C0,Layer5,Layer3/4 |
| 2894 | ***Uchl5*** | C1,C0 |
| 2895 | ***Uckl1*** | Layer1/2 |
| 2896 | ***Ufc1*** | C1,Layer1/2,C0 |
| 2897 | ***Ufm1*** | C1 |
| 2898 | ***Ufsp2*** | C1 |
| 2899 | ***Ugcg*** | C1 |
| 2900 | ***Uhrf1bp1l*** | C1,C2 |
| 2901 | ***Ulk1*** | C1 |
| 2902 | ***Ulk2*** | C3,C0 |
| 2903 | ***Unc13a*** | C1,Layer1/2 |
| 2904 | ***Unc13b*** | Layer1/2 |
| 2905 | ***Unc50*** | Layer1/2,C0 |
| 2906 | ***Unc5a*** | C1,Layer1/2,C0 |
| 2907 | ***Unc5d*** | Layer1/2 |
| 2908 | ***Unc80*** | Dendritic,Layer6,C1,Layer1/2,C2,C0 |
| 2909 | ***Uqcc1*** | C0 |
| 2910 | ***Uqcc2*** | Dendritic,Layer6,C1,Layer1/2,C4,C0,C5 |
| 2911 | ***Uqcc3*** | C1,C0 |
| 2912 | ***Uqcr10*** | Dendritic,Somatic,Layer6,C1,C3,Layer1/2,C0,C5,Layer5,Layer3/4 |
| 2913 | ***Uqcr11*** | Dendritic,Somatic,Layer6,C1,C3,Layer1/2,C0,C5,Layer5,Layer3/4 |
| 2914 | ***Uqcrb*** | Dendritic,Somatic,Layer6,C1,C3,Layer1/2,C2,C0,C5,Layer5,Layer3/4 |
| 2915 | ***Uqcrc1*** | C1,C0 |
| 2916 | ***Uqcrc2*** | Layer6,C1,Layer1/2,C0,Layer5 |
| 2917 | ***Uqcrfs1*** | Layer6,C1,C3,Layer1/2,C0,C5 |
| 2918 | ***Uqcrh*** | Dendritic,Somatic,Layer6,C1,C3,Layer1/2,C2,C4,C0,C5,Layer5,Layer3/4 |
| 2919 | ***Uqcrq*** | Dendritic,Somatic,Layer6,C1,C3,Layer1/2,C0,C5,Layer5,Layer3/4 |
| 2920 | ***Urgcp*** | C1 |
| 2921 | ***Urm1*** | C0 |
| 2922 | ***Use1*** | C1 |
| 2923 | ***Uso1*** | C0 |
| 2924 | ***Usp12*** | Layer1/2 |
| 2925 | ***Usp19*** | Layer1/2 |
| 2926 | ***Usp2*** | C1,C0 |
| 2927 | ***Usp22*** | Dendritic,Layer1/2,C2,C0,Layer5 |
| 2928 | ***Usp30*** | Layer1/2 |
| 2929 | ***Usp31*** | C0 |
| 2930 | ***Usp33*** | Dendritic,C1 |
| 2931 | ***Usp39*** | Layer1/2 |
| 2932 | ***Usp5*** | C1 |
| 2933 | ***Usp50*** | Layer1/2 |
| 2934 | ***Usp54*** | C0 |
| 2935 | ***Usp9x*** | Layer6,Layer1/2,C2 |
| 2936 | ***Uvrag*** | Layer1/2 |
| 2937 | ***Vamp2*** | Dendritic,Somatic,Layer6,C1,C3,Layer1/2,C2,C0,C5,Layer5,Layer3/4 |
| 2938 | ***Vamp4*** | Layer1/2 |
| 2939 | ***Vapa*** | C1,Layer1/2,Layer5,Layer3/4 |
| 2940 | ***Vapb*** | C0 |
| 2941 | ***Vdac1*** | Layer6,C1,Layer1/2,C2,C0,C5,Layer3/4 |
| 2942 | ***Vdac2*** | Dendritic,Layer6,C1,C3,Layer1/2,C2,C4,C0 |
| 2943 | ***Vdac3*** | Dendritic,C1,C2 |
| 2944 | ***Vegfb*** | C0 |
| 2945 | ***Vip*** | Layer1/2,Layer3/4 |
| 2946 | ***Vipr1*** | Layer1/2 |
| 2947 | ***Vma21*** | C1 |
| 2948 | ***Vmp1*** | C1 |
| 2949 | ***Vps25*** | Layer1/2 |
| 2950 | ***Vps26b*** | Layer6 |
| 2951 | ***Vps28*** | Layer6,C1,Layer1/2,C0,Layer5 |
| 2952 | ***Vps29*** | Layer6,C1,Layer1/2,C0 |
| 2953 | ***Vps37b*** | Layer1/2 |
| 2954 | ***Vps39*** | C0 |
| 2955 | ***Vps41*** | C1,C2 |
| 2956 | ***Vps45*** | C1 |
| 2957 | ***Vps4a*** | C1,C0 |
| 2958 | ***Vps4b*** | Layer1/2 |
| 2959 | ***Vps50*** | C0 |
| 2960 | ***Vps52*** | C1 |
| 2961 | ***Vps72*** | Layer1/2 |
| 2962 | ***Vps8*** | C0 |
| 2963 | ***Vps9d1*** | Layer1/2 |
| 2964 | ***Vsnl1*** | Dendritic,Layer6,Layer1/2,C2,C0,Layer5,Layer3/4 |
| 2965 | ***Vstm2a*** | C1 |
| 2966 | ***Vstm2b*** | Layer1/2 |
| 2967 | ***Vti1b*** | Layer6,C1,C0 |
| 2968 | ***Vxn*** | Layer6,C1 |
| 2969 | ***Wasf1*** | C1,C3,Layer1/2,C5 |
| 2970 | ***Wasl*** | C1 |
| 2971 | ***Wbp11*** | Dendritic,Somatic,Layer6,C3,Layer1/2,C0,C5,Layer5,Layer3/4 |
| 2972 | ***Wbp2*** | Layer6,C1,Layer1/2,Layer5,Layer3/4 |
| 2973 | ***Wdr13*** | Layer1/2 |
| 2974 | ***Wdr4*** | Layer1/2 |
| 2975 | ***Wdr45*** | Layer1/2,C0 |
| 2976 | ***Wdr45b*** | Layer1/2,C0 |
| 2977 | ***Wdr47*** | C1,Layer1/2 |
| 2978 | ***Wdr61*** | Layer1/2 |
| 2979 | ***Wdr7*** | C0 |
| 2980 | ***Wdr82*** | Layer1/2 |
| 2981 | ***Wdr83os*** | C1,C0 |
| 2982 | ***Wdr89*** | Dendritic,C1,C3,Layer1/2,C0 |
| 2983 | ***Wipf3*** | C1,C3,Layer1/2 |
| 2984 | ***Wnk2*** | C1,Layer1/2 |
| 2985 | ***Wnt10a*** | Layer1/2 |
| 2986 | ***Wrb*** | C0 |
| 2987 | ***Wsb1*** | Layer1/2,C2,C0 |
| 2988 | ***Wsb2*** | C1,Layer1/2,C0 |
| 2989 | ***Wscd2*** | Layer1/2 |
| 2990 | ***Xpo7*** | Layer1/2 |
| 2991 | ***Xylt2*** | Layer1/2 |
| 2992 | ***Yaf2*** | Dendritic,C1,Layer1/2,C0 |
| 2993 | ***Yars*** | C1 |
| 2994 | ***Ydjc*** | Layer1/2 |
| 2995 | ***Yipf4*** | C1 |
| 2996 | ***Ypel3*** | Dendritic,Layer6,C1,C3,Layer1/2,C2,C0,Layer5,Layer3/4 |
| 2997 | ***Ypel4*** | Layer1/2,C0 |
| 2998 | ***Ypel5*** | Layer3/4 |
| 2999 | ***Yrdc*** | Layer1/2 |
| 3000 | ***Ythdc2*** | Layer1/2 |
| 3001 | ***Ywhae*** | Dendritic,Somatic,Layer6,C1,C3,Layer1/2,C2,C0,C5,Layer5,Layer3/4 |
| 3002 | ***Ywhag*** | C1,C2,Layer3/4 |
| 3003 | ***Ywhah*** | Dendritic,Layer6,C1,C3,Layer1/2,C4,Layer5,Layer3/4 |
| 3004 | ***Ywhaq*** | Dendritic,Layer6,C1,Layer1/2,C4,C0,Layer5,Layer3/4 |
| 3005 | ***Ywhaz*** | Somatic,Layer6,C1,C3,Layer1/2,C2,C0,C5,Layer5,Layer3/4 |
| 3006 | ***Zbtb18*** | C1,Layer1/2,C0,Layer5,Layer3/4 |
| 3007 | ***Zbtb33*** | Layer1/2 |
| 3008 | ***Zc3h15*** | C1 |
| 3009 | ***Zcchc14*** | Layer1/2 |
| 3010 | ***Zcchc17*** | C0 |
| 3011 | ***Zcchc18*** | Dendritic,Layer6,Layer1/2,C0,Layer5 |
| 3012 | ***Zcrb1*** | C1,C0 |
| 3013 | ***Zdhhc16*** | Layer1/2 |
| 3014 | ***Zdhhc17*** | C1,Layer1/2,C0 |
| 3015 | ***Zdhhc22*** | Layer1/2 |
| 3016 | ***Zdhhc8*** | Layer1/2 |
| 3017 | ***Zfand2b*** | Layer1/2 |
| 3018 | ***Zfand5*** | C1,C3,Layer1/2 |
| 3019 | ***Zfand6*** | C1 |
| 3020 | ***Zfas1*** | Dendritic,Layer6,C1,C3,Layer1/2,C0,Layer5 |
| 3021 | ***Zfc3h1*** | Layer1/2 |
| 3022 | ***Zfp1*** | Layer1/2 |
| 3023 | ***Zfp119a*** | Layer1/2 |
| 3024 | ***Zfp131*** | Layer1/2 |
| 3025 | ***Zfp14*** | Layer1/2 |
| 3026 | ***Zfp180*** | Layer1/2 |
| 3027 | ***Zfp276*** | Layer1/2 |
| 3028 | ***Zfp346*** | Layer1/2 |
| 3029 | ***Zfp365*** | C1,Layer1/2,C5 |
| 3030 | ***Zfp369*** | Layer1/2 |
| 3031 | ***Zfp384*** | Layer1/2 |
| 3032 | ***Zfp445*** | C0 |
| 3033 | ***Zfp46*** | Layer1/2 |
| 3034 | ***Zfp511*** | Layer1/2 |
| 3035 | ***Zfp513*** | Layer1/2 |
| 3036 | ***Zfp523*** | C0 |
| 3037 | ***Zfp560*** | Layer1/2 |
| 3038 | ***Zfp580*** | Layer1/2 |
| 3039 | ***Zfp606*** | Layer1/2 |
| 3040 | ***Zfp664*** | C0 |
| 3041 | ***Zfp692*** | Layer1/2 |
| 3042 | ***Zfp697*** | Layer1/2 |
| 3043 | ***Zfp706*** | Layer6,C1,Layer1/2,C5,Layer3/4 |
| 3044 | ***Zfp738*** | Layer1/2 |
| 3045 | ***Zfp74*** | Layer1/2 |
| 3046 | ***Zfp821*** | Layer1/2 |
| 3047 | ***Zfp871*** | Layer1/2 |
| 3048 | ***Zfp938*** | Layer1/2 |
| 3049 | ***Zfr*** | Layer6,C3,Layer1/2,C2,C0 |
| 3050 | ***Zmat2*** | Layer1/2,Layer3/4 |
| 3051 | ***Zmiz1*** | C1,Layer1/2 |
| 3052 | ***Zmynd19*** | Layer1/2 |
| 3053 | ***Zmynd8*** | C3,Layer1/2 |
| 3054 | ***Znhit1*** | C1 |
| 3055 | ***Znhit2*** | C1 |
| 3056 | ***Znhit3*** | Layer6,C1,Layer1/2,C0 |
| 3057 | ***Znrd2*** | C0 |
| 3058 | ***Zranb2*** | C1,Layer1/2,C2,C0 |
| 3059 | ***Zrsr2*** | C1 |
| 3060 | ***Zscan26*** | C0 |
| 3061 | ***Zswim6*** | Layer1/2 |
| 3062 | ***Zswim8*** | Layer1/2 |
| 3063 | ***Zwint*** | Dendritic,Somatic,Layer6,C3,Layer1/2,C2,C0,Layer5,Layer3/4 |
| 3064 | ***Zxdb*** | Layer1/2 |
| 3065 | ***Zyx*** | Layer1/2 |
