# Supplementary material for: Modifying Glucose Metabolism Reverses Memory Defects of Alzheimer's Disease Model at Late Stages
Source: Adv Sci (Weinh). 2025 Dec 8;13(11):e06695. doi: 10.1002/advs.202506695 (PMC12931168; doi:10.1002/advs.202506695)
Supplement: Supplementary file 3 — Supporting Information [file ADVS-13-e06695-s003.docx]

**Table S5. Top30 AAR genes across all brain regions (101 genes in total).**

| ID | **AAR genes** | **Region** | **Pvalue** |
| --- | --- | --- | --- |
| 1 | ***Sgk1*** | C0,C1,C2,Dendritic | C0:0.00e+00 ; C1:1.80e-287 ; C2:9.02e-105 ; Dendritic:1.16e-98 |
| 2 | ***Cox8a*** | C0,C1,C2,C3,C4,C5,Dendritic,Layer1/2,  Layer3/4,Layer5,Layer6,Somatic | C0:1.12e-200 ; C1:2.83e-309 ; C2:7.79e-210 ; C3:8.25e-151 ; C4:4.24e-39 ; C5:2.06e-120 ; Dendritic:4.05e-236 ; Layer1/2:4.07e-304 ; Layer3/4:3.47e-165 ; Layer5:3.47e-157 ; Layer6:4.90e-163 ; Somatic:6.94e-90 |
| 3 | ***Ppia*** | C0,C1,C3,C4,C5,Dendritic,Layer1/2,  Layer3/4,Layer5,Layer6,Somatic | C0:2.22e-165 ; C1:2.47e-147 ; C3:5.18e-92 ; C4:2.25e-27 ; C5:1.59e-84 ; Dendritic:3.15e-150 ; Layer1/2:5.85e-209 ; Layer3/4:8.27e-111 ; Layer5:2.22e-113 ; Layer6:3.31e-124 ; Somatic:2.50e-67 |
| 4 | ***Arf5*** | C0,C1,C2,C3,C5,Layer1/2,Layer5,Layer6 | C0:1.26e-160 ; C1:5.57e-247 ; C2:6.91e-116 ; C3:1.41e-93 ; C5:2.06e-55 ; Layer1/2:4.92e-192 ; Layer5:3.14e-96 ; Layer6:4.01e-114 |
| 5 | ***Ndrg2*** | C0,C2 | C0:1.48e-138 ; C2:2.15e-102 |
| 6 | ***Rsrp1*** | C0 | C0:2.33e-134 |
| 7 | ***Cox6a1*** | C0,C1,C2,C3,C5,Dendritic,Layer1/2,  Layer3/4,Layer5,Layer6,Somatic | C0:1.73e-131 ; C1:4.74e-168 ; C2:1.43e-102 ; C3:3.54e-93 ; C5:6.78e-82 ; Dendritic:8.67e-166 ; Layer1/2:8.45e-194 ; Layer3/4:2.54e-104 ; Layer5:9.29e-94 ; Layer6:4.30e-113 ; Somatic:2.08e-70 |
| 8 | ***Pink1*** | C0,C1 | C0:2.77e-130 ; C1:4.61e-214 |
| 9 | ***Tpt1*** | C0,C1,C4,Dendritic | C0:4.33e-128 ; C1:3.64e-146 ; C4:3.52e-23 ; Dendritic:6.13e-105 |
| 10 | ***Cox4i1*** | C0,C1,C3,C4,C5,Dendritic,Layer1/2,  Layer3/4,Layer5,Layer6,Somatic | C0:1.36e-127 ; C1:2.17e-163 ; C3:4.63e-116 ; C4:8.21e-24 ; C5:7.19e-76 ; Dendritic:2.37e-169 ; Layer1/2:1.56e-190 ; Layer3/4:5.00e-112 ; Layer5:4.34e-99 ; Layer6:8.65e-117 ; Somatic:1.16e-54 |
| 11 | ***Uba52*** | C0,C1,C4,C5,Dendritic,Layer5,Layer6 | C0:5.70e-126 ; C1:9.97e-212 ; C4:9.21e-39 ; C5:4.25e-51 ; Dendritic:3.02e-123 ; Layer5:3.72e-96 ; Layer6:7.14e-90 |
| 12 | ***Eef1a1*** | C0,C3,Dendritic,Layer1/2,Layer3/4,Layer5,Layer6 | C0:6.56e-126 ; C3:1.69e-116 ; Dendritic:1.02e-146 ; Layer1/2:5.12e-218 ; Layer3/4:8.12e-128 ; Layer5:1.59e-139 ; Layer6:5.50e-137 |
| 13 | ***Selenow*** | C0,C1,C3,C5,Layer6 | C0:4.34e-125 ; C1:1.31e-222 ; C3:6.99e-105 ; C5:1.75e-58 ; Layer6:2.34e-115 |
| 14 | ***Fau*** | C0,C1,C3,C4,Dendritic,Layer1/2,  Layer3/4,Layer5,Layer6 | C0:4.48e-125 ; C1:4.15e-151 ; C3:2.94e-78 ; C4:9.53e-50 ; Dendritic:1.17e-142 ; Layer1/2:5.48e-165 ; Layer3/4:4.89e-84 ; Layer5:5.44e-93 ; Layer6:1.96e-113 |
| 15 | ***Cox7c*** | C0,C3,C5,Dendritic,Layer1/2,Layer5,Layer6,Somatic | C0:7.14e-120 ; C3:9.57e-86 ; C5:1.48e-64 ; Dendritic:4.93e-154 ; Layer1/2:3.42e-181 ; Layer5:3.72e-79 ; Layer6:1.79e-98 ; Somatic:3.94e-71 |
| 16 | ***Ubb*** | C0,C1,C2,C3,Dendritic,Layer1/2,  Layer3/4,Layer5,Layer6,Somatic | C0:2.07e-119 ; C1:7.13e-172 ; C2:6.16e-116 ; C3:3.25e-89 ; Dendritic:1.48e-106 ; Layer1/2:4.86e-223 ; Layer3/4:1.03e-112 ; Layer5:4.63e-118 ; Layer6:4.76e-115 ; Somatic:1.08e-60 |
| 17 | ***Hint1*** | C0,C1,C3,C4,C5,Layer1/2,  Layer6,Somatic | C0:1.43e-114 ; C1:1.25e-160 ; C3:3.39e-106 ; C4:8.68e-34 ; C5:1.89e-58 ; Layer1/2:7.73e-165 ; Layer6:2.02e-86 ; Somatic:7.28e-45 |
| 18 | ***Cox6c*** | C0,C1,C3,C5,Dendritic,Layer1/2,  Layer3/4,Layer5,Layer6,Somatic | C0:8.98e-113 ; C1:6.82e-146 ; C3:4.37e-117 ; C5:4.66e-87 ; Dendritic:3.55e-169 ; Layer1/2:6.23e-184 ; Layer3/4:4.97e-124 ; Layer5:1.53e-107 ; Layer6:1.60e-92 ; Somatic:1.40e-85 |
| 19 | ***Gpx4*** | C0,C1,C3,C4,Layer6 | C0:1.45e-110 ; C1:7.58e-175 ; C3:8.63e-78 ; C4:2.16e-21 ; Layer6:1.57e-88 |
| 20 | ***Aldoa*** | C0,C1,C5,Layer1/2,Layer5 | C0:3.66e-110 ; C1:5.83e-163 ; C5:6.22e-68 ; Layer1/2:7.16e-184 ; Layer5:7.02e-73 |
| 21 | ***Pcp4*** | C0 | C0:1.73e-107 |
| 22 | ***Itm2b*** | C0,C1,Layer1/2,Layer6 | C0:6.22e-107 ; C1:6.58e-143 ; Layer1/2:3.61e-151 ; Layer6:2.57e-87 |
| 23 | ***Aplp2*** | C0 | C0:1.25e-106 |
| 24 | ***Cartpt*** | C0 | C0:1.77e-103 |
| 25 | ***Serinc1*** | C0,C1,C2 | C0:2.96e-99 ; C1:1.84e-150 ; C2:7.95e-129 |
| 26 | ***Uqcrb*** | C0,C2,C5,Somatic | C0:1.51e-98 ; C2:1.57e-104 ; C5:5.98e-51 ; Somatic:4.70e-54 |
| 27 | ***App*** | C0,C1,C2,C4,C5,Layer1/2,  Layer3/4,Layer5,Layer6 | C0:8.79e-98 ; C1:3.60e-179 ; C2:1.05e-162 ; C4:2.75e-33 ; C5:2.29e-68 ; Layer1/2:1.42e-154 ; Layer3/4:6.00e-112 ; Layer5:8.80e-79 ; Layer6:2.82e-90 |
| 28 | ***Ddit4*** | C0 | C0:2.14e-97 |
| 29 | ***Ftl1*** | C0,Somatic | C0:8.52e-97 ; Somatic:1.19e-45 |
| 30 | ***Atp5md*** | C0,C2,C3,C5,Dendritic,Layer1/2,  Layer3/4,Layer5,Layer6,Somatic | C0:6.63e-96 ; C2:1.02e-111 ; C3:1.85e-104 ; C5:1.25e-61 ; Dendritic:2.09e-124 ; Layer1/2:4.19e-147 ; Layer3/4:7.96e-84 ; Layer5:5.72e-92 ; Layer6:3.15e-89 ; Somatic:8.28e-70 |
| 31 | ***2900097C17Rik*** | C1 | C1:2.93e-185 |
| 32 | ***Uqcrh*** | C1,C5,Dendritic,Layer5,Somatic | C1:4.49e-171 ; C5:1.63e-54 ; Dendritic:2.70e-97 ; Layer5:7.47e-75 ; Somatic:1.22e-51 |
| 33 | ***Ywhaz*** | C1,C2,C3,Layer3/4,Layer5,Layer6,Somatic | C1:7.27e-166 ; C2:4.60e-120 ; C3:1.95e-75 ; Layer3/4:4.22e-99 ; Layer5:3.36e-80 ; Layer6:3.99e-84 ; Somatic:2.78e-47 |
| 34 | ***Hsp90ab1*** | C1,Layer1/2 | C1:2.16e-162 ; Layer1/2:3.89e-143 |
| 35 | ***Pebp1*** | C1 | C1:3.66e-154 |
| 36 | ***Cfl1*** | C1 | C1:5.12e-153 |
| 37 | ***Ndufa4*** | C1,C3,C5,Dendritic,Layer1/2,  Layer3/4,Layer5,Layer6,Somatic | C1:5.57e-153 ; C3:1.19e-114 ; C5:1.77e-84 ; Dendritic:8.21e-111 ; Layer1/2:3.90e-188 ; Layer3/4:4.96e-94 ; Layer5:1.03e-81 ; Layer6:2.13e-86 ; Somatic:1.23e-56 |
| 38 | ***Ndufb4*** | C1,C2,C3,C4,Dendritic,Layer1/2,Layer5,Somatic | C1:2.36e-150 ; C2:2.07e-125 ; C3:1.57e-85 ; C4:1.89e-19 ; Dendritic:2.71e-122 ; Layer1/2:3.79e-149 ; Layer5:2.53e-79 ; Somatic:4.10e-59 |
| 39 | ***Chchd2*** | C1,C2,C3,C4,Layer1/2 | C1:1.64e-145 ; C2:3.97e-118 ; C3:5.01e-79 ; C4:1.39e-25 ; Layer1/2:5.00e-154 |
| 40 | ***Ndufa13*** | C1,C2,Dendritic | C1:3.80e-144 ; C2:7.74e-118 ; Dendritic:8.32e-92 |
| 41 | ***Snap25*** | C1,C3 | C1:4.32e-143 ; C3:3.69e-80 |
| 42 | ***Calm1*** | C2,C3,Dendritic,Layer3/4,Layer5,Somatic | C2:2.68e-152 ; C3:2.90e-90 ; Dendritic:9.68e-100 ; Layer3/4:5.17e-118 ; Layer5:2.62e-73 ; Somatic:2.24e-64 |
| 43 | ***Ndfip1*** | C2,C4,Layer3/4,Layer5 | C2:3.56e-138 ; C4:2.18e-33 ; Layer3/4:7.73e-89 ; Layer5:1.23e-72 |
| 44 | ***Snhg11*** | C2,Layer1/2 | C2:7.70e-131 ; Layer1/2:8.29e-163 |
| 45 | ***Ndrg4*** | C2,Layer3/4 | C2:2.97e-117 ; Layer3/4:5.46e-101 |
| 46 | ***Vdac1*** | C2 | C2:1.47e-115 |
| 47 | ***Cox7a2*** | C2,C3,C4,C5,Dendritic,Layer1/2,  Layer3/4,Layer5,Layer6,Somatic | C2:3.44e-114 ; C3:9.96e-91 ; C4:8.70e-19 ; C5:3.17e-60 ; Dendritic:1.39e-122 ; Layer1/2:1.65e-183 ; Layer3/4:4.40e-85 ; Layer5:4.22e-88 ; Layer6:1.42e-103 ; Somatic:5.28e-57 |
| 48 | ***Clstn1*** | C2 | C2:2.09e-110 |
| 49 | ***Cox7b*** | C2,C5,Dendritic | C2:1.49e-107 ; C5:1.40e-56 ; Dendritic:1.23e-87 |
| 50 | ***Gabra1*** | C2 | C2:3.15e-104 |
| 51 | ***Slc25a4*** | C2,C5,Layer1/2 | C2:6.76e-104 ; C5:3.09e-55 ; Layer1/2:4.67e-157 |
| 52 | ***Atp5l*** | C2,C5,Dendritic,Somatic | C2:7.29e-104 ; C5:2.21e-59 ; Dendritic:2.61e-97 ; Somatic:6.35e-56 |
| 53 | ***Ndufs5*** | C2 | C2:6.70e-103 |
| 54 | ***Atp5h*** | C2,Dendritic,Layer5 | C2:1.20e-102 ; Dendritic:1.63e-92 ; Layer5:3.86e-74 |
| 55 | ***Vamp2*** | C2,Layer1/2,Layer3/4 | C2:3.56e-102 ; Layer1/2:4.99e-160 ; Layer3/4:5.74e-95 |
| 56 | ***Arf1*** | C2,Layer6 | C2:3.57e-101 ; Layer6:1.29e-87 |
| 57 | ***Atp6v1e1*** | C2 | C2:1.20e-99 |
| 58 | ***Fth1*** | C3,C5,Dendritic,Layer1/2,  Layer3/4,Layer5,Layer6,Somatic | C3:4.31e-158 ; C5:1.18e-71 ; Dendritic:2.26e-202 ; Layer1/2:9.40e-163 ; Layer3/4:1.10e-181 ; Layer5:2.27e-179 ; Layer6:3.53e-118 ; Somatic:2.64e-105 |
| 59 | ***Tmsb4x*** | C3,C4,C5,Dendritic,Layer1/2,  Layer3/4,Layer5,Layer6 | C3:3.55e-148 ; C4:6.80e-40 ; C5:7.81e-66 ; Dendritic:1.05e-98 ; Layer1/2:2.69e-212 ; Layer3/4:2.42e-85 ; Layer5:8.13e-103 ; Layer6:1.12e-164 |
| 60 | ***Bc1*** | C3 | C3:8.10e-133 |
| 61 | ***Dynll1*** | C3,C4,Somatic | C3:2.77e-105 ; C4:6.59e-26 ; Somatic:4.56e-45 |
| 62 | ***Calm2*** | C3,Layer3/4 | C3:1.14e-79 ; Layer3/4:2.77e-117 |
| 63 | ***Gas5*** | C3,Dendritic,Layer6 | C3:1.98e-79 ; Dendritic:3.64e-116 ; Layer6:5.11e-88 |
| 64 | ***Zwint*** | C3,Layer1/2,Layer3/4,Layer5 | C3:2.80e-79 ; Layer1/2:1.19e-149 ; Layer3/4:1.23e-82 ; Layer5:1.26e-75 |
| 65 | ***Atpif1*** | C3,C4 | C3:8.82e-78 ; C4:9.13e-20 |
| 66 | ***Atp1b1*** | C3,C5,Layer3/4,Layer6 | C3:8.21e-76 ; C5:3.37e-66 ; Layer3/4:6.28e-122 ; Layer6:6.09e-82 |
| 67 | ***Rtn3*** | C4,Layer3/4 | C4:2.95e-33 ; Layer3/4:4.52e-88 |
| 68 | ***Olfm1*** | C4,Layer3/4 | C4:3.62e-30 ; Layer3/4:2.65e-90 |
| 69 | ***Tuba1b*** | C4 | C4:6.28e-30 |
| 70 | ***Gnas*** | C4,C5,Dendritic,Layer1/2,  Layer3/4,Layer5,Layer6 | C4:1.25e-29 ; C5:2.12e-57 ; Dendritic:1.39e-106 ; Layer1/2:2.68e-192 ; Layer3/4:1.48e-117 ; Layer5:3.82e-95 ; Layer6:4.05e-98 |
| 71 | ***Hnrnpk*** | C4 | C4:2.57e-29 |
| 72 | ***Ywhah*** | C4,Layer3/4 | C4:1.41e-27 ; Layer3/4:6.96e-84 |
| 73 | ***Pfdn5*** | C4 | C4:2.34e-25 |
| 74 | ***H3f3b*** | C4 | C4:6.17e-24 |
| 75 | ***Basp1*** | C4 | C4:1.15e-20 |
| 76 | ***Atp6v1a*** | C4 | C4:3.83e-20 |
| 77 | ***Ndufa7*** | C4 | C4:4.17e-20 |
| 78 | ***Tle5*** | C4 | C4:3.16e-19 |
| 79 | ***Cox6b1*** | C4,C5 | C4:3.69e-19 ; C5:3.29e-54 |
| 80 | ***Oaz1*** | C4,Somatic | C4:5.29e-18 ; Somatic:5.37e-49 |
| 81 | ***Mt1*** | C5 | C5:4.65e-78 |
| 82 | ***Uqcrq*** | C5 | C5:4.83e-62 |
| 83 | ***Ndufb9*** | C5,Dendritic,Layer5 | C5:1.81e-54 ; Dendritic:1.45e-117 ; Layer5:3.73e-86 |
| 84 | ***Atp5b*** | C5,Dendritic,Layer1/2,  Layer3/4,Somatic | C5:3.60e-53 ; Dendritic:4.23e-98 ; Layer1/2:3.68e-166 ; Layer3/4:1.49e-101 ; Somatic:1.89e-53 |
| 85 | ***Cck*** | C5 | C5:1.74e-50 |
| 86 | ***Atp5j*** | Dendritic | Dendritic:5.01e-99 |
| 87 | ***Ndufa6*** | Dendritic,Layer5,Somatic | Dendritic:6.19e-92 ; Layer5:9.07e-73 ; Somatic:3.52e-54 |
| 88 | ***Psap*** | Layer1/2 | Layer1/2:1.30e-231 |
| 89 | ***Eif1*** | Layer1/2,Layer5 | Layer1/2:6.64e-154 ; Layer5:9.84e-75 |
| 90 | ***Rtn1*** | Layer3/4 | Layer3/4:7.03e-103 |
| 91 | ***Atp5a1*** | Layer3/4,Layer6,Somatic | Layer3/4:3.65e-92 ; Layer6:9.37e-91 ; Somatic:3.89e-47 |
| 92 | ***Bex2*** | Layer3/4,Layer5 | Layer3/4:1.00e-88 ; Layer5:1.55e-72 |
| 93 | ***Sparcl1*** | Layer6 | Layer6:3.18e-87 |
| 94 | ***Gpm6a*** | Layer6 | Layer6:1.81e-82 |
| 95 | ***Scg5*** | Layer6 | Layer6:3.85e-82 |
| 96 | ***Timm8b*** | Somatic | Somatic:2.49e-54 |
| 97 | ***Wbp11*** | Somatic | Somatic:3.05e-48 |
| 98 | ***Sumo2*** | Somatic | Somatic:6.76e-47 |
| 99 | ***Ptk2b*** | Somatic | Somatic:2.27e-45 |
| 100 | ***Atp5mpl*** | Somatic | Somatic:3.98e-45 |
| 101 | ***Atp5j2*** | Somatic | Somatic:1.26e-44 |
